# Design of a multi-target focused library for antidiabetic targets using a comprehensive set of chemical transformation rules

**DOI:** 10.3389/fphar.2023.1276444

**Published:** 2023-11-02

**Authors:** Fernanda I. Saldívar-González, Gabriel Navarrete-Vázquez, José L. Medina-Franco

**Affiliations:** ^1^ Department of Pharmacy, DIFACQUIM Research Group, School of Chemistry, Universidad Nacional Autónoma de México, Mexico City, Mexico; ^2^ Facultad de Farmacia, Universidad Autónoma Del Estado de Morelos, Cuernavaca, Morelos, Mexico

**Keywords:** computer-aided drug design, diabetes, target-focused libraries, library enumeration, ligandbased design, multi-target design, transformation rules, reaction informatics

## Abstract

Virtual small molecule libraries are valuable resources for identifying bioactive compounds in virtual screening campaigns and improving the quality of libraries in terms of physicochemical properties, complexity, and structural diversity. In this context, the computational-aided design of libraries focused against antidiabetic targets can provide novel alternatives for treating type II diabetes mellitus (T2DM). In this work, we integrated the information generated to date on compounds with antidiabetic activity, advances in computational methods, and knowledge of chemical transformations available in the literature to design multi-target compound libraries focused on T2DM. We evaluated the novelty and diversity of the newly generated library by comparing it with antidiabetic compounds approved for clinical use, natural products, and multi-target compounds tested *in vivo* in experimental antidiabetic models. The designed libraries are freely available and are a valuable starting point for drug design, chemical synthesis, and biological evaluation or further computational filtering. Also, the compendium of 280 transformation rules identified in a medicinal chemistry context is made available in the linear notation SMIRKS for use in other chemical library enumeration or hit optimization approaches.

## 1 Introduction

Type 2 diabetes mellitus (T2DM) is a metabolic disorder characterized by hyperglycemia caused by defects in insulin secretion and/or action due to a complex network of pathological conditions (Galicia-Garcia et al., 2020). Currently, T2DM is one of the diseases with the highest socio-health impact and prevalence worldwide. Although pharmacotherapeutic options include different mechanisms of action, they are limited by side effects and lack of blood glucose control in the diabetic population (Shah et al., 2021). Another problem is that patients with this disease are prone to polypharmacy, which increases the risk of adverse effects and makes it difficult for patients to adhere to their treatment and receive proper follow-up from healthcare professionals (Dobrică et al., 2019). For this reason, new biological targets have been explored in multi-target approaches (Makhoba et al., 2020). Similarly, virtual libraries focused on single therapeutic targets have been developed using various computational approaches and their application in multi-target approaches is emerging.

Recent advances in computational methods and the incorporation of synthetic expert knowledge have inspired research groups to develop *de novo* and “make-on-demand” chemical libraries (Walters, 2019). Several companies use the so-called “novel molecular matter” in early-phase drug discovery (Korn et al., 2023). Specifically in the design of antidiabetic compounds, Otava released the chemical structures of ten libraries focused on DM-related targets designed under ligand- and structure-based approaches or combinations of both (OTAVAchemicals, Ltd. - synthetic organic compounds for research and drug discovery, n.d.). ChemDiv developed methods for screening diverse and highly specialized focused compounds. Recently, ChemDiv released an “Annotated space library” with more than 18,000 chemical compounds covering 38 validated targets (including targets for T2DM) across 900 drugs launched in the last 10 years. Academic groups have also generated virtual libraries focused on T2DM. For example, Chen et al. used a generative method to design compounds targeted for GPR40 (Chen et al., 2021). To our knowledge, no *in silico* multi-target libraries have been disclosed for T2DM. However, there is published information on active compounds and pharmacophore models that can guide the design of multi-target compounds for T2DM (Artasensi et al., 2020; Lillich et al., 2020; Tassopoulou et al., 2022).

**FIGURE 1:**
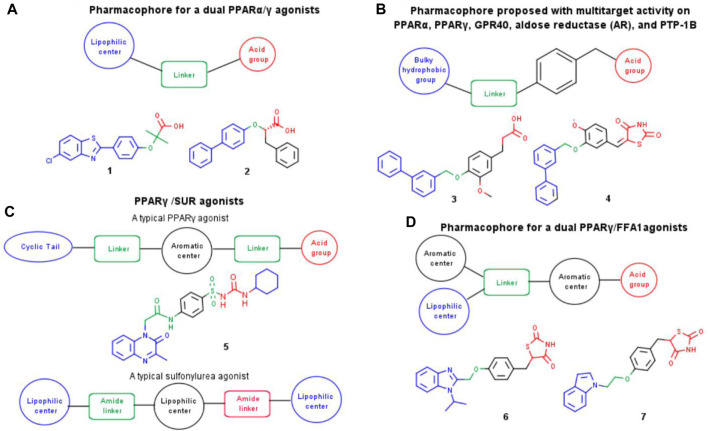
Pharmacophores and chemical structures of multi-target compounds studied in *vivo* models for T2DM and MEtS. **(A)** PPARα/γ agonists, **(B)** PPARα, PPARγ, GPR40, AR and PTP1B **(C)** PPARγ/SUR agonists **(D)** PPARγ/FFA1 agonists.

Figure 1 shows the structures of pharmacophores and multi-target compounds studied *in vivo* models for T2DM and metabolic syndrome (MetS). For example, dual peroxisome proliferator-activated receptor (PPAR) α/γ agonists can improve insulin sensitivity and reduce triglyceride and blood glucose levels without the PPARγ-related weight gain since the latter effect is balanced by PPARα agonistic activity (Balakumar et al., 2019). Attempts towards developing dual agonists for PPAR α/γ yielded promising molecules that have reached clinical trials. However, many of these compounds have failed due to significant side effects. Compounds such as 1 (MHY908) and 2 (LT175) continue to be explored, as they have been shown to have beneficial effects on blood glucose and insulin resistance in animal models of T2DM (Gilardi et al., 2014; Park et al., 2016).

The development of multi-target compounds for GPR40, PTP1B, AR, PPARα, and PPARγ may provide additional therapeutic benefits in preventing or delaying the development of diabetic complications (Domínguez-Mendoza et al., 2021). Navarrete’s group designed compounds 3 and 4 (Figure 1) that showed robust *in vivo* antihyperglycemic activity (Domínguez-Mendoza et al., 2021). Dual PPARγ/SUR agonists, such as compound 5 (Ibrahim et al., 2017a), can improve insulin sensitivity and stimulate insulin secretion simultaneously, making them an attractive therapeutic option for patients with T2DM who have insulin resistance and decreased insulin secretion. Another combination that has been explored is that of PPARy and FFA1, also known as GPR40. Dual PPARγ/FFA1 agonists (compounds 6 and 7, Darwish et al., 2018) can improve insulin sensitivity, stimulate insulin secretion, and lower blood glucose levels.

Table 1 shows multi-target compounds for T2DM based on pharmacophores and virtual screening approaches that have been reported and tested *in vivo* and can guide the design of new chemical libraries. The table includes information on the combination of targets, the number of lead compounds that hit all targets, and the implications or outcome in T2DM and MEtS. The table indicated that there are ninety-one multi-target compounds with *in vivo* antidiabetic activity. These have been tested in a total of twenty target combinations. The most studied biological target in multi-target approaches is PPARγ. The most successful target combination is PPARα/γ as several compounds directed to these two targets are in clinical studies such as ragaglitazar, imiglitazar, muraglitazar, tesaglitazar, naveglitazar, aleglitazar, saroglitazar, netoglitazone and lobeglitazone. In particular, PPARs have demonstrated clinical efficacy in metabolic diseases such as T2DM and those related to lipid metabolism, which has led to testing and optimization of different compounds such as fibrates and TZDs to simultaneously modulate multiple targets, with a synergistic effect on T2DM and MetS (Ammazzalorso et al., 2019). Other compounds under investigation or in any of the experimental phases by the FDA include elafibranor for PPARα/δ (Schattenberg et al., 2021), telmisartan and fimasartan for PPARγ/AT1 (Seo et al., 2022), lanifibranor and sodelglitazar for PPARα/δ/γ (Kamata et al., 2023) and licogliflozin and sotagliflozin for SGLT1-2, the latter already approved by the FDA in 2023 to reduce the risk of cardiovascular death and heart failure in adults with heart failure, T2DM, chronic kidney disease, and other cardiovascular risk factors (Packer, 2023).

**TABLE 1:** Multi-target ligands studied *in vivo* models for type II diabetes mellitus.

Targets	Lead compounds	Implications in T2DM and MEtS/Outcome	Reference
GLUT4, PPAR-α, PPAR-γ, adiponectin	1	Antihyperglycemic, antidiabetic, and antidyslipidemic effects	Estrada-Soto et al. (2022)
PPARα, PPARγ, GPR40*, AR, PTP1B	2	Antihyperglycemic and antidiabetic effects. Attractive to prevent or delay the development of diabetic complications	Colín-Lozano et al. (2018), Domínguez-Mendoza et al. (2021)
PPARγ, GLUT-4	3	Antihyperglycemic and antidiabetic effects	Gutierréz-Hernández et al. (2019)
PPARα, PPARγ, FATP-1, GLUT-4, PTP1B	4	Antihyperglycemic and antidiabetic effects	Herrera-Rueda et al. (2018)
sEH,PPARγ	2	Antidiabetic, cardioprotective, renoprotective, hypotensive effects	Blöcher et al. (2016), Hye Khan et al. (2018)
DPP-4,GPR119	2	Antidiabetic, glucose homeostasis effects	Huan et al. (2017), Fang et al. (2020)
PPAR-α,γ	21	Antidiabetic and antidyslipidemic effects (13 PPARα/γ dual agonist compounds have reached clinical trials or the market)	Balakumar et al., 2007 (2019), Ammazzalorso et al. (2019)
PPAR-α,d	3	Antidiabetic, antidyslipidemic and anti-fatty liver effects	Hanf et al. (2014), Ren et al. (2020), Liu et al. (2021)
PPAR-d,γ	1	Antihyperglycemic and anti-fatty liver effects	Li et al. (2021)
PPAR-α,d,γ	6	Antidiabetic and antidyslipidemic effcts. Therapeutic potential for nonalcoholic steatohepatitis patients	Mahindroo et al. (2005), He et al. (2012), Boubia et al. (2018)
PPARγ, AT1	6	Antidiabetic and antihypertensive effects	Benson et al. (2004), Casimiro-Garcia et al. (2011), 2013; Lamotte et al. (2014), Choung et al. (2018)
PPARγ, GK	7	Antihyperglycemic, antidiabetic, improves insulin resistance and sensitize muscle cells to insulin response	Song et al. (2011), Li et al. (2014), Lei et al. (2015)
PPARγ, SUR	10	Improve insulin sensitivity and stimulate insulin secretion at the same time	Ibrahim et al., 2017a, 2017b
FFA1^a^, PPARd	5	Antidiabetic and anti-fatty liver effects	Li et al., 2019a, 2019b (2020), Hu et al. (2020), Zhou et al. (2022)
FFA1, PPARγ	4	Antidiabetic and antihyperlipidemic effects	Darwish et al. (2018), Hidalgo-Figueroa et al. (2021)
FFA1, PPARγ, PPARd	1	Antidiabetic and antihyperglycemic effects	Li et al. (2018)
PPARγ, PTP1B	4	Antihyperglycemic and antiobesity effects	Bhattarai et al., 2009 (2010), Otake et al., 2012 (2015)
PPARα/γ/PTP1B	2	Antidiabetic, antidyslipidemic and antiobesity effects	Otake et al. (2011)
PARP-1 - AR	2	Nephroprotective effect and antioxidant potential	Chadha and Silakari (2017), Kumar et al. (2022)
SGLT1-SGLT2	5	Antihyperglycemic and antiobesity effects	Lapuerta et al. (2015), Kuo et al. (2018), Xu et al., 2018 (2020), He et al. (2019)

AT1, angiotensin II type 1 receptor; AR, aldose reductase; DPP-4, dipeptidyl peptidase-4; FATP-1, fatty acid transport protein 1; FFAR1, free fatty acid receptor 1; GK:glucokinase; GLUT4, glucose transporter type 4; GPR119, G protein-coupled receptor 119; GPR-40, G-protein-coupled receptor 40; PARP-1, poly (ADP-ribose)polymerase-1; PPARs, peroxisome proliferator-activated receptors; PTP1B, protein tyrosine phosphatase 1B; SGLTs: ; sEH, soluble epoxide hydrolase; SUR, sulfonylurea receptor.

^a^ GPR40 is also known as FFAR1.

An attractive approach to exploring and expanding the chemical space around the first hit compounds of single and multi-target compounds is the computational generation of chemical libraries that can be used in virtual screening campaigns (Walters, 2019). Through chemical library enumeration, it is possible to find new bioactive compounds and generate therapeutic options for emerging and challenging molecular targets and complex diseases. It is also possible to control features such as library size, complexity, physicochemical properties, and structural diversity (Ruddigkeit et al., 2012). The goal is to help design high-quality analog series overcoming issues such as low potency, off-target activities, metabolic stability, or poor physicochemical properties for oral administration. One approach that can aid rigorous exploration of the chemistry around first hit compounds is using approaches based on transformation rules from empirical observation and systematic identification using chemoinformatics methods. A recent application of transformation rules was presented with DrugSpaceX, a database with more than 100 million compounds transformed from approved drug molecules (Yang et al., 2021). Although transformation rules are useful for generating *in silico* libraries, the list of rules currently available in the public domain is limited, in many cases due to the difficulty in collecting, curating, and annotating such information (Rarey et al., 2022). To address this problem, we compiled, organized, and made freely available an extensive list of transformation rules for generating compound libraries, as part of this work.

**FIGURE 2:**
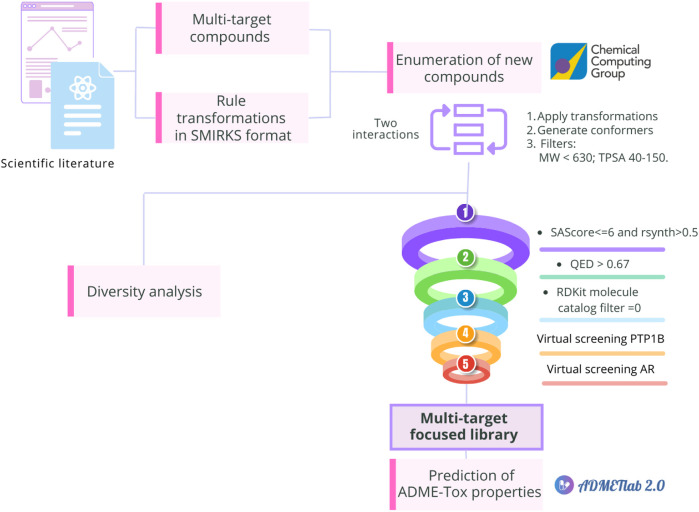
Overview of the methodology implemented in this study to design a multi-target focused library for T2DM using a comprehensive set of chemical transformation rules. First, multi-target compounds evaluated for *in vivo* antidiabetic activity and transformation rules were retrieved from the literature. The latter were encoded into SMIRKS linear notation. Using MOE, the new chemical library was enumerated and compared to reference libraries of antidiabetic compounds to assess their chemical diversity. The compounds in the enumerated library were filtered using criteria as follows: QED > 0.67, SAScore ≤ 6, rsynth > 0.5 and zero structural alerts included in the RDKit molecule catalog filter node in KNIME. These compounds were further filtered in docking-based virtual screening with protein tyrosine phosphatase 1B (PTP1B) and aldose reductase (AR). Finally, the ADME-T properties of the best-scoring compounds in both targets were calculated.

This study aimed to design a multi-target focused library for T2DM using a comprehensive set of chemical transformation rules herein collected, curated, and annotated. As shown in Figure 2, we evaluated the novelty and diversity of the focused library by comparing it with antidiabetic compounds approved for clinical use, natural products, and multi-target compounds reported with *in vivo* activity. To focus the library on attractive and synthetically feasible compounds, computational filters based on medicinal chemistry criteria were applied. Finally, virtual screening using molecular docking for PTP1B and AR was performed at Molecular Operating Environment (MOE) version 2022.02 (Chemical Computing Group (CCG), 2023) and the ADME-Tox properties of compounds with potential multi-target activity were calculated using ADMElab 2.0 (Xiong et al., 2021). Here, we selected PTP1B and AR considering the reference compounds used for enumeration and the currently available hypotheses and information from molecular dynamics models (Domínguez-Mendoza et al., 2021). In particular, this combination could be attractive to modulate insulin signaling, reducing insulin resistance and preventing or delaying diabetic complications such as nephropathies, neuropathies, and cardiomyopathies (Maccari and Ottanà, 2015). The significance of this work is twofold: making available a focused multi-targeted library for T2DM with full details of the methodology used to construct the compounds and making publicly available a general and extensive list of transformation rules to explore the chemical space of targets of therapeutic relevance.

## 2 Methods

### 2.1 Encoding chemical transformations in linear notation (SMIRKS format)

Transformation rules associated with hit optimization were retrieved from the literature. These transformation rules included modifications associated with molecular, physicochemical, pharmacological, ADME, safety, and toxicity parameters. Other rules are associated with structural diversity and bioisosteric changes. Most of the transformation rules found in the literature come from the addition, substitution or removal of a functional group. There are also rules that include cyclization and ring substitution by other rings or intramolecular hydrogen bonding groups. In total, 280 transformation rules were collected and converted into SMIRKS notation (Daylight theory: SMIRKS - A reaction transform language, 2023) using MarvinSketck (Chemaxon, 2023). The transformation rules were considered in a protonation state of 7.4. Table 2 summarizes examples of the rules identified and their SMIRKS. The Supplementary material includes the complete list of transformation rules and the literature reference.

**TABLE 2:** Examples of transformation rules retrieved from the literature.

Group	Transformation	Transformation type	SMIRKS	Note	Reference
Aminophenyl	Aminophenyl_to_aminobicyclo[1.1.1]pentyl 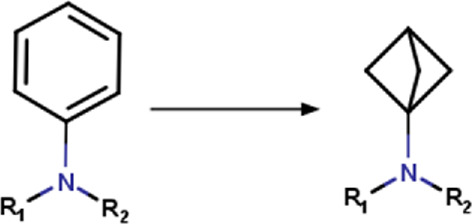	Ring substitution	[*:3]-[#7:2](-[*:1])-[c:4]1[c; D2][c; D2][c; D2][c; D2][c; D2]1>>[*:3]-[#7:2](-[*:1])[C:4]12[#6]-[#6](-[#6]1)-[#6]2	-Metabolic stability	Sodano et al. (2020)
				-Isosteric replacement	
1,4-diaminophenyl	1,4-diaminophenyl_to_3,6-diaminopyridazinyl 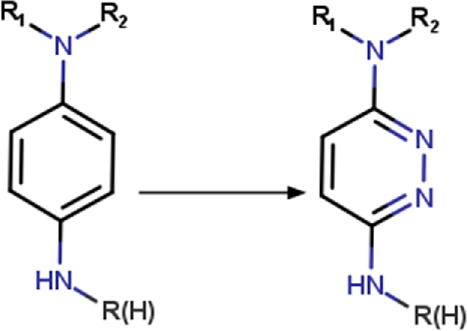	Ring modification	[#7H2:1]-[c; D3:2]1[c; D2:6][c; D2:5][c; D3:4][c; D2:3][c; D2]1>>[#7H2:1]-[c; D3:2]1[c; D2:6][c; D2:5][c; D3:4]nn1	-Metabolic stability	Zhang et al. (2020)
Carboxyl	Carboxyl_to_2,4-dioxo-1,3-thiazolidin-5-yl (ionized) 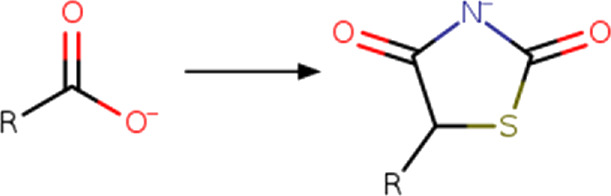	Ring addition	[#8; D1][#6; A; !R:2]([*:1]) = O>>[*:1]-[#6:2]-1-[#16]-[#6](=O)-[#7-]-[#6]-1 = O |s:0:1|	-Similar acidic pKa	Hidalgo-Figueroa et al. (2013), Domínguez-Mendoza et al. (2021)
				-Increased sterics	
Benzoylphenyl	benzoylphenyl _to_(3-phenyloxetan-3-yl)phenyl] 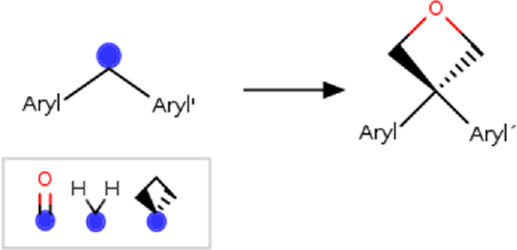	Linker modification	[#6; a:1][C; $([#6:2] = O),$([#6; A; @:2]1[#6]-[#6]-[#6]1),$([#6; H2])][#6; a:3] >>[#6; a:3][#6; A@:2]1([#6; a:1])[#6]-[#8:4]-[#6]1	-Isosteric replacement	Dubois et al. (2021)
			*Also apply to 1,1-diphenylmethyl and 1.1′-cyclobutane-1,1-diyldiphenyl	-Metabolic stability	
				-Reduce phototoxicity in benzofenones	
Phenyl	Phenyl_to_cyclohexyl 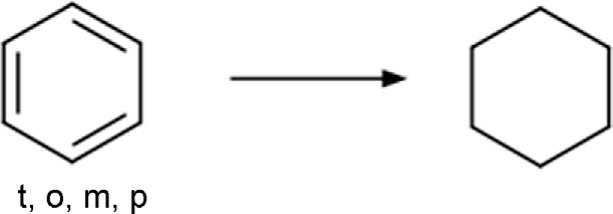	Ring substitution	[c; x2:2]1[c; x2:3][c; x2:4][c; x2:5][c; x2:6][c; x2:1]1>>[#6:5]-1-[#6:6]-[#6:1]-[#6:2]-[#6:3]-[#6:4]-1	-Bioisosteric remplacement	Press et al., 2012; Press et al., 2015; Huang et al., 2019; Subbaiah and Meanwell, 2021
				-Increase lipophilicity	
				-Improved aqueous solubility	
				-Enhanced oral bioavailability	
				-Reproducible PK profiles	
Phenyl	Phenyl_to_(propoxyimino)methyl 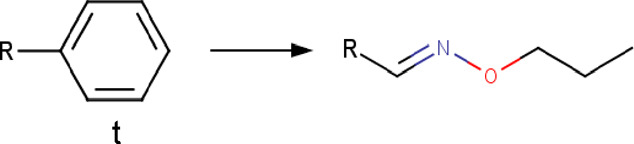	Functional group change	[*:3]-[c; x2D3:1]1[c; x2D2][c; x2D2][c; x2D2][c; x2D2][c; x2D2]1>>[#6]-[#6]-[#6]-[#8]\[#7] = [#6:1]\[*:3] |rb:1:2.2:2.3:2.4:2.5:2.6:2,s:1:3|	-Bioisostere replacement	Piemontese et al. (2015), Subbaiah and Meanwell (2021)
				-Modulation of selectivity	
Phenyl	Phenyl_to_(2-oxopyridin-1(2H)-yl) 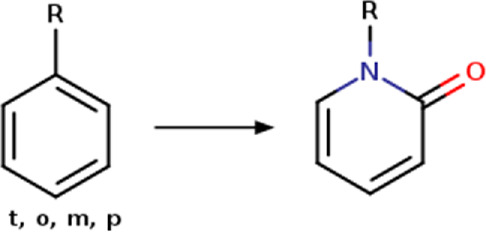	Ring modification	[c; D2:2]1[c; x2:3][c; x2:4][c; x2:5][c; x2:6][c; D3:1]1>>O = [#6; x2:2]-1-[#7:1]-[#6:6] = [#6:5]-[#6:4] = [#6:3]-1	-Improved potency	Subbaiah and Meanwell (2021)
				-Reduce off-target	
				-Metabolic stability	
				-Enhanced solubility	
				-Reduced lipophilicity	

### 2.2 Enumeration of a new multi-target focused library

Compounds **3** and **4** (Figure 1) were selected as reference compounds to enumerate the new library focused on multi-target compounds. These compounds have robust antihyperglycemic activity *in vivo* and molecular dynamics studies with PTP1B and AR provide relevant structure ligand-protein interaction information for structure-based optimization studies (Domínguez-Mendoza et al., 2021). The best predicted scoring conformations of these compounds docked with a crystallographic structure of the PTP1B protein obtained from the Protein Data Bank (Berman et al., 2000) (PDB ID: 4Y14 (Krishnan et al., 2015)) and that maintained protein-ligand interactions reported in literature were used as the basis for the enumeration of the new library. To compare the effect on the number of compounds and the molecular diversity generated, we used the 175 transformation rules integrated into the MedChem module of MOE version 2022.02 and added 273 transformation rules that we constructed as part of this study. Two iterations were used. Compounds that had a molecular weight (MW) < 630 and topological surface area (TPSA) between 40 and 150 Å were kept. The threshold values were established based on the minimum and maximum values of the multi-target compounds designed for T2DM.

### 2.3 Evaluation of the chemical diversity of the multi-target focused library

The compounds generated in Section 2.2 were compared in terms of physicochemical properties with antidiabetic compounds approved for clinical use retrieved from DrugBank (Wishart et al., 2018); antidiabetic compounds from medicinal plants obtained from DiaNat-DB (Madariaga-Mazón et al., 2021), and multi-target compounds for T2DM studied *in vivo* assays. Compounds in SMILES format can be found in the Supplementary material. Prior to analysis, each molecule was prepared using the open-source cheminformatics toolkits RDKit (Landrum, n.d.) and MolVS (MolVS: Molecule Validation and Standardization—MolVS 0.1.1 documentation, n.d.). Compounds were standardized and those containing multiple components were split, keeping the largest component. Compounds with valence errors or chemical elements other than H, B, C, N, O, F, Si, P, S, Cl, Se, Br, and I were removed. The remaining compounds were neutralized and reionized to subsequently generate a canonical tautomer. Finally, duplicated compounds were deleted and canonical simplified molecular-input line-entry system (SMILES) (ignoring stereochemistry information) were generated as molecular representation (Weininger, 1988). Table 3 summarizes the number and source of compounds used in the comparison.

**TABLE 3:** Reference compound datasets to assess the novelty and properties of the newly designed libraries.

Database	Size	Note	Reference
Approved antidiabetic drugs	42	36 approved drugs and 6 under investigation. Obtained from DrugBank. Only compounds for a single target.	Wishart et al. (2018)
DiaNat -DB	329	Antidiabetic compounds from medicinal plants	Madariaga-Mazón et al. (2021)
Multi-target compounds	91	From literature. This set includes 16 multi-target compounds under investigation or in experimental phase by FDA.	This study. See Section 2.1
Multi-target compMedChem	84,778	Compounds generated in MOE using 455 transformations rules	This study. See Section 2.3

#### 2.3.1 Property rules and synthetic accessibility

To profile the generated molecules based on common property rules and synthetic accessibility, we calculated seven descriptors for each molecular entity with the RDKit library: molecular weight (MW), octanol–water partition coefficient (logP), number of hydrogen bond acceptors (HBA), number of hydrogen bond donors (HBD), topological polar surface area (TPSA), the number of rotatable bonds (RotB) and the distribution of quantitative estimate of drug-likeness (QED) (Bickerton et al., 2012).

Among the several computational scores available to estimate the synthetic accessibility of generated compounds, and based on previous comparisons of scores. (Skoraczyński et al., 2023), in this work, we calculated rsynth (a ligand-based score implemented in MOE, version 2022.02) and SASscore (Ertl and Schuffenhauer, 2009) as a structure-based approach.

#### 2.3.2 Chemical multiverse: visual representation and analysis

To have a comprehensive analysis of the chemical space, we used three well-established visualization methods and different types of descriptors Noteworthy, analyzing the chemical space with distinct and complementary descriptors is crucial because each one provides a different and complementary perspective of the chemical space (aka, a “chemical multiverse” as discussed elsewhere (Medina-Franco et al., 2022). In this study, we used principal component analysis (PCA) and t-distributed stochastic neighbor embedding t-SNE based on six molecular properties of pharmacological interest, namely,; HBD, HBA, logP, MW, RB, TPSA. As a third method to characterize the chemical space, we used was the Tree MAP (TMAP) algorithm (Probst and Reymond, 2020) based on ECFP4 as a structural fingerprint. Additionally, the molecular shape of the compounds in the newly generated library was evaluated using the principal moments of inertia (PMI) graph, which was carried out by calculating the lowest energy conformation of each compound using MMFF94x as a force field in MOE, version 2022.22. Once the lowest energy conformer was calculated, values of normalized PMI ratios, npr1 (I1/I3) and npr2 (I1/I3), were determined in MOE. Then, npr1 and npr2 were plotted on a triangular graph with the vertices (0,1), (0.5,0.5), and (1,1) representing a perfect rod, disc, and sphere, respectively.

### 2.4 Filtering of compounds with relevance in pharmaceutical chemistry

Various filters (e.g., calculated descriptors) can be used to improve the selection of enumerated compounds, including the removal of compounds with undesirable pharmaceutical properties and molecules very difficult to synthesize. In this work, we chose to filter by SAScore, rsynth, QED, and the filters included in the RDKit molecule catalog filter node in KNIME. This node removes compounds with Pan-assay interference compounds (PAINS) (Baell and Holloway, 2010), unwanted functionality due to potential toxicity or unfavorable pharmacokinetic properties, and problematic functional groups. The compounds in the enumerated library were filtered using criteria as follows: QED > 0.67, SAScore ≤ 6, rsynth >0.5 and zero structural alerts. It should be noted that other cutoff values could be used for other applications.

### 2.5 Structure-based filtering

Although enumeration of compounds can be performed using the protein structure to obtain a score value in MOE, this can be computationally expensive considering the number of compounds that can potentially be generated. In addition, although the search algorithm can be selected, neither an algorithm to perform the rescoring nor a specific number of conformations to be obtained can be selected. For this reason, we followed a structure-based filtering to select the most promising compound subset as a multi-target library. In this study, compounds that showed a docking score better than the reference compounds for PTP1B (PDB ID: 4Y14 (Krishnan et al., 2015)), were further filtered considering docking with another therapeutic target relevant to T2DM: AR (PDB ID: 4XZH (Ruiz et al., 2015)). Of note, other relevant targets in T2DM can be considered during the design of the reference compounds. Here, we selected PTP1B and AR considering the current available hypothesis and model information based on molecular dynamics (Domínguez-Mendoza et al., 2021). Also, we want to point out that several other docking programs could be used (including post consensus scoring analysis). However, testing different docking programs and exploring multiple consensus analysis schemes is out of the scope of this study that is focused on proposing a general approach to design multi-target focused libraries.

The protein preparation of PDB ID: 4Y14 and 4XZH was made with default settings of the QuickPrep module of MOE v. 2022.02. This module carries out the following processes: calibration of the structure by protonation, addition of all the lacking hydrogen atoms, elimination of water molecules 4.5 Å farther from the protein, addition of missing amino acids residues, neutralization of the endpoints adjoining empty residues and energy minimization. We used AMBER14:EHT forcefield (ff14SB (Maier et al., 2015) as parameter for the energy minimization stage for the protein. The ligands were also prepared in MOE, we used MFF94x as forcefield. For docking, the receptor was considered rigid and the ligands flexible. We used the default settings for placement (method: triangle matcher, score function: London dG) and refinement (method: rigid receptor, score function: GBVI/WSA dG) (Vilar et al., 2008).

### 2.6 Prediction of ADME-Tox properties

The ADME-tox properties of the compounds that had successfully passed the filters described in Section 2.6 and Section 2.7 were evaluated using ADMETlab 2.0 platform (Xiong et al., 2021). This platform has been compared with other free online ADMET tools and has significant advantages (Dulsat et al., 2023). Based on these comparisons we used this tool because ADMETlab is a complete platform in terms of the large number of relevant parameters that can be predicted including elimination parameters such as clearance and half-life (t1/2). The latter two descriptors are particularly relevant in chronic diseases such as T2DM, where ideally compounds with a long half-life are sought to reduce the dose. Finally, ADMETlab 2.0 allows evaluating up to 500 compounds at the same time.

## 3 Results and discussion

### 3.1 Encoding chemical transformations in SMIRKS format

Based on the structures exploited in the multi-target pharmacophores for DMT2, 280 transformation rules were collected from the literature, of which 113 were bioisosteric modifications of the phenyl group (Subbaiah and Meanwell, 2021), 25 for the amide bond (Kumari et al., 2020), 36 for the carboxyl group (Bredael et al., 2022), 57 for the phosphate group, and 49 for other moieties including ester, alcohol, alkyl, aminophenyl, and nitro to name a few examples. Compared to the preloaded MOE transformations (version 2022.22), these included only 10 for the phenyl group, 7 for the amide bond and 4 for the carboxyl group. The remaining transformations in MOE mainly concern cyclization and substitution of rings by other rings or intramolecular hydrogen bonding groups. It is important to mention that some examples of the rule transformations present in Table 2 have been applied in the optimization of antidiabetic compounds. For example, Huang et al. reported the effect of bioisosteric replacement of a phenyl ring in the biphenyl moiety with cyclohexyl motif in a GPR40 agonist (Huang et al., 2019). Another bioisosteric replacement of the phenyl ring in an antidiabetic compound was reported by (Piemontese et al., 2015). In that work, the authors replaced a terminal phenyl ring in a dual PPAR α/γ agonist with the n-propyl oxime moiety. For its part, Dr. Navarrete-Vázquez’s group has proposed various series of thiazolidine-2,4-dione and barbituric acid derivatives with robust antihyperglycemic activity *in vivo* as acid bioisosteres (Hidalgo-Figueroa et al., 2013; Domínguez-Mendoza et al., 2021).

### 3.2 Enumeration of new compounds

Using the transformation rules preloaded in MOE and performing two iterations, 6,838 molecules were obtained from compound **3** and 1834 from compound **4**. After adding the transformation rules collected from the literature and keeping two iterations, the number of compounds increased to 52,185 (from compound **3**) and to 32,593 (from compound **4**), after curation of the enumerated library. This large increase in the number of compounds was expected, as it followed an iterative process, and the transformations considered in this work are focused on moieties that contain the reference compounds.

### 3.3 Evaluation of the chemical diversity of the multi-target focused library

#### 3.3.1 Property rules and synthetic accessibility

**FIGURE 3:**
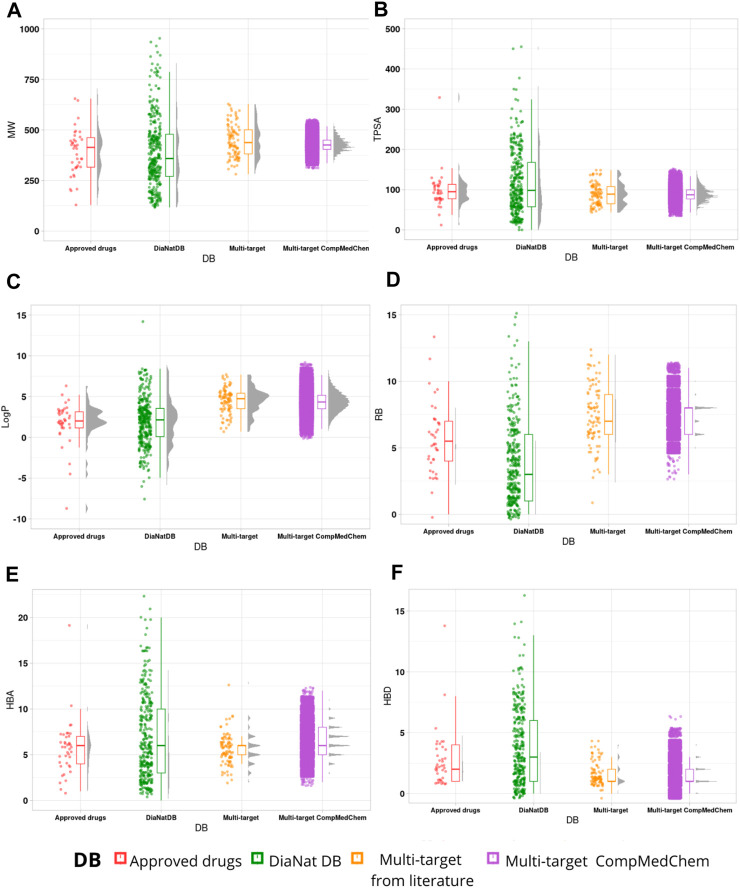
Rain cloud plots of the six physicochemical properties of pharmaceutical relevance: **(A)** molecular weight (MW), **(B)** topological polar surface area (TPSA), **(C)** partition coefficient octanol/water (log P), **(D)** number of rotatable bonds (RB), **(E)** hydrogen bond acceptors (HBA) and **(F)** hydrogen bond donors (HBD).

Figure 3 shows the distributions of each descriptor for the compounds of the generated library and the reference libraries using rain cloud plots (Allen et al., 2019). These plots allow visualization of the probability density and typical boxplot statistics such as median, mean, and confidence intervals. According to these plots, DiaNat-DB (329 antidiabetic compounds from medicinal plants) has a wider distribution in terms of properties of pharmaceutical importance. Since MW and TPSA values were used as filters to generate the new compounds, the distributions of these values for generated compounds are skewed and resemble the distribution of multi-target compounds and approved drugs. Comparing the plots of the other descriptors, we can see that the transformations used increased the range of properties such as LogP and HBD, HBA and chirality (Supplementary Table S1 in the Supplementary Material).

**FIGURE 4:**
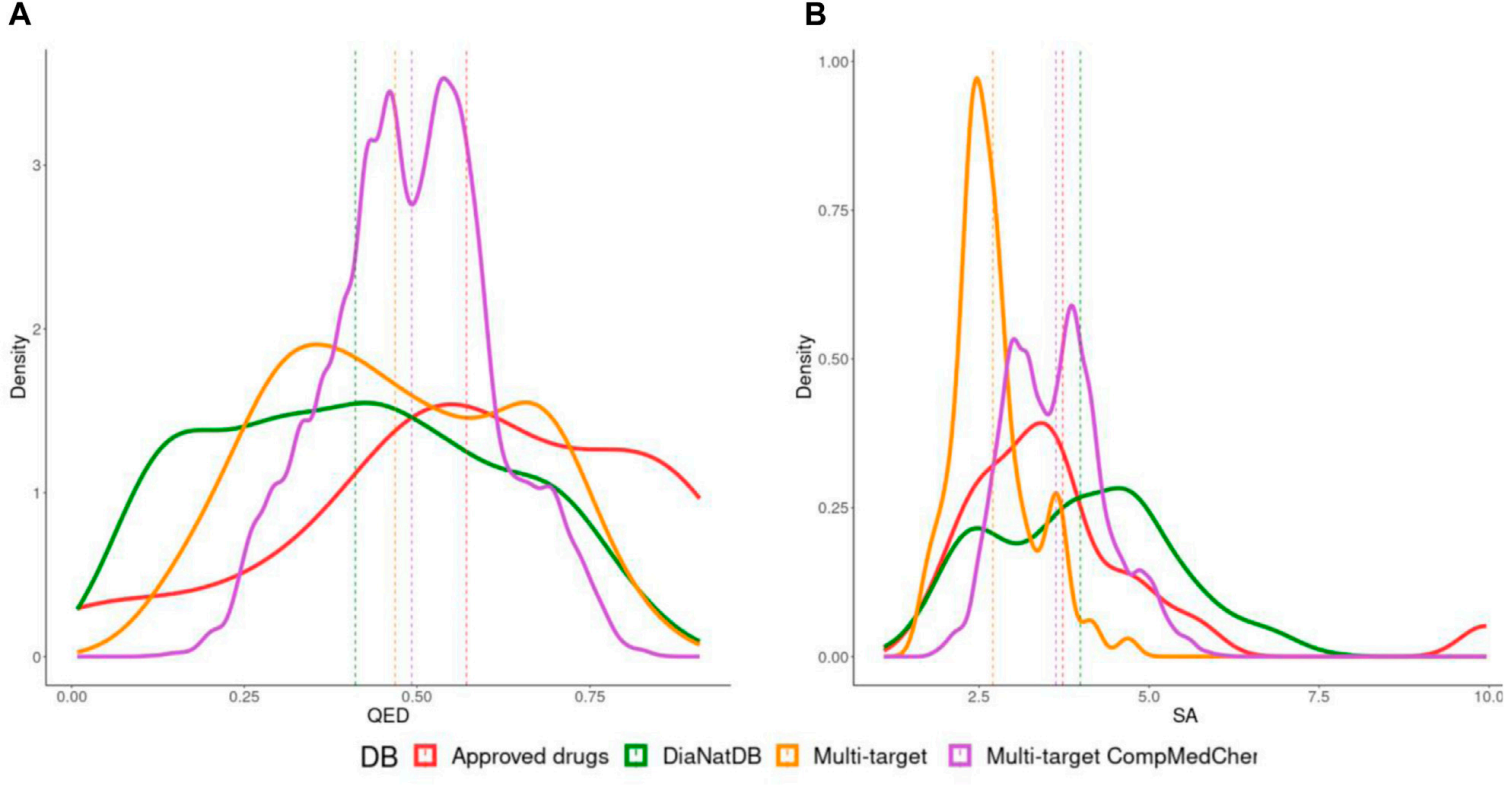
Distribution of **(A)** quantitative estimate of drug likeness (QED) and **(B)** SAscore of all the antidiabetic compounds contained in approved drugs (red) DiaNat database (green), multi-target compounds (orange) and multi-target compounds generated by medicinal chemistry transformation rules (purple). Vertical dashed lines represent the mean of the distributions.

Figure 4 shows the distributions of the calculated descriptors to quantity synthetic accessibility and drug-likeness through QED. As can be seen in Figure 4, most of the generated compounds have a SAScore value of less than 6, indicating that they are, in principle, synthesizable (Ertl and Schuffenhauer, 2009). Regarding the QED value, which is a measure of drug-likeness based on the concept of desirability, a value greater than or equal to 0.67 represents attractive compounds, and the lower this value, the compounds are considered unattractive (QED 0.49∼0.67) and too complex (QED < 0.49). These reference values were established based on the calculated physicochemical properties of marketed oral drugs and published human data (Bickerton et al., 2012). When we compare the QED distribution of all antidiabetic compounds, we find that the compounds generated by transformation rules have a higher mean value (0.49) than the multi-target compounds obtained from the literature (0.46). The summary statistics of these plots can be found in the Supplementary material.

#### 3.3.2 Chemical multiverse of generated compounds

**FIGURE 5:**
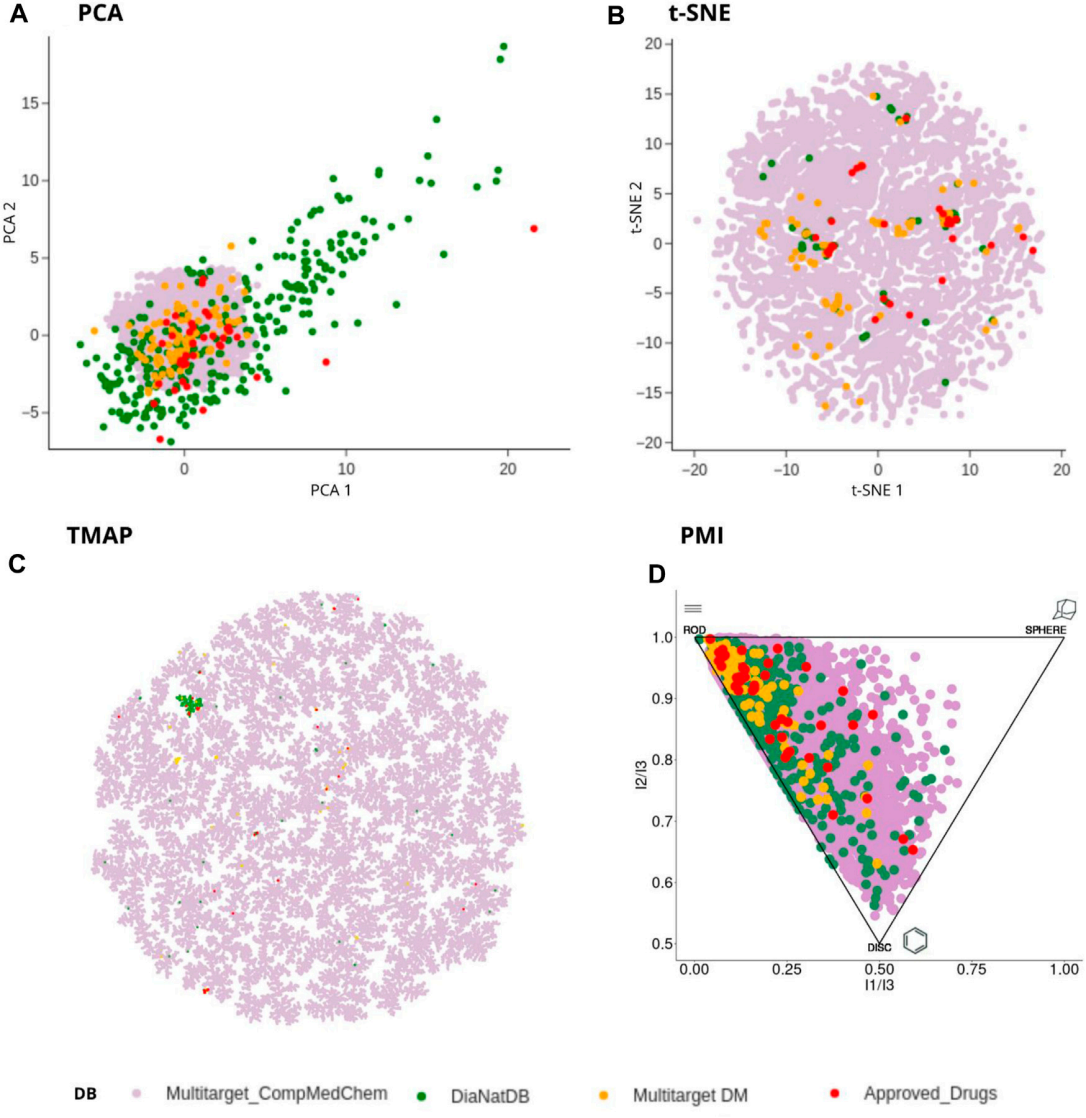
Visual representation of the chemical multiverse of antidiabetic compounds contained in approved drugs (red) DiaNat-DB (green), multi-target compounds (orange) and multi-target compounds generated by medicinal chemistry transformation rules (purple). **(A)** PCA of six physicochemical properties: MW, HBD, HBA, logP, TPSA and RB **(B)** t-SNE of six physicochemical properties (*vide supra*), **(C)** TMAP based on molecular ECFP4 fingerprint. **(D)** PMI space. Each corner on the triangular PMI plot indicates compounds with certain shape characteristics. The top left corner of the PMI represents compounds with rodlike shape, the top right corner represents compounds with spherical shape, and the bottom corner represents compounds with disc-like shape.

The concept of chemical multiverse (e.g., for the same compound datasets, different chemical spaces, each based on a different set of descriptors) was used to compare comprehensively the visual representation of the chemical space of the generated compounds with collections of reference compounds (Figure 5). The PCA of six physicochemical properties: MW, HBD, HBA, logP, TPSA and RB shows DiaNat-DB is the most diverse database in terms of physicochemical properties (Figure 5A). Using a non-linear method such as t-SNE and the same descriptors, we obtain a different visualization that allows us to visualize clusters or groups of data points and their relative proximities (5b). In terms of molecular fingerprints (ECFP4) (5c) and molecular shapes (5d), the library generated with transformation rules exhibited the largest structural and shape diversity, which is even larger than that of the DiaNat-DB database.

### 3.4 Filtering of compounds with relevance in pharmaceutical chemistry

To focus the library on attractive and synthetically feasible compounds, computational filters were applied as indicated in Section 2.6 of the Methods section. Table 4 summarizes the criteria used and the number of compounds remaining after applying the filters. As can be seen, the filter that considerably reduced the number of compounds was the QED value. The filtered compounds are characterized by not having chiral centers. If this is an important feature, you will need to consider it when applying the filters. Filtered compounds are listed in the Supplementary material.

**TABLE 4:** Filters applied and the number of compounds remaining for virtual screening.

Filters	Compound 3	Compound 4
Initial (ComMedChem rules)	72,349	33,661
Curated^a^	52,185	32,593
SAScore ≤ 6	52,185	32,552
Rsynth > 0.5	43,625	29,166
QED > 0.67	2,276	3,226
RDKit Molecule Catalog Filter = 0	1,543	451

^a^ For diversity studies, the set of curated structures was used.

### 3.5 Structure-based filtering

**FIGURE 6:**
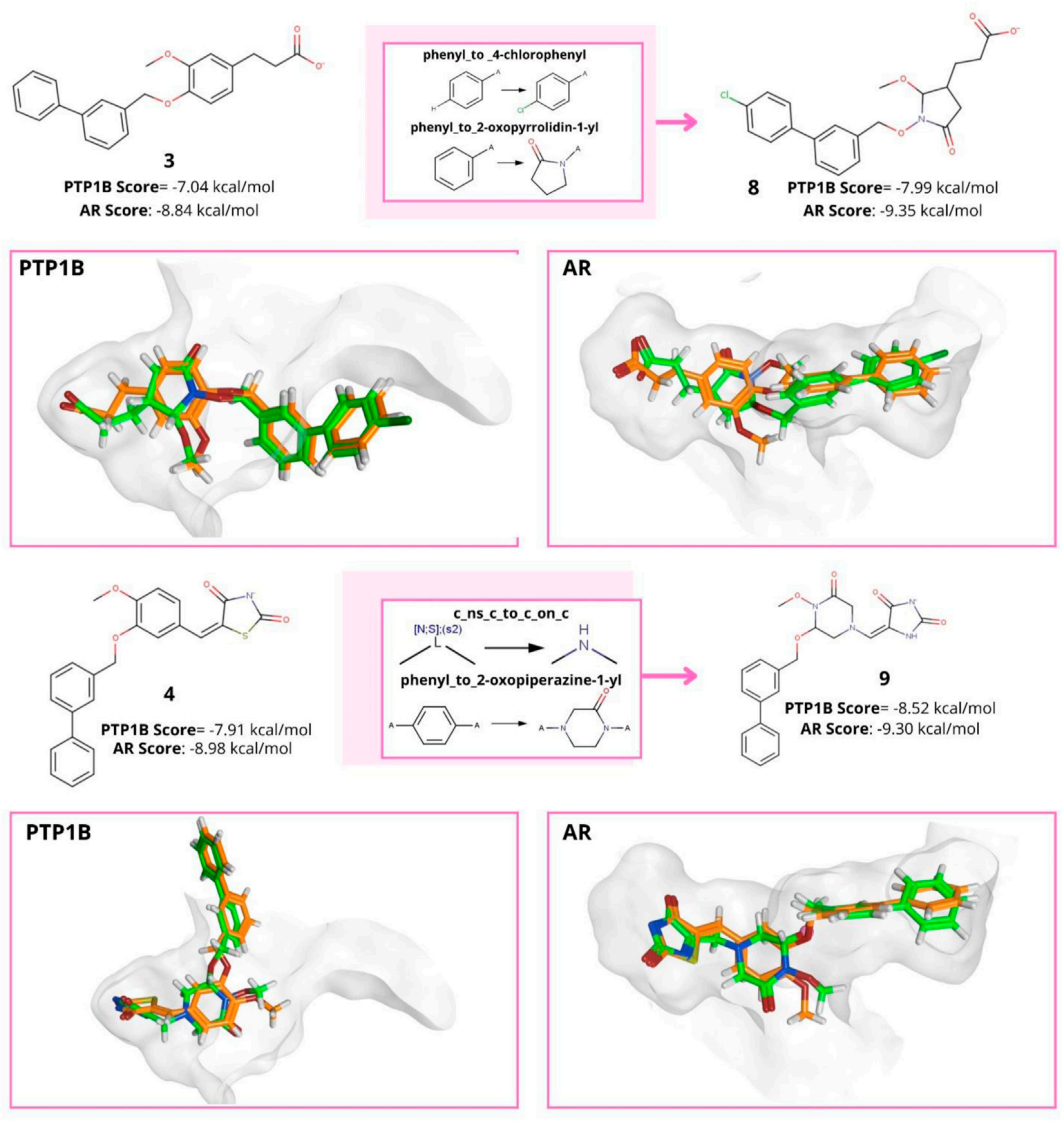
Examples of compounds selected from the multi-target virtual library, transformation rules used, and the calculated docking scores. 3D docking models for the proposed compounds (green) that overlap with the reference compounds (orange) for PTP1B and AR are included.

The compounds selected from the filtering described in Section 3.6 were subjected to a docking-based virtual screening with PTP1B and AR. The docking protocols are described in Section 2.6. The docking scores calculated with MOE for compounds **3** (PTP1B score: −7.04 kcal/mol and AR score = −8.84 kcal/mol) and **4** (PTP1B score: −7.91 kcal/mol and AR score = −8.98 kcal/mol) were used as cut-offs to select potential multi-target compounds. It was also checked whether the docking of these compounds reproduced the interactions previously reported in molecular dynamics studies (Domínguez-Mendoza et al., 2021). Supplementary Table S2 shows the score values and interaction plots of compounds **3** and **4** with PTP1B and AR. The first virtual screening with PTP1B yielded 1,655 compounds with a lower score value than the reference compounds: 1,543 from compound **3** and 112 from compound **4**. The virtual screening hit compounds were docked with AR yielding 816 hit compounds: 792 were from compound **3** and 24 from compound **4**. Figure 6 shows examples of designed compounds that have the potential to be used in multi-target approaches. The figure shows the parent structure (**3** and **4**), the transformation rules and the compounds designed with their corresponding calculated docking scores with PTP1B and AR. We also include the 3D docking models for the proposed compounds, and their overlap with the reference compounds. The docking results for PTP1B and AR of the 816 compounds can be found in the Supplementary material.

### 3.6 Prediction of ADME-Tox properties

Table 5 shows the average and standard deviation for different ADME-Tox properties of approved DMT2 drugs, multi-target compounds with reported *in vivo* activity and the 816 compounds that showed the potential to interact with PTP1B and AR. The values described to make an empirical decision are taken directly from the ADMElab 2.0 documentation (https://admetmesh.scbdd.com/explanatthat overlapion/index). As can be seen, the subset of compounds selected from the multi-target library generated in this work exhibit optimal adsorption and distribution properties. It is also noteworthy that in terms of metabolism, ADMETlab 2.0 predicts that the compounds generated in this work have a lower probability of being inhibitors of various CYPs compared to approved drugs and multi-target compounds that have a moderate to high probability of being inhibitors of CYP2C9, CYP2C19, and CYP3A4. Knowing this information is important because the inhibition of some CYPs is associated with the risk of hypoglycemia. Knowing this information is important because the inhibition of some CYPs is associated with the risk of hypoglycemia. For example, CYP2C9 metabolizes nateglinide, repaglinida, rosiglitazone, and most sulfonylureas, such as glibenclamide, glimepiride and glipizide (Holstein et al., 2012). Other examples are pioglitazone and repaginate metabolized with CYP3A4. ADMETlab 2.0 also predicts the probability of being substrates of various CYPs. This data and other properties calculated with ADMETlab 2.0 for each compound can be found in the Supplementary material. It is important to mention that the prediction of inhibitory activity in ADMETlab 2.0 is based on a dataset containing information on inhibitory activity of compounds obtained by high-throughput quantitative screening with an *in vitro* bioluminescence assay (Veith et al., 2009). However, in the description of these data, it is mentioned that the inhibitory activity in the assay may be due to compounds acting as substrates and that some weakly binding substrates may be classified as “inactive,” so that the predictions may need further confirmation.

**TABLE 5:** Estimation of selected ADME-Tox properties of 816 compounds with ADMETlab 2.0^a^ ^,^ ^b^.

ADME-tox properties	Empirical decision	Approved drugs	Multi-target from literature	Multi-target CompMedChem^c^
% Lipinski	MW ≤ 500; logP≤5; Hacc≤10; Hdon≤5 < 2 violations: Accepted; ≥2 violations: Rejected	92.85%	83.51%	100%
LogD at pH = 7.4	Compounds in the range from 1 to 3 log mol/L will be considered proper	1.5807 ± 1.9413	3.4817 ± 1.1394	2.3117 ± 1.257
Caco-2 Permeability	> −5.15: excellent; otherwise: poor	−5.3635 ± 0.5697	−5.0198 ± 0.3816	−5.1287 ± 0.282
Volume Distribution (VD)	0.04–20: excellent; otherwise: poor	0.7733 ± 0.6735	0.6091 ± 0.4483	0.3805 ± 0.1896
CYP1A2-inhibitor	The output value is the probability of being inhibitor, within the range of 0–1	0.1543 ± 0.2344	0.4157 ± 0.2926	0.1384 ± 0.1618
CYP2C9 inhibitor		0.3849 ± 0.3621	0.7215 ± 0.2534	0.287 ± 0.2952
CYP2C19 inhibitor		0.2601 ± 0.3034	0.5659 ± 0.3208	0.2154 ± 0.2288
CYP2D6-inhibitor	0–0.3: excellent; 0.3–0.7: medium; 0.7–1: poor	0.1736 ± 0.2457	0.2659 ± 0.2953	0.1115 ± 0.1707
CYP3A4-inhibitor		0.3034 ± 0.3448	0.3790 ± 0.3219	0.0992 ± 0.1289
Clearance (CL)	>15: excellent	4.4716 ± 3.9932	4.0985 ± 3.3766	6.2131 ± 3.2198
	5–15: medium			
	<5: poor			
Half-life time (T_1/2_)	0–0.3: excellent; 0.3–0.7: medium; 0.7–1: poor	0.4080 ± 0.2759	0.3083 ± 0.2511	0.7309 ± 0.1447
hERG Blockers	0–0.3: excellent; 0.3–0.7: medium; 0.7–1.0: poor	0.2179 ± 0.2446	0.2705 ± 0.2587	0.1631 ± 0.1621
Human hepatotoxicity (H-HT)	0–0.3: excellent; 0.3–0.7: medium; 0.7–1.0: poor	0.5874 ± 0.3218	0.6796 ± 0.2888	0.6023 ± 0.2631
Drug - Induced liver Injury (DILI)	0–0.3: excellent; 0.3–0.7: medium; 0.7–1.0: poor	0.6480 ± 0.3965	0.9073 ± 0.195	0.7124 + 0.3434

^a^ Compounds that showed the potential to interact with PTP1B and AR, selected in Section 3.5.

^b^ The color coding represents if the criteria in the empirical decision column are met (green), partially met (yellow), and not (red). The color coding in the table is the same as the one used in ADMETlab 2.0.

^c^ Compounds that showed the potential to interact with PTP1B and AR, selected in Section 3.5

In terms of excretion, clearance (CL) and half-life (T_1/2_) are important pharmacokinetic parameters that allow defining a drug´s dosing frequency. In the case of ADMETlab 2.0, the half-life is not measured in units of time. The output value is the probability of falling into category 1 (T_1/2_ ≤ 3). That is, the greater the probability of falling into category 1, the more likely the substance is to be classified as “poor” because its T_1/2_ would be lower (T_1/2_ ≤ 3). For antidiabetic drugs, the average CL is 4.4716 mL/min/kg (poor) and the T_1/2_ is 0.4080 (medium). The discrepancy in predictions could be due to the difference in models or datasets. In the case of the generated multi-target compounds, they may not be optimal for reducing the frequency of administration. Finally, although the probability of compounds being hERG blockers is reduced, the likelihood of drug-induced liver injury (DILI) would remain a challenge to optimize.

## 4 Conclusion

Designing multi-target compounds is an attractive approach to develop therapeutic treatments for complex diseases such as T2DM and MetS. Herein, we collected from the literature and analyzed ninety-one multi-target compounds for which *in vivo* antidiabetic activity has been reported, with a total of twenty target combinations. Following an enumeration based on transformation rules, we expanded the relevant chemical space of two of these multi-target hit compounds. More than 450 transformation rules were applied, of which 280 are made openly available to the scientific community. We concluded that the compounds generated with transformation rules have similar physicochemical properties to antidiabetic drugs and multi-target compounds reported in literature. Of the 84,778 generated compounds with valid structures, 85% are predicted to be synthetically feasible. The enumerated compounds are also attractive considering structural and shape diversity.

To focus on attractive and synthetically feasible compounds to perform virtual screening, various drug-likeness and quality filters were applied, yielding a multi-target virtual library with 2037 compounds. After a docking-based virtual screening with PTP1B and AR, 816 multi-target compounds were selected. Compounds in this library have favorable ADME properties, making the library an attractive source of promising candidates for further research and development.

In line with open and democratization of science, the newly designed multi-target focused library is freely available as a valuable source of starting points for chemical synthesis, biological evaluation, or further computational analysis such as virtual screening or reference libraries in library design.

## Data Availability

The original contributions presented in the study are publicly available. This data can be found here: https://figshare.com/projects/Design_of_a_multi-target_focused_library_for_antidiabetic_targets_using_a_comprehensive_set_of_chemical_transformation_rules/175194.

## Abbreviations

ADME, absorption, distribution, metabolism, and excretion; AR, aldose reductase; DM, diabetes mellitus; FFA1, Free fatty acid receptor 1; GPR40, G-protein-coupled receptor 40; MetS, metabolic syndrome; MOE, Molecular Operating Environment; MW, molecular weight; PCA, principal component analysis; PMI, principal moments of inertia; PPARs, peroxisome proliferator-activated receptors; PTP1B, protein tyrosine phosphatase 1B; QED, quantitative estimate of drug-likeness; RB, rotatable bonds; T2DM, Type II diabetes mellitus; TMAP, tree map; TPSA, topological polar surface area; t-SNE, t-distributed stochastic neighbor embedding; SMILES, Simplified Molecular Input Line Entry Specification, SMIRKS: line notation for specifying reaction transforms; SUR, Sulfonylurea receptor.
