# Supplementary material for: Design of a multi-target focused library for antidiabetic targets using a comprehensive set of chemical transformation rules
Source: Front Pharmacol. 2023 Nov 2;14:1276444. doi: 10.3389/fphar.2023.1276444 (PMC10651762; doi:10.3389/fphar.2023.1276444)
Supplement: Supplementary file 1 [file Table1.pdf]

SUPPLEMENTARY MATERIAL

Design of a multi-target focused library for antidiabetic targets using a comprehensive set of chemical transformation rules

Fernanda I. Saldivar-González [1,*], Gabriel Navarrete-Vázquez [2], José L. Medina-Franco [1,*]

[1] DIFACQUIM research group, School of Chemistry, Department of Pharmacy, Universidad Nacional Autónoma de México, Avenida Universidad 3000, Mexico City 04510, Mexico

[2] Faculty of Pharmacy, Universidad Autónoma del Estado de Morelos, Cuernavaca, Morelos 62209, Mexico

* Corresponding authors: F.I. S-G. (fer.saldivarg@gmail.com) J.L.M.-F. (medinajl@unam.mx)

CONTENTS **PAGE**

**Table S1**	Summary statistics of physicochemical properties of pharmaceutical relevance.	S2
**Table S2**	Summary of structure-based filtering	S4
**Table S3**	Transformation rules retrieved from the literature.	S5

Table S1. Summary statistics of physicochemical properties of pharmaceutical relevance

Molecular Weight (MW)							
	Min.	1st Qu.	Median	Mean	3rd Qu.	Max.	sd
Approved drugs	129.20	315.90	413.70	562.10	461.90	4113.60	793.22
DiaNatDB	117.10	270.20	358.60	390.60	478.40	952.60	177.43
Multi-target	281.30	381.40	437.50	447.70	500.60	628.40	81.28
Multi-target ComMedChem	310.30	403.90	425.50	428.30	449.60	551.50	33.55

Topological polar surface area (TPSA)							
	Min.	1st Qu.	Median	Mean	3rd Qu.	Max.	sd
Approved drugs	12.03	77.39	94.78	172.57	113.15	1693.14	342.06
DiaNatDB	0.00	57.53	98.36	118.28	167.67	455.18	80.02
Multi-target	43.37	64.99	88.94	88.75	107.78	148.49	26.59
Multi-target ComMedChem	35.53	76.99	87.34	88.84	99.52	151.86	16.87

LogP							
	Min.	1st Qu.	Median	Mean	3rd Qu.	Max.	sd
Approved drugs	-24.0348	1.1621	2.0100	0.8274	3.1315	6.3136	5.1290
DiaNatDB	-7.5730	0.0917	2.1474	1.9854	3.5508	14.2011	2.9480
Multi-target	0.6980	3.5130	4.7420	4.5100	5.4120	7.7010	1.5426
Multi-target ComMedChem	-0.1359	3.5077	4.3383	4.3454	5.1616	9.1863	1.2536

Rotatable Bonds (RB)							
	Min.	1st Qu.	Median	Mean	3rd Qu.	Max.	sd
Approved drugs	0.00	4.00	5.50	11.50	7.00	149.00	27.08
DiaNatDB	0.00	1.00	3.00	3.90	6.00	15.00	3.07
Multi-target	1.00	6.00	7.00	7.40	9.00	12.00	2.30
Multi-target ComMedChem	3.00	6.00	8.00	7.39	8.00	11.00	0.98

Cont. **Table S1**. Summary statistics of physicochemical properties of pharmaceutical relevance

Hydrogen-bond donors (HBD)							
	Min.	1st Qu.	Median	Mean	3rd Qu.	Max.	sd
Approved drugs	1.000	1.000	2.000	5.262	4.000	56.000	11.599
DiaNatDB	0.000	1.000	3.000	3.857	6.000	16.000	3.130
Multi-target	0.000	1.000	1.000	1.593	2.000	4.000	0.966
Multi-target ComMedChem	0.000	1.000	1.000	1.549	2.000	6.000	0.863

Hydrogen-bond acceptors (HBA)							
	Min.	1st Qu.	Median	Mean	3rd Qu.	Max.	sd
Approved drugs	1.000	4.000	6.000	8.095	7.000	59.000	11.539
DiaNatDB	0.000	3.000	6.000	6.951	10.000	27.000	4.849
Multi-target	2.000	5.000	6.000	5.593	6.000	13.000	1.692
Multi-target ComMedChem	2.000	5.000	6.000	6.520	8.000	12.000	1.511

Quantitative estimate of drug-likeness (QED)							
	Min.	1st Qu.	Median	Mean	3rd Qu.	Max.	sd
Approved drugs	0.0080	0.4462	0.5680	0.5714	0.8007	0.9090	0.2457
DiaNatDB	0.03533	0.23600	0.40905	0.41081	0.57214	0.87405	0.2096
Multi-target	0.1343	0.3245	0.4450	0.4683	0.6358	0.8255	0.1772
Multi-target ComMedChem	0.1097	0.4146	0.4936	0.4923	0.5698	0.8597	0.1196

Synthetic accessibility score (SA)							
	Min.	1st Qu.	Median	Mean	3rd Qu.	Max.	sd
Approved drugs	1.786	2.681	3.418	3.729	3.846	9.961	1.707
DiaNatDB	1.097	2.895	4.007	3.988	4.824	7.546	1.314
Multi-target	1.661	2.372	2.589	2.703	2.901	4.687	0.575
Multi-target ComMedChem	1.737	3.064	3.615	3.631	4.084	6.342	0.724

Table S2. Summary of structure-based filtering.

<table>
<tr><td>Compound 3
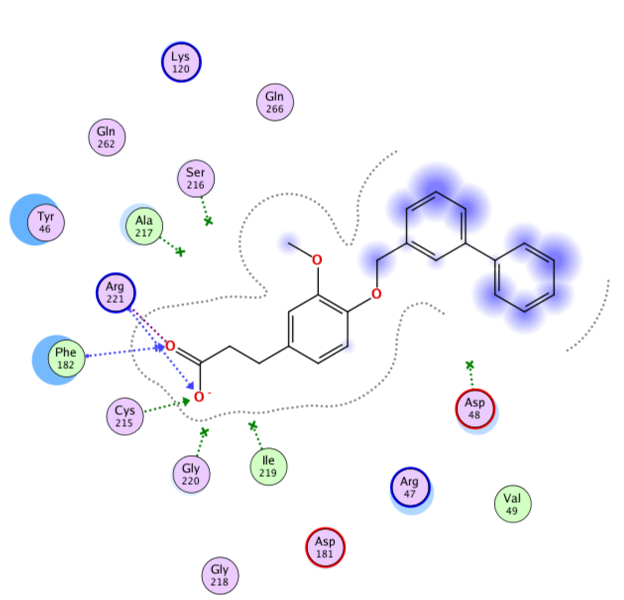
PTP1B docking score: -7.04 kcal/mol</td><td>Compound 4
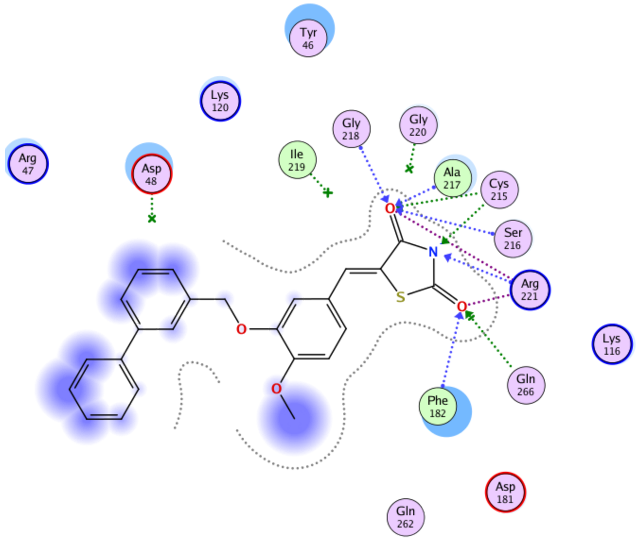
PTP1B docking score: -7.91 kcal/mol</td></tr>
<tr><td colspan="2">Virtual screening with PTP1B</td></tr>
<tr><td>1543</td><td>112</td></tr>
<tr><td colspan="2">Virtual screening with AR</td></tr>
<tr><td>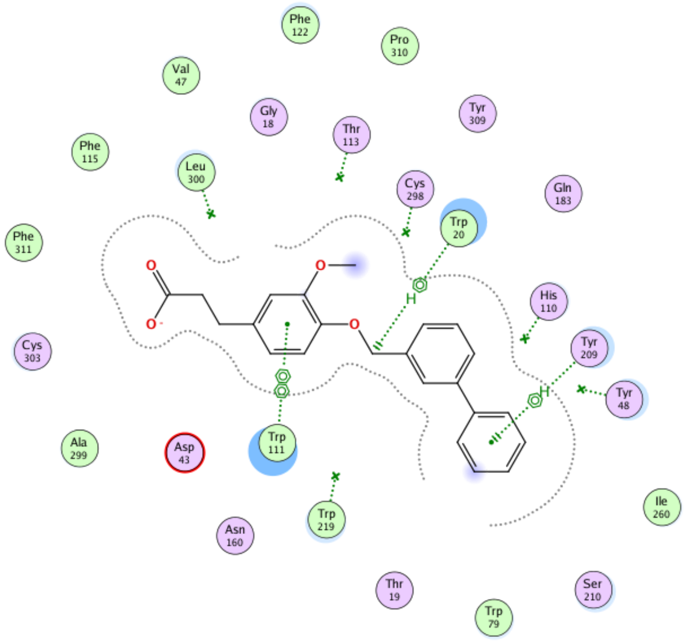
AR Score: -8.84 kcal/mol</td><td>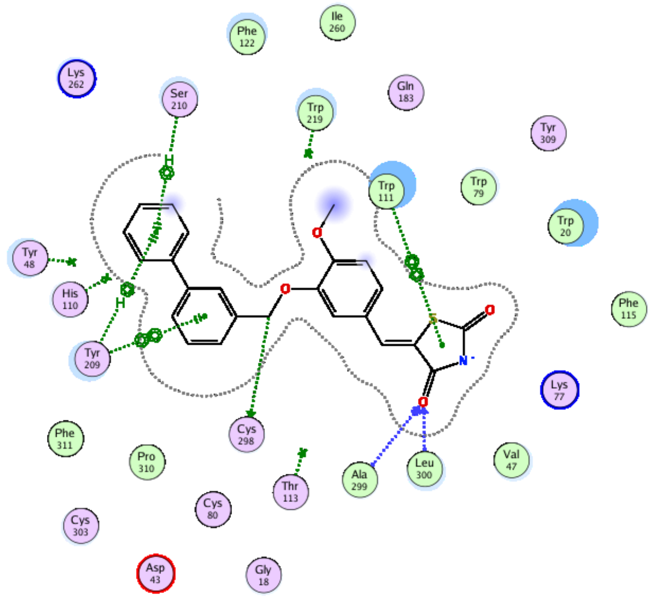
AR Score: -8.98 kcal/mol</td></tr>
<tr><td>792</td><td>24</td></tr>
</table>

Table S3. Transformation rules retrieved from the literature.

<table>
<tr><th>Group</th><th>Transformation</th><th>Type of transformation</th><th>SMIRKS</th><th>Note</th><th>Reference</th></tr>
<tr><td>Alcohol</td><td>Aliphatic_alcohol_to_CF2H
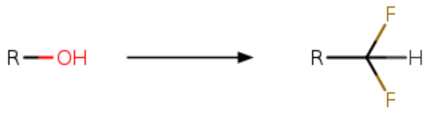</td><td>Functional group change</td><td>[#6;A;D2;H2:1][#8;D1;H1:2]>>[H][C:2]([#6;A:1])(F)F</td><td>-Bioisoster replacement
-Improved membrane permeability
-Enhancing specificity</td><td>(Erickson and McLoughlin, 1995; Xu et al., 2004)</td></tr>
<tr><td>Alcohol</td><td>Alcohol_to_sulfoximines
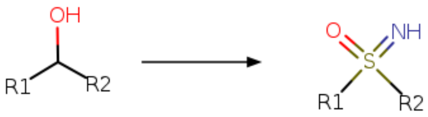</td><td>Functional group change</td><td>[#8;D1:4]-[#6;!R:2](-[*:1])-[C:3]>[#8]>[*:1][S:2]([C:3])(=[#7])=[O:4]</td><td>-Isosteric replacement</td><td>(Lu and Vince, 2007)</td></tr>
<tr><td>Alcohol</td><td>Alcohol_to_sulfone
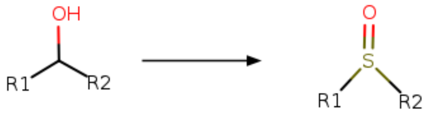</td><td>Functional group change</td><td>[#8;D1:4]-[#6;!R:2](-[*:1])-[*:3]>>[*:1][S:2]([*:3])=[O:4]</td><td>-Isosteric replacement</td><td>(Lu and Vince, 2007)</td></tr>
<tr><td>Alkyl</td><td>Metyl_to_cyclopropane
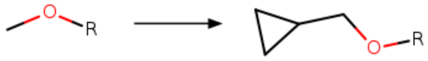</td><td>Atom addition</td><td>[#6;A;H3:1]-[#8;A;!R:2]-[#6;A:3]>>[#6;A:3]-[#8;A;!R:2]-[#6:1]-[#6]-1-[#6]-[#6]-1</td><td>-Metabolic stability (more resistant to CYP450 metabolic oxidation)</td><td>(Li, 2020)</td></tr>
<tr><td>Alkyl</td><td>CH2_to_O
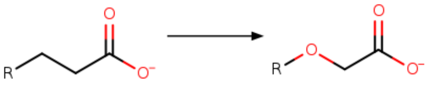</td><td>Atom substitution</td><td>[#6;A:6][#6;A:5][#6;A:4][#6;A:2]([#8;D1:1])=[O:3]>>[#6:6]-[#8:5]-[#6:4]-[#6:2](-[#8;D1-:1])=[O:3] |s:4:1|</td><td>-Metabolic stability (impede b-oxidation of aliphatic acid)</td><td>(Li, 2020)</td></tr>
<tr><td>Alkyl</td><td>t-butyl_to_trifluoromethyl_oxetane
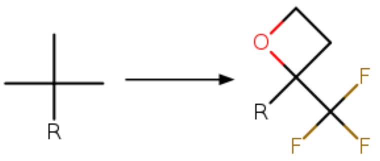</td><td>Functional group change</td><td>[#6:1]C([#6;AH3])([#6;AH3])[#6;AH3]>>[#6:1]C1([#6]-[#6]-[#8]1)C(F)(F)F</td><td>-Metabolic stability
-Decreased lipophilicity
-Improved LLE
-Bioisosteric replacement</td><td>(Mukherjee et al., 2017)</td></tr>
<tr><td>Alkyl</td><td>t-butyl_to_trifluoromethyl
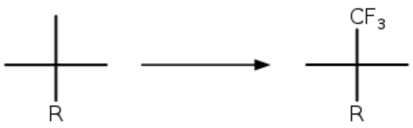</td><td>Atom substitution</td><td>[#6:1]C([#6;AH3])([#6;AH3])[#6;AH3]>>[#6:1]C([#6])([#6])C(F)(F)F</td><td>-Metabolic stability (more resistant to ω-oxidation)</td><td>(Furet et al., 2013)</td></tr>
<tr><td>Alkyl</td><td>t-butyll_to_trifluoromethyl_cyclopropane
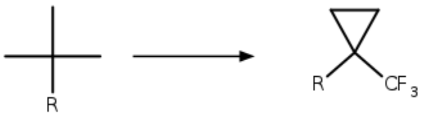</td><td>Functional group change</td><td>[#6:1]C([#6;AH3])([#6;AH3])[#6;AH3]>>[#6:1]C1([#6]-[#6]1)C(F)(F)F</td><td>Metabolic stability (more resistant to ω-oxidation)</td><td>(Barnes-Seeman et al., 2013)</td></tr>
</table>

Alkyl	Gem-dimethyl_to 3,3-diaryloxetane<br>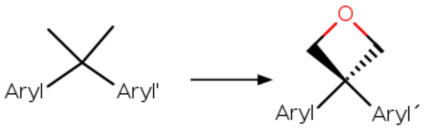	Functional group change	[#6;a:1][#6;A:2]([#6;a:3])([#6;H3])[#6;H3]>>[#6;a:3][#6;A@:2]1([#6;a:1])[#6]-[#8:4]-[#6]1	-Metabolic stability<br>-Reduce phototoxicity<br>-Improved solubility<br>-Improved permeability	(Dubois et al., 2021)
Diaryl	Diaryl_to_3,3-diaryloxetanes<br>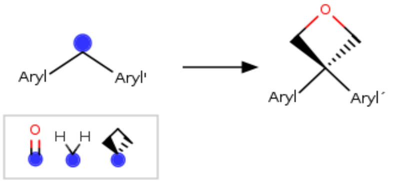	Functional group change	[#6;a:1][C;$([#6:2]=O),$([#6;A;@:2]1[#6]-[#6]-[#6]1),$([#6;H2])][#6;a:3]<br>>>[#6;a:3][#6;A@:2]1([#6;a:1])[#6]-[#8:4]-[#6]1	-Metabolic stability<br>-Reduce phototoxicity<br>-Improved solubility<br>-Improved permeability	(Dubois et al., 2021)
Amide	Amide_to_imidazole<br>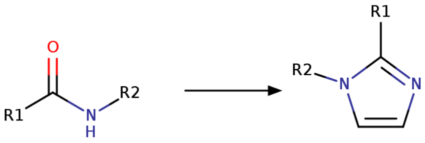	Ring addition/linker modification	[#6:4]-[#6;X3:2](=[O;X1:3])[#7;A;H1X3:1][*:5]>>[#6:4]-[#6;X3R1:2]1=[#7:3]-[#6]=[#6][#7;A;X3:1]1[*:5] |c:7,t:5|	-Bioisoster replacement<br>- Metabolic stability (generally resistant to cleavage mediated by proteases, oxidation and hydrolysis).	(Dundee et al., 1984)
Amide	Amide_to_imidazole2<br>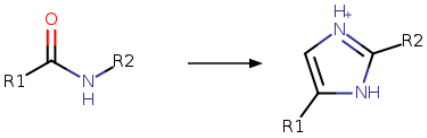	Ring addition/linker modification	[#6:3]-[#6;!R:2](=O)-[#7;!R:1]-[*:4]>>[#6:3]-[#6:2]-1=[#7]-[#6]=[#6](-[*:4])-[#7:1]-1<br><br>*Amide not in Aliphatic Ring	-Bioisoster replacement<br><br>-Increase bioavailability (rat)	(Kumari et al., 2020)
Amide	Amide_to_triazole1<br>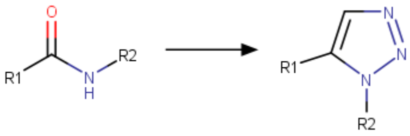	Ring addition/linker modification	[#6:4]-[#6;X3:2](=[O;X1:3])[#7;A;X3;H:1][*:5]>>[#6:4]-[#6;X3:2]1=[#6:3]-[#7]=[#7][#7;A;X3]1[*:5]	-Bioisoster replacement<br><br>-Metabolic stability	(Kumari et al., 2020)
Amide	Amide_to_triazole2<br>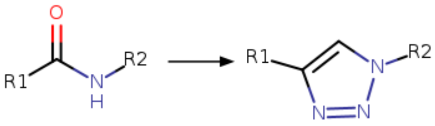	Ring addition/linker modification	[#6:4]-[#6;X3:2](=[O;X1:3])[#7;A;H1X3:1][*:5]>>[#6:4]-[#6;X3:2]1=[#6:3]-[#7]=[#7][#7;A;X3:1]1[*:5]<br>*Amide not in Aliphatic Ring	-Bioisoster replacement<br><br>-Metabolic stability	(Kumari et al., 2020)
Amide	Amide_to_thiazole<br>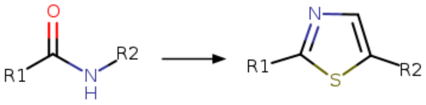	Ring addition / linker modification	[#6:3]-[#6;!R:2](=O)-[#7;!R:1]-[*:4]>>[#6:3]-[#6:2]-1=[#7]-[#6]=[#6](-[*:4])-[#16:1]-1<br><br>*Amide not in Aliphatic Ring	-Bioisoster replacement<br><br>-Metabolic stability	(Kumari et al., 2020)
Amide	Amide_to_1_2_4_oxadiazole<br>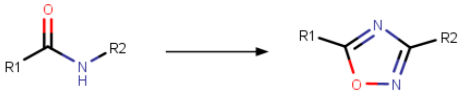	Ring addition / linker modification	[#6:1]-[#6;X3;!R:2](=[O;X1:3])[#7;A;H1X3;!R:4][*:5]>>[#6:1]-[#6:2]-1=[#7;A;X3:4][#6;X3](-[*:5])=[#7]-[#8:3]-1<br><br>*Amide not in Aliphatic Ring	- Bioisoster replacement<br><br>-Metabolic stability	(Benmansour et al., 2016; Kumari et al., 2020; Camci and Karali, 2023)

Amide	Amide_to_1_3_4_oxadiazole<br>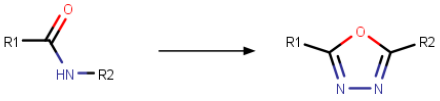	Ring addition / linker modification	[#6:1]-[#6;X3;!R:2](=[O;X1:3])[#7;A;H1X3;!R:4][*:5]>>[#6:1]-[#6:2]-1=[#7]-[#7:4]=[#6](-[*:5])-[#8:3]-1<br>*Amide not in Aliphatic Ring	-Bioisoster replacement<br>-Metabolic stability	(Benmansour et al., 2016; Kumari et al., 2020)
Amide	Amide_to__1_2_5_oxadiazole<br>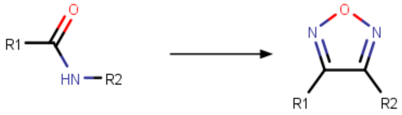	Ring addition /linker modification	[#6:1]-[#6;X3;!R:2](=[O;X1:3])[#7;A;H1X3;!R:4][*:5]>>[#6:1]-[#6:2]-1=[#7]-[#8:3]-[#7:4]=[#6]-1-[*:5]<br>*Amide not in Aliphatic Ring	-Bioisoster replacement<br>-Metabolic stability<br>- Increase oral bioavailability<br>-Improved potency and gut permeability.	(Nakajima et al., 2017; Kumari et al., 2020)
Amide	Amide_to_tetrazole<br>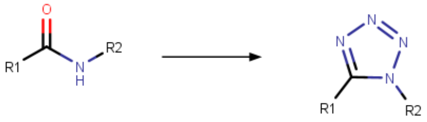	Ring addition /linker modification	[#6:4]-[#6;X3:2](=[O;X1:3])-[#7;H1:1]-[*:5]>>[#6:4]-[#6;X3:2]-1=[#7:3]-[#7]=[#7]-[#7:1]-1-[*:5]	-Bioisoster replacement<br>- Metabolic stability (generally resistant to cleavage mediated by proteases, oxidation and hydrolysis)	(Kumari et al., 2020)
Amide	Amide_to_tetrazole2<br>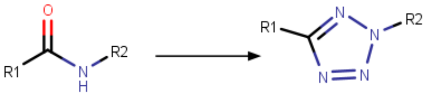	Ring addition /linker modification	[#6:4]-[#6;X3:2](=[O;X1:3])-[#7;X3:1]-[*:5]>>[#6:4]-[#6:3]-1=[#7:2]-[#7:1](-[*:5])-[#7]=[#7]-1	-Bioisosteric replacement	(Kumari et al., 2020)
Amide	Amide_to_E_olefin<br>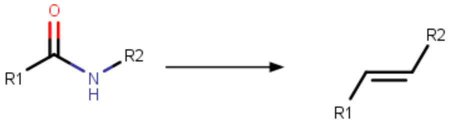	Linker modification	[#6:3]-[#6;!R:2](=O)-[#7;!R:1]-[*:4]>>[#6:3]\[#6:2]=[#6:1]\[*:4]<br>*Amide not in Aliphatic Ring	-Bioisosteric replacement<br>- Metabolic stability (generally resistant to cleavage mediated by proteases, oxidation and hydrolysis)<br>-Increase bioavailability	(Kim et al., 2014; Randolph et al., 2018; Kumari et al., 2020)
Amide	Amide_to_fluoroalkene<br>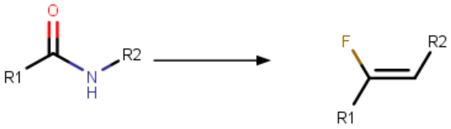	Linker modification	[#6:3]-[#6;!R:2](=O)-[#7:1]-[*:4]>>[#6:3]\[#6:2](F)=[#6:1]\[*:4]<br>*Amide not in Aliphatic Ring	-Bioisosteric replacement<br>-Metabolic stability<br>-Improved lipophilicity<br>-Increase in half-life in human plasma<br>* Reactive structure	(Chang et al., 2012; Altman et al., 2018; Meanwell, 2018; Kumari et al., 2020)
Amide	Amide_to_urea<br>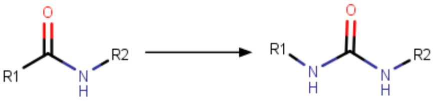	Linker modification	[#6:3]-[#6;!R:2](=O)-[#7H1;!R:1]-[*:4]>>[#6:3]-[#7]-[#6:2](=O)-[#7:1]-[*:4]<br>*Amide not in Aliphatic Ring	-Bioisosteric replacement<br>-Increase solubility	(Jagtap et al., 2017; Kumari et al., 2020)
Amide	Amide_to_carbamato<br>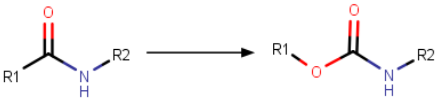	Linker modification	[#6:3]-[#6;!R:2](=O)-[#7H1;!R:1]-[*:4]>>[#6:3]-[#8]-[#6:2](=O)-[#7:1]-[*:4]<br>*Amide not in Aliphatic Ring	-Bioisosteric replacement<br>-Some degree of rigidity<br>-Improved permeability	(Fransson et al., 2014; Kumari et al., 2020)

Amide	Amide_to_ester<br>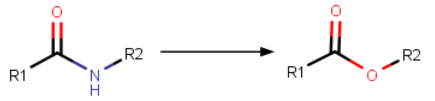	Linker modification	[#6:3]-[C;!R:2](=O)-[N:1]-[*:4]>>[#6:3]-[#6:2](=O)-[#8:1]-[*:4]<br><br>*Amide not in Aliphatic Ring	-Bioisosteric replacement<br><br>-Deleterious to metabolic stability	(Kumari et al., 2020)
Amide	Amide_to_thioamide<br>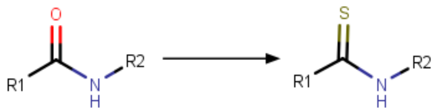	Linker modification	[#6:3]-[#6;A;!R:2](=O)-[#7;A:1]-[*:4]>>[#6:3]-[#6:2](=S)-[#7:1]-[*:4]<br><br>*Amide not in Aliphatic Ring	-Bioisosteric replacement<br><br>-Increased rotational constriction	(Kumari et al., 2020)
Amide	Amide_to_phosphonamidate<br>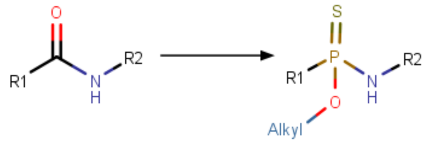	Linker modification	[#6:3]-[#7:1]-[#6;!R:2](-[#6:4])=[O:5]>>[#6:3]-[#7:1][P:2]([*:4])(=[O:5])[#8]-[#6]<br><br>*Alkyl= CH_3	-Bioisosteric replacement<br>-Increased water solubility and charge.<br>-Metabolically labile<br>-Reduced cell permeability	(Kumari et al., 2020)
Amide	Amide_to_sulfonamide<br>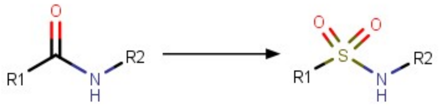	Linker modification	[#6:3]-[N:1][#6;A;!R:2]([*:4])=O>>[#6:3]-[#7:1][S:2]([*:4])(=O)=O	-Bioisosteric replacement<br><br>-Increased water solubility	(Kumari et al., 2020)
Amide	Amide_to_trifluoroethylamine<br>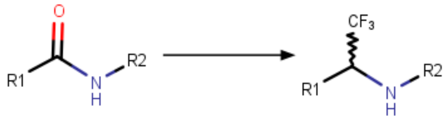	Linker modification	[#6:3]-[#7;A:1]-[#6;A;!R:2](-[*:4])=O>>[#6:3]-[#7:1]-[#6:2](-[*:4])C(F)(F)F	-Bioisosteric replacement<br><br>-Increased lipophilicity	(Black et al., 2005; Kumari et al., 2020)
Amide	Amide_to_difluoroethanamine<br>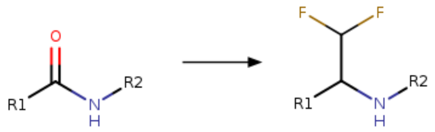	Linker modification	[#6:3][#7;A:1][#6;A;!R:2]([*:4])=O>>[#6:3]-[#7:1]-[#6:2](-[*:4])-[#6](F)F	-Bioisosteric replacement<br>-Increased the basicity of the nitrogen which positively impacted the logD	(Isabel et al., 2011; Meanwell, 2018)
Amide	Amide_to_amidine<br>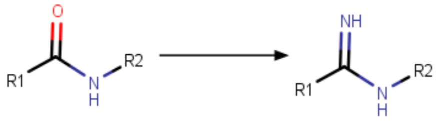	Linker modification	[#6:3][#7;A:1][#6;A;!R:2]([*:4])=O>>[#6:3]-[#7:1]-[#6:2](-[*:4])=[#7]	-Bioisosteric replacement<br>-Increased charged and likely reduction of BBB penetration.	(Kumari et al., 2020)
Amide	Amide_to_trifluoropropene<br>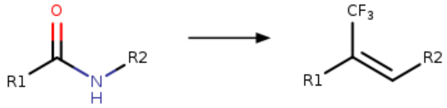	Linker modification	[#6:3][#7;A:1][#6;A;!R:2]([*:4])=O>>[#6:3]\[#6:1]=[#6:2](\[*:4])C(F)(F)F	-Isosteric replacement	(Meanwell, 2018)
Amide	Amide_to_methylamine<br>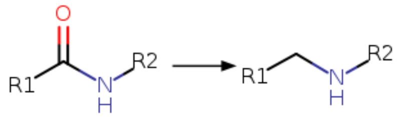	Linker modification	[#6:3][#7;A:1][#6;A;!R:2]([*:4])=O>>[#6:3]-[#7:1]-[#6:2]-[*:4]	-Biososteric replacement<br><br>-Free rotation around carbon and nitrogen.	(Evelyn et al., 2010; Kumari et al., 2020)

Amide	Aryl_amide_to_Amino-oxetane<br>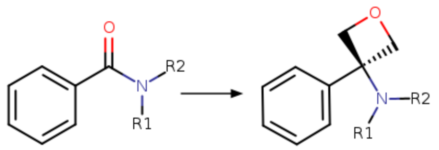	Linker modification	[c;X3:2][#6;A;X3;!R:3]([#7:4])=O>>[c:2][C:3]1([#7:4])[#6]-[#8]-[#6]1	-Isosteric replacement<br><br>-Increased sterics	(Rojas et al., 2022)
Amide	ArylAmide_to_3_acyl_indole<br>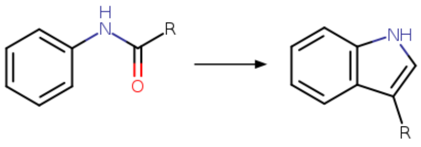	Ring Creation	[#6:5][#6;A;!R:4](=O)[#7H1:3]-[c;X3:2]:[c:1]>>[#6:5]-[#6]-1=[#6:4]-[#7H1:3]-[#6:2]=,:[#6:1]-1	-Biososteric replacement<br>-Increased sterics<br>-Increased lipophicity<br>-Increase half-life and %F	(Sheppard et al., 1994; Kumari et al., 2020)
Amine	Aryl_amine_to_amino_pyridine<br>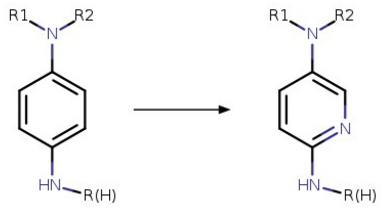	Ring addition	[#7;A;X3;!H1:7][c:1]1[c;D2:6][c;D2:5][c:4](-[#7;X3;H2,H1:8])[c;D2:3][c;D2:2]1>>[#7:8]-[#6:4]-1=[#6:3]-[#7:2]=[#6:1](-[#7:7])-[#6;D2:6]=[#6;D2:5]-1 |s:7:2|	-Metabolic stability (lower abundance of GSH conjugate)	(Zhang et al., 2020)
Amine	Aryl_amine_to_pyridazine<br>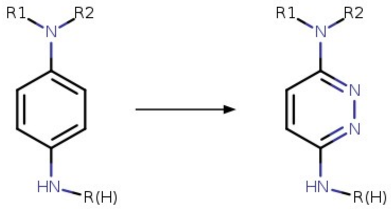	Ring modification	[#7;A;X3;!H1:7][c:1]1[c;D2:6][c;D2:5][c:4](-[#7;X3;H2,H1:8])[c;D2:3][c;D2:2]1>>[#7:8]-[#6:4]-1=[#7:3]-[#7:2]=[#6:1](-[#7:7])-[#6;D2:6]=[#6;D2:5]-1 |s:7:2,6:2|	-Metabolic stability (lower abundance of GSH conjugate)	(Zhang et al., 2020)
Aniline	Aniline_to_aminoBCP<br>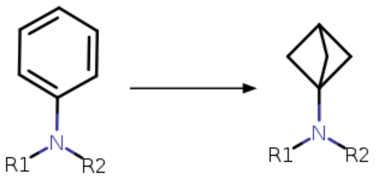	Ring substitution	[*:3]-[#7:2](-[*:1])-[c:4]1[c;D2][c;D2][c;D2][c;D2][c;D2]1>>[*:3]-[#7:2](-[*:1])[C:4]12[#6]-[#6](-[#6]1)-[#6]2	-Metabolic stability (more resistant to reactive metabolite formation and CYP-inhibition)<br><br>-Isosteric replacement	(Sodano et al., 2020)
Aniline	Aniline_to_aminoBCO<br>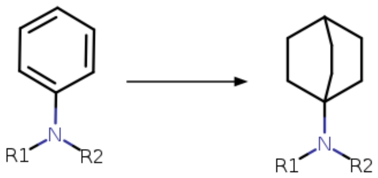	Ring substitution	[*:1]-[#7:2](-[*:3])-[c:4]1[c;D2][c;D2][c;D2][c;D2][c;D2]1>>[*:3]-[#7:2](-[*:1])[C:4]12[#6]-[#6]-[#6](-[#6]-[#6]1)-[#6]-[#6]2	-Metabolic stability (more resistant to reactive metabolite formation and CYP-inhibition)<br><br>-Isosteric replacement	(Sodano et al., 2020)
Aniline	Aniline_to_aminoCUB<br>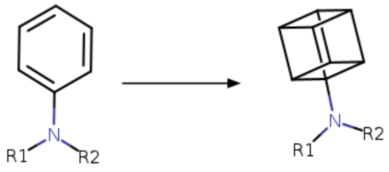	Ring substitution	[*:1]-[#7:2](-[*:3])-[c:4]1[c;D2][c;D2][c;D2][c;D2][c;D2]1>>[*:3]-[#7:2](-[*:1])[C:4]12[#6]-3-[#6]-4-[#6]-5-[#6]-3-[#6]1-[#6]-5-[#6]2-4	-Metabolic stability (more resistant to reactive metabolite formation and CYP-inhibition)<br><br>-Isosteric replacement	(Sodano et al., 2020)

<table>
<tr><td>Aniline</td><td>Aniline_to_aminoNB
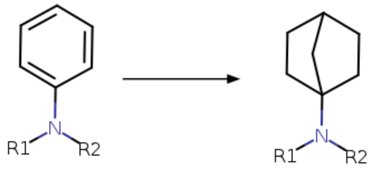</td><td>Ring substitution</td><td>[*:3]-[#7:2](-[*:1])-[c:4]1[c;D2][c;D2][c;D2][c;D2][c;D2]1>>[*:1]-[#7:2](-[*:3])[C:4]12[#6]-[#6]-[#6](-[#6]-[#6]1)-[#6]2</td><td>-Metabolic stability (more resistant to reactive metabolite formation and CYP-inhibition)

-Isosteric replacement</td><td>(Sodano et al., 2020)</td></tr>
<tr><td>Carbamate</td><td>Carbamate _to_oxazole
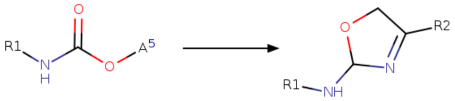</td><td>Linker modification</td><td>[*:1]-[#7;A:2]-[#6;A;!R:3](=O)-[#8:4]-[*:5]>>[*:1]-[#7:2]-[#6:3]-1-[#8:4]-[#6]-[#6](-[*:5])=[#7]-1 |c:11|</td><td>-Improved potency

-Improved margin of safety</td><td>(Peng et al., 2023)</td></tr>
<tr><td>Carboxylate</td><td>Carboxylate_to_tetrazole
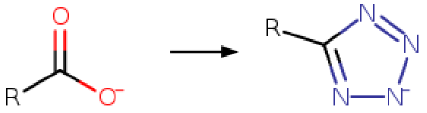</td><td>Ring addition</td><td>[#8;D1:4]-[#6:2](-[*:1])=[O:3]>>[*:1]-[*:2]-1=[#7:4]-[#7-]-[#7]=[#7:3]-1 |s:0:1|</td><td>-Biososteric replacement
-Similar acidic pKa
-More stability and enhanced permeability</td><td>(Navarrete-Vázquez et al., 2013; Mittal and Awasthi, 2019; Li, 2020; Bredael et al., 2022)</td></tr>
<tr><td>Carboxylate</td><td>Carboxylate_to_oxo_oxadiazole
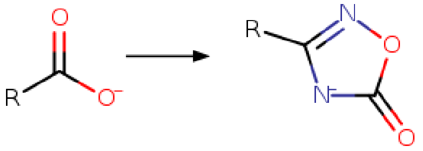</td><td>Ring addition</td><td>[#8;D1][#6;A;!R:2]([*:1])=O>>[*:1]-[#6:2]-1=[#7]-[#8]-[#6](=O)-[#7-]-1 |s:0:1|</td><td>-Biososteric replacement

-Metabolic stability</td><td>(Bredael et al., 2022)</td></tr>
<tr><td>Carboxylate</td><td>Carboxylate_to_oxo_thiadiazole
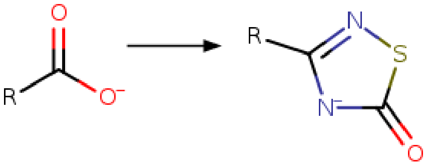</td><td>Ring addition</td><td>[#8;D1][#6;A;!R:2]([*:1])=O>>[*:1]-[#6:2]-1=[#7]-[#16]-[#6](=O)-[#7-]-1 |s:0:1|</td><td>-Biososteric replacement

-Metabolic stability</td><td>(Bredael et al., 2022)</td></tr>
<tr><td>Carboxylate</td><td>Carboxylate_to_thiazolidine-2,4-dione
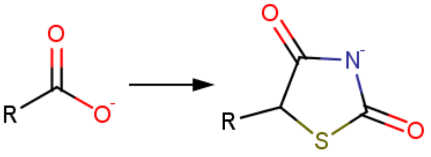</td><td>Ring addition</td><td>[#8;D1][#6;A;!R:2]([*:1])=O>>[*:1]-[#6:2]-1-[#16]-[#6](=O)-[#7-]-[#6]-1=O |s:0:1|</td><td>-Biososteric replacement

-Metabolic stability

- Similar acidic pKa</td><td>(Hidalgo-Figueroa et al., 2013)</td></tr>
<tr><td>Carboxylate</td><td>Carboxylate_to_oxazolidine-2,4-dione
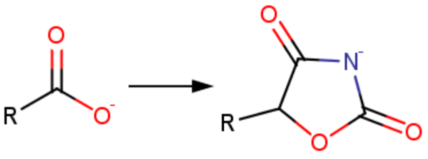</td><td>Ring addition</td><td>[#8;D1][#6;A;!R:2]([*:1])=O>>[*:1]-[#6:2]-1-[#8]-[#6](=O)-[#7-]-[#6]-1=O |s:0:1|</td><td>-Biososteric replacement

-Metabolic stability

- Similar acidic pKa</td><td>(Hidalgo-Figueroa et al., 2013)</td></tr>
</table>

Carboxylate	Carboxylate_to_thioxo_oxadiazole<br>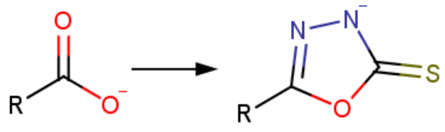	Ring addition	[#8;D1][#6;A;!R:2]([*:1])=O>>[*:1]-[#6:2]-1=[#7]-[#7-]-[#6](=S)-[#8]-1 |s:0:1|	-Biososteric replacement<br>-Metabolic stability	(Bredael et al., 2022)
Carboxylate	Carboxylate_to_thioxo_oxadiazole2<br>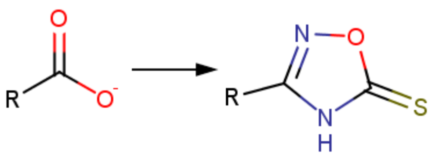	Ring addition	[#6:1][#6;A;!R:2]([#8;D1])=O>>[#6:1]-[#6:2]-1=[#7]-[#8]-[#6](=S)-[#7]-1 |s:2:1|	-Biososteric replacement<br>-Metabolic stability	(Bredael et al., 2022)
Carboxylate	Carboxylate_to_triazole<br>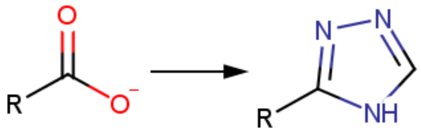	Ring addition	[#8;D1]-[#6:2](-[*:1])=O>>[*:1]-[#6:2]-1=[#7]-[#7]=[#6]-[#7]-1	-Biososteric replacement<br>-Metabolic stability	(Bredael et al., 2022)
Carboxylate	Carboxylate_to_imidazole<br>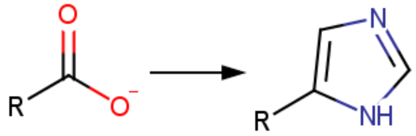	Ring addition	[#8;D1]-[#6;A;!R:2](-[*:1])=O>>[*:1]-[#6:2]-1=[#6]-[#7]=[#6]-[#7]-1|s:0:1|	-Biososteric replacement<br>-Metabolic stability	(Bredael et al., 2022)
Carboxylate	Carboxylate_to_4hydroxy_oxadiazole<br>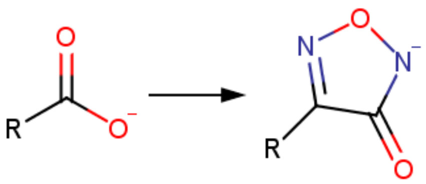	Ring addition	[#8;D1]-[#6:2](-[*:1])=O>>[*:1]-[#6:2]-1=[#7]-[#8]-[#7-]-[#6]-1=O	-Biososteric replacement	(Bredael et al., 2022)
Carboxylate	Carboxylate_to_hidroxipyrazol<br>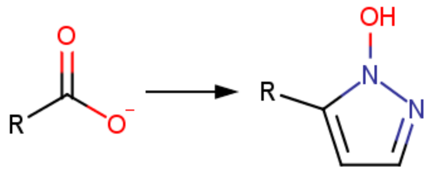	Ring addition	[#8;D1]-[#6:2](-[*:1])=O>>[#8]-[#7]-1-[#7]=[#6]-[#6]=[#6:2]-1-[*:1]	-Biososteric replacement	(Bredael et al., 2022)
Carboxylate	Carboxylate_to_cyclopetane-1_3-dione<br>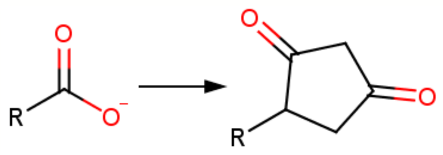	Ring addition	[#6:1][#6;A;!R:2]([#8;D1])=O>>[#6:1]-[#6:2]-1-[#6]-[#6](=O)-[#6]-[#6]-1=O |s:2:1|	-Biososteric replacement	(Ballatore et al., 2011)
Carboxylate	Carboxylate_to_cyclopentane_1_2_dione<br>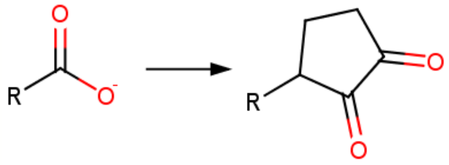	Ring addition	[#8;D1]-[#6:2](-[*:1])=O>>[*:1]-[#6:2]-1-[#6]-[#6]-[#6](=O)-[#6]-1=O	-Biososteric replacement	(Ballatore et al., 2014)

Carboxylate	Carboxylate_to_cyclopentane_1_2_dione2 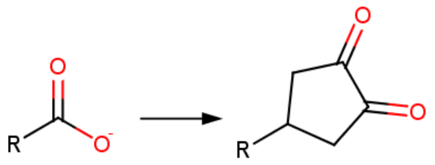	Ring addition	[#8;D1]-[#6:2](-[*:1])=O>>[*:1]-[#6:2]-1-[#6]-[#6](=O)-[#6](=O)-[#6]-1	-Biososteric replacement	(Bredael et al., 2022)
Carboxylate	Carboxylate_to_tetronic_acid 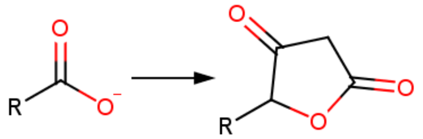	Ring addition	[#6:1][#6;A;!R:2]([#8;D1])=O>>[#6:1]-[#6:2]-1-[#8]-[#6](=O)-[#6]-[#6]-1=O |s:2:1|	-Isosteric replacement	(Ballatore et al., 2013)
Carboxylate	Carboxylate_to_tetramic_acid 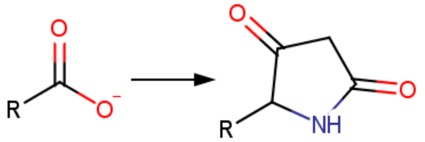	Ring addition	[#6:1][#6;A;!R:2]([#8;D1])=O>>[#6:1]-[#6:2]-1-[#7]-[#6](=O)-[#6]-[#6]-1=O |s:2:1|	-Isosteric replacement	(Ballatore et al., 2013)
Carboxylate	Carboxylate_to_hydantoin 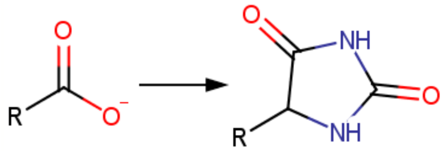	Ring addition	[#8;D1][#6;A;!R:2]([*:1])=O>>[*:1]-[#6:2]-1-[#7]-[#6](=O)-[#7]-[#6]-1=O |s:0:1|	- Isosteric replacement<br><br>-Similar acidic pKa	(Ahmed et al., 2011)
Carboxylate	Carboxylate_to_barbituric acid 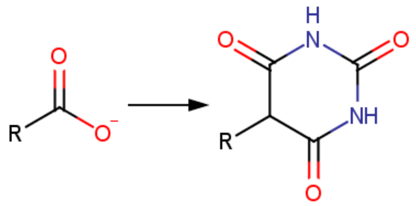	Ring addition	[#8;D1][#6;A;!R:2]([*:1])=O>>[*:1]-[#6:2]-1-[#6](=O)-[#7]-[#6](=O)-[#7]-[#6]-1=O |s:0:1|	-Biososteric replacement<br><br>-Similar acidic pKa	(Sundriyal et al., 2008; Hidalgo-Figueroa et al., 2013)
Carboxylate	Carboxylate_to_squaric_acid 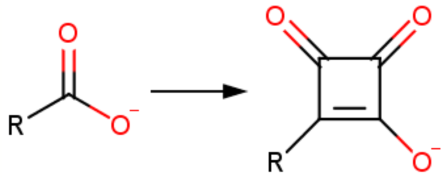	Ring addition	[#6:1][#6;A;!R:2]([#8;D1])=O>>[#6:1]-[#6:2]-1=[#6](-[#8-])-[#6](=O)-[#6]-1=O |s:2:1|	- Isosteric replacement<br><br>* Reactive structure associated with toxicity, a lack of selectivity, and thus promiscuity towards several targets	(Agnew-Francis and Williams, 2020; Bredael et al., 2022)
Carboxylate	Carboxylate_to_hydroxamic_acid 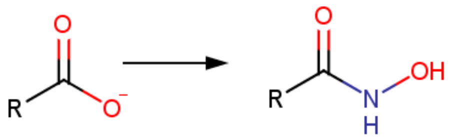	Functional group change	[#8;D1][#6;A;!R:2]([#6:1])=O>>[#8;H1]-[#7]-[#6:2](-[*:1])=O |s:0:1|	--Biososteric replacement<br><br>-Decrease metabolic stability (can undergo relatively rapid hydrolysis)	(Barrett et al., 2008; Ballatore et al., 2013)

<table>
<tr><td></td><td></td><td></td><td></td><td></td><td></td></tr>
<tr><td>Carboxylate</td><td>Carboxylate_to_trifluoromethylketones
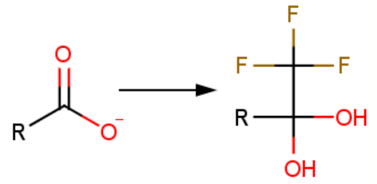</td><td>Functional group change</td><td>[#6:1]-[#6:2](-[#8;D1])=O>>[#8][C:2]([#8])([*:1])C(F)(F)F</td><td>-Biososteric replacement

-Increase the lipophilicity</td><td>(Ballatore et al., 2013; Bredael et al., 2022)</td></tr>
<tr><td>Carboxylate</td><td>Carboxylate_to_sulfonamide
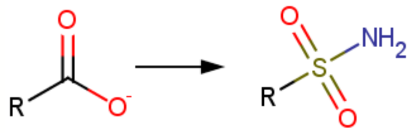</td><td>Functional group change</td><td>[#6:1]-[#6:2](-[#8;D1:4])=[O:3]>>[#7:4][S:2]([*:1])(=O)=[*:3]</td><td>-Biososteric replacement</td><td>(Bredael et al., 2022)</td></tr>
<tr><td>Carboxylate</td><td>Carboxylate_to_sulfonic_acid
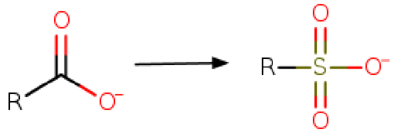</td><td>Functional group change</td><td>[#6:1][#6;A;!R:2]([#8;D1:3])=O>>[#6:1][S:2]([#8-:3])(=O)=O |s:2:1|</td><td>-Biososteric replacement</td><td>(Ballatore et al., 2013; Bredael et al., 2022)</td></tr>
<tr><td>Carboxylate</td><td>Carboxylate_to_sulfonamide2
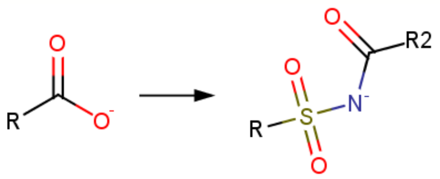</td><td>Functional group change</td><td>[#6:1][#6;A;!R:2]([#8;D1])=O>>[#6:1][S:2](=O)(=O)[#7-]-[#6](=O)-c1ccccc1 |s:2:1|

*R2 point of diversity
R2= Phenyl</td><td>-Biososteric replacement</td><td>(Bredael et al., 2022)</td></tr>
<tr><td>Carboxylate</td><td>Carboxylate_to_sulfonamide3
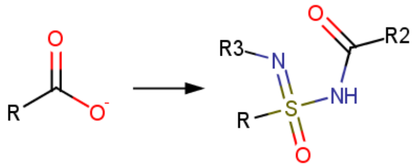</td><td>Functional group change</td><td>[#8;D1]-[#6:2](-[*:1])=O>>[*:1][S:2](=[#7])(=O)[#7]-[#6](=O)-c1ccccc1

*R2 and R3 points of diversity
R2= Phenyl; R3=H</td><td>-Biososteric replacement</td><td>(Bredael et al., 2022)</td></tr>
<tr><td>Carboxylate</td><td>Carboxylate_to_acylsulfonamide
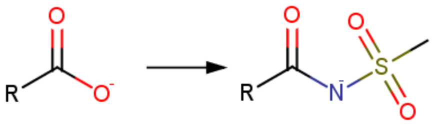</td><td>Functional group change.</td><td>[#8;D1:4]-[#6:2](-[*:1])=[O:3]>>[#6]S(=O)(=O)[#7-:4]-[*:2](-[*:1])=[*:3] |s:0:1|</td><td>-Biososteric replacement

- Similar acidic pKa</td><td>(Winters et al., 2008)</td></tr>
<tr><td>Carboxylate</td><td>Carboxylate_to_sulfonylurea
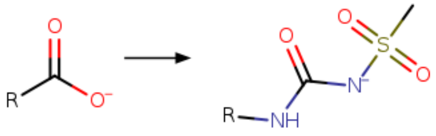</td><td>Functional group change.</td><td>[#6:1][#6;A;!R:2]([#8;D1])=O>>[#6:1]-[#7:2]-[#6](=O)-[#7-]S([#6])(=O)=O |s:2:1|</td><td>-Biososteric replacement</td><td>(Winters et al., 2008)</td></tr>
</table>

<table>
<tr><th></th><th></th><th></th><th></th><th></th><th></th></tr>
<tr><td>Carboxylate</td><td>Carboxylate_to_phosphonic_acid
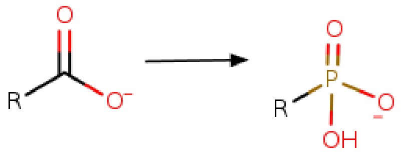</td><td>Functional group change.</td><td>[#6:1][#6;A;!R:2]([#8;D1])=[O:3]>>[#6:1][P:2]([#8])([#8-])=[O:3] |s:2:1|</td><td>-Isosteric replacement</td><td>(Froestl et al., 1995; Ballatore et al., 2013)</td></tr>
<tr><td>Carboxylate</td><td>Carboxylate_to_tetrazolone
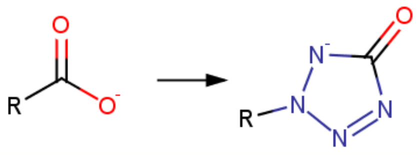</td><td>Ring addition</td><td>[#8;D1][#6;A;!R:2]([#6:1])=O>>[*:1]-[#7:2]-1-[#7]-[#6](=O)-[#7]=[#7]-1 |c:8,s:0:1|</td><td>-Biososteric replacement</td><td>(Bredael et al., 2022)</td></tr>
<tr><td>Carboxylate</td><td>Carboxylate_to_cyclic_sulfonimidamide
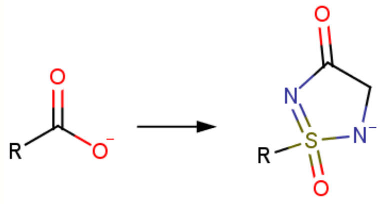</td><td>Ring addition</td><td>[#6:1][#6;A;!R:2]([#8;D1])=O>>[*:1][S:2]1(=O)=[#7]-[#6](=O)-[#6]-[#7-]1</td><td>-Biososteric replacement</td><td>(Bredael et al., 2022)</td></tr>
<tr><td>Carboxylate</td><td>Carboxylate_to_4_hydroxy_1_2-3_triazole
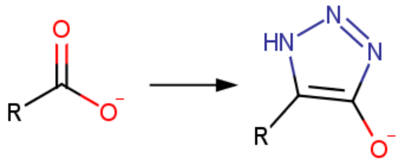</td><td>Ring addition</td><td>[#8;D1:3][#6;A;!R:2]([#6:1])=O>>[#8-:3]-[#6]-1=[#6:2](-[*:1])-[#7]-[#7]=[#7]-1 |s:0:1|</td><td>-Biososteric replacement</td><td>(Pippione et al., 2015; Bredael et al., 2022)</td></tr>
<tr><td>Carboxylate</td><td>Carboxylate_to_oxetan_3_ol
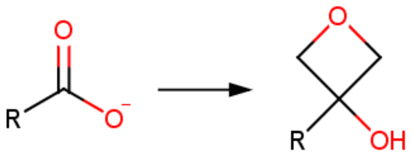</td><td>Ring addition</td><td>[#8;D1]-[#6;A;!R:2](-[#6:1])=O>>[#8;H][C:2]1([*:1])[#6]-[#8]-[#6]1</td><td>-Biososteric replacement

-Increased lipophilicity and permeability</td><td>(Lassalas et al., 2017; Bredael et al., 2022)</td></tr>
<tr><td>Carboxylate</td><td>Carboxylate_to_thietan_3_ol
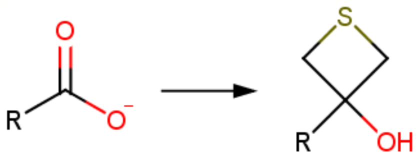</td><td>Ring addition</td><td>[#8;D1]-[#6:2](-[*:1])=O>>[#8;H][C:2]1([*:1])[#6]-[#16]-[#6]1</td><td>-Biososteric replacement

-Increased lipophilicity and permeability</td><td>(Lassalas et al., 2017; Bredael et al., 2022)</td></tr>
<tr><td>Carboxylate</td><td>Carboxylate_to_thietan_1_oxide_3_ol
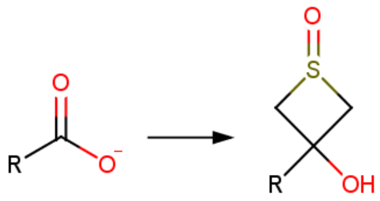</td><td>Ring addition</td><td>[#8;D1]-[#6:2](-[*:1])=O>>[#8;H][C:2]1([*:1])[#6]S(=O)[#6]1</td><td>-Biososteric replacement

-Increased lipophilicity and permeability</td><td>(Lassalas et al., 2017; Bredael et al., 2022)</td></tr>
</table>

<table>
<tr><td>Carboxylate</td><td>Carboxylate_to_thietan_1_1_dioxide_3_ol
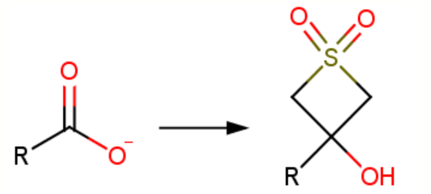</td><td>Ring addition</td><td>[#8;D1]-[#6:2](-[*:1])=O>>[#8H1][C:2]1([*:1])[#6]S(=O)(=O)[#6]1</td><td>-Biososteric replacement

-Increased lipophilicity and permeability</td><td>(Lassalas et al., 2017; Bredael et al., 2022)</td></tr>
<tr><td>Carboxylate</td><td>Carboxylate_to_2,6-difluorophenol
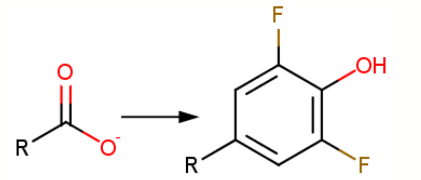</td><td>Ring addition</td><td>[#8;D1][#6;A;!R:2]([#6:1])=O>>[#8]-[#6]-1=[#6](F)-[#6]=[#6:2](-[*:1])-[#6]=[#6]-1F |s:0:1|</td><td>-Biososteric replacement

-Similar acidic pKa</td><td>(Chebib et al., 1999; Nicolaou et al., 2004)</td></tr>
<tr><td>Nitro</td><td>ArylNitro_to_benzofurazan
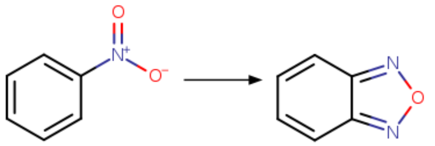</td><td>Ring addition</td><td>[#8-]-[#7+](=[O])-[c;X3:2]:[c:1]>>[#6:1]=,:1=[#7]-[#8]-[#7]=[#6:2]=,:1</td><td>-Metabolic stability</td><td>(Swain, n.d.)</td></tr>
<tr><td>Nitro</td><td>ArylNitro_to_phthalide
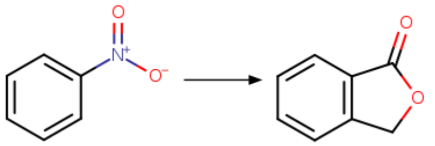</td><td>Ring addition</td><td>[#8-]-[#7+](=O)-[c;X3:2]:[c:1]>>O=[#6]-1-[#6:2]=,:[#6:1]-[#6]-[#8]-1</td><td>-Metabolic stability</td><td>(Swain, n.d.)</td></tr>
<tr><td>Nitro</td><td>ArylNitro_to_benzonitrile
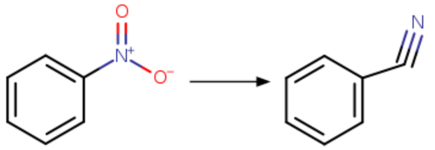</td><td>Functional group change</td><td>[#8-]-[#7+:3](=O)-[c;X3:2]:[c:1]>>[c:1]:[c;X3:2]C#[N:3]</td><td>-Metabolic stability</td><td>(Swain, n.d.)</td></tr>
<tr><td>Nitro</td><td>Aliphatic_Nitro_to_Trifluoromethyl
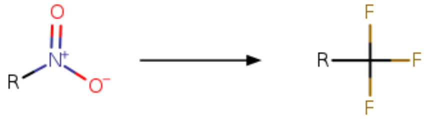</td><td>Functional group change</td><td>[#8-]-[#7+:2]([*;A:1])=O>>F[C:2](F)(F)[*;A:1]</td><td>-Bioisosteric replacement
-Metabolic stability</td><td>(Tseng et al., 2019)</td></tr>
<tr><td>Naftalen</td><td>a_sub_naphthalene_benzazaborinines
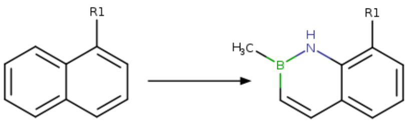</td><td>Ring modification</td><td>[*:1]-[c;D3:2]1[c;D2][c;D2][c;D2][c;D3]2[c;x2D2][c;x2D2][c;D2:5][c;D2:4][c;D3:3]12>>[#6]-[#5:5]-1-[#7:4]-[c;D3:3]2[c;D3:2](-[*:1])[c;D2][c;D2][c;D2][c;D3]2-[#6]=[#6]-1 |rb:6:2,7:2,s:1:3,8:2,9:2|</td><td>-Metabolic stability
-Bioisosteric replacement
-Improved bioavailability
-Improved brain penetration</td><td>(Rombouts et al., 2015)</td></tr>
<tr><td>Naftalen</td><td>a_sub_naphthalene_benzazaborinines2
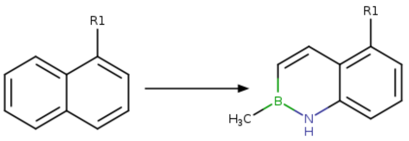</td><td>Ring modification</td><td>[*:1]-[c;D3:2]1[c;D2][c;D2][c;D2][c;D3]2[c;x2D2][c;x2D2][c;D2:5][c;D2:4][c;D3:3]12>>[#6]-[#5:5]-1-[#7:4]-[c;D3:3]2[c;D3:2](-[*:1])[c;D2][c;D2][c;D2][c;D3]2-[#6]=[#6]-1 |rb:6:2,7:2,s:1:3,6:2,7:2|</td><td>Metabolic stability
-Bioisosteric replacement
-Improve bioavailability
-Improve brain penetration</td><td>(Rombouts et al., 2015)</td></tr>
</table>

Naftalen	B_sub_naphthalene_benzazaborinines<br>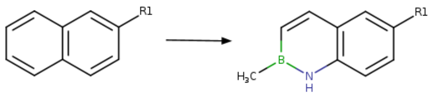	Ring modification	[*:8]-[c;D3:1]1[c;D2][c;D2][c;D3]2[c;x2D2:7][c;x2D2:6][c;D2:5][c;D2:4][c;D3:3]2[c;D2:2]1>>[#6]-[#5:6]-1-[#7:7]-[c;D3]2[c;D2][c;D2][c;D3:1](-[*:8])[c;D3:2][c;D3:3]2-[#6:4]=[#6:5]-1<br>|rb:5:2,6:2,s:5:2,6:2,1:3|	-Metabolic stability	(Rombouts et al., 2015)
Phenol	Phenol_to_indole<br>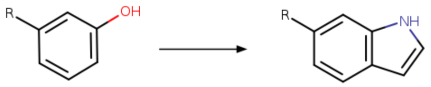	Ring addition	[#8;D1;H1:8]-[c;x2D3:4]1[c;x2D2:5][c;x2D2:6][c;D2:1][c;x2D3:2](-[*:7])[c;x2D2:3]1>>[*:7]-[c:2]1[c:1][c;x2D2:6][c:5]2-[#6]=[#6]-[#7:8]-[c:4]2[c;x2D2:3]1<br>|rb:1:2,2:2,3:2,5:2,7:2,11:2,17:2,s:1:3,2:2,3:2,4:2,5:3,7:2|	-Metabolic stability<br>-Isosteric replacement	(Di Grandi et al., 2009)
Phenol	Phenol_to_indazole<br>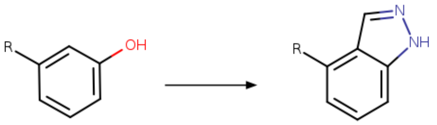	Ring addition	[#8;D1;H1:8]-[c;x2D3:4]1[c;x2D2:5][c;x2D2:6][c;D2:1][c;x2D3:2](-[*:7])[c;x2D2:3]1>>[*:7]-[c:2]1[c:1][c;x2D2:6][c:5][c:4]2-[#7:8]-[#7]=[#6]-[c:3]12<br>|rb:1:2,2:2,3:2,5:2,7:2,11:2,s:1:3,2:2,3:2,4:2,5:3,7:2|	-Metabolic stability<br><br>-Isosteric replacement	(Di Grandi et al., 2009)
Phenol	phenol_to_benzotriazole<br>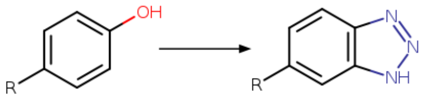	Ring addition	[#8;D1;H1:8]-[c;x2D3:4]1[c;x2D2:3][c;x2D2:2][c;x2D3:1](-[*:7])[c;x2D2:6][c;x2D2:5]1>>[*:7]-[c;x2D3:1]1[c;x2D2:2][c;x2D2:3][c:4]2-[#7:8]=[#7]-[#7]-[c:5]2[c;x2D2:6]1<br>|rb:1:2,2:2,3:2,4:2,6:2,7:2,9:2,10:2,11:2,17:2,s:1:3,2:2,3:2,4:3,6:2,7:2,9:3|	-Metabolic stability<br><br>-Isosteric replacement	(Wright et al., 2000)
Phenol	Phenol_to_indolone<br>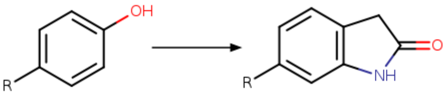	Ring addition	[#8;D1;H1:8]-[c;x2D3:4]1[c;x2D2:3][c;x2D2:2][c;x2D3:1](-[*:7])[c;x2D2:6][c;x2D2:5]1>>[*:7]-[c;x2D3:1]1[c;x2D2:2][c;x2D2:3][c:4]2-[#6:8]-[#6](=O)-[#7]-[c:5]2[c;x2D2:6]1<br>|rb:1:2,2:2,3:2,4:2,6:2,7:2,9:2,10:2,11:2,18:2,s:1:3,2:2,3:2,4:3,6:2,7:2,9:3|	-Metabolic stability<br><br>-Bioisosteric replacement	(Wright et al., 2000)
Phenol	Phenol_to_benzimidazolone<br>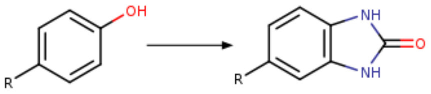	Ring addition	[#8;D1;H1:8]-[c;x2D3:4]1[c;x2D2:3][c;x2D2:2][c;x2D3:1](-[*:7])[c;x2D2:6][c;x2D2:5]1>>[*:7]-[c;x2D3:1]1[c;x2D2:2][c;x2D2:3][c:4]2-[#7:8]-[#6](=O)-[#7]-[c:5]2[c;x2D2:6]1<br>|rb:1:2,2:2,3:2,4:2,6:2,7:2,9:2,10:2,11:2,18:2,s:1:3,2:2,3:2,4:3,6:2,7:2,9:3|	-Metabolic stability<br>-Bioisosteric replacement	(Wright et al., 2000)
Phenol	Phenol_to_benzoxazolone<br>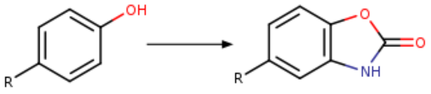	Ring addition	[#8;D1:H1:8]-[c;x2D3:4]1[c;x2D2:3][c;x2D2:2][c;x2D3:1](-[*:7])[c;x2D2:6][c;x2D2:5]1>>[*:7]-[c;x2D3:1]1[c;x2D2:2][c;x2D2:3][c:4]2-[#8:8]-[#6](=O)-[#7]-[c:5]2[c;x2D2:6]1<br>|rb:1:2,2:2,3:2,4:2,6:2,7:2,9:2,10:2,11:2,18:2,s:1:3,2:2,3:2,4:3,6:2,7:2,9:3|	-Bioisosteric replacement	(Wright et al., 2000)
Catechol	Catechol_to_aminothiazole<br>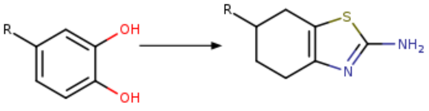	Ring addition	[#8;D1;H1:8]-[c;x2D3:4]1[c;x2D2:5][c;x2D2:6][c;x2D3:1](-[*:7])[c;x2D2:2][c;x2D3:3]1-[#8;D1;H1:9]>>[#7]-[#6]-1=[#7:8]-[#6:4]-2=[#6:3](-[#6:2]-[#6:1](-[*:7])-[#6:6]-[#6:5]-2)-[#16:9]-1<br>|rb:1:2,2:2,3:2,4:2,6:2,7:2,s:1:3,2:2,3:2,4:3,6:2,7:3|	-Isosteric replacement	(Hübner et al., 2000)

<table>
<tr><th></th><th></th><th></th><th></th><th></th><th></th></tr>
<tr><td>Catechol</td><td>Catechol_to_ethynylcyclohexene
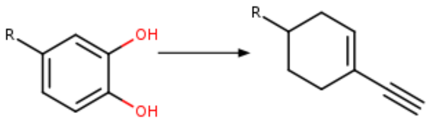
</td><td>Ring modification + Functional group change</td><td>[#8;D1;H1:8]-[c;x2D3:4]1[c;x2D2:5][c;x2D2:6][c;x2D3:1](-[*:7])[c;x2D2:2][c;x2D3:3]1-[#8;D1;H1]>>[*:7]-[#6:1]-1-[#6:6]-[#6:5]-[#6:4](=[#6:3]-[#6:2]-1)[C:8]#C |rb:1:2,2:2,3:2,4:2,6:2,7:2,s:1:3,2:2,3:2,4:3,6:2,7:3|</td><td>-Isosteric replacement</td><td>(Hübner et al., 2000)</td></tr>
<tr><td>Phenyl</td><td>Phenyl_to _4-fluorophenyl
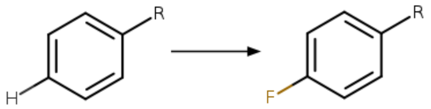

t</td><td>Atom addition</td><td>[*:6]-[c;x2D3:1]1[c;x2D2:2][c;x2D2:3][c;x2D2:4][c;x2D2][c;x2D2:5]1>>F[c;x2:4]1[c;x2D2][c;x2D2:5][c;x2D3:1](-[*:6])[c;x2D2:2][c;x2D2:3]1 |rb:1:2,2:2,3:2,4:2,5:2,6:2,8:2,9:2,10:2,11:2,13:2,14:2,s:1:3,2:2,3:2,4:2,5:2,6:2,11:3|</td><td>-Bioisosteric replacement
-Block metabolic site</td><td>(Navarrete-Vázquez et al., 2006)</td></tr>
<tr><td>Phenyl</td><td>Phenyl_to _4-chlorophenyl
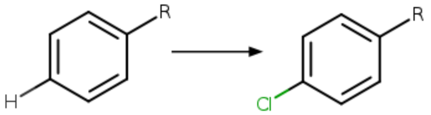

t</td><td>Atom addition</td><td>[*:6]-[c;x2D3:1]1[c;x2D2:2][c;x2D2:3][c;x2D2:4][c;x2D2][c;x2D2:5]1>>Cl[c;x2:4]1[c;x2D2][c;x2D2:5][c;x2D3:1](-[*:6])[c;x2D2:2][c;x2D2:3]1 |rb:1:2,2:2,3:2,4:2,5:2,6:2,8:2,9:2,10:2,11:2,13:2,14:2,s:1:3,2:2,3:2,4:2,5:2,6:2,11:3|</td><td>-Bioisosteric replacement
-Block metabolic site</td><td>(Navarrete-Vázquez et al., 2006)</td></tr>
<tr><td>Phenyl</td><td>Phenyl_to _4-cyanophenyl
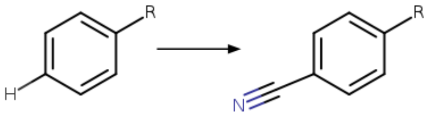

t</td><td>Atom addition</td><td>[*:6]-[c;x2D3:1]1[c;x2D2:2][c;x2D2:3][c;x2D2:4][c;x2D2][c;x2D2:5]1>>[*:6]-[c;x2D3:1]1[c;x2D2:5][c;x2D2][c;x2:4]([c;x2D2:3][c;x2D2:2]1)C#N |rb:1:2,2:2,3:2,4:2,5:2,6:2,8:2,9:2,10:2,11:2,12:2,13:2,s:1:3,2:2,3:2,4:2,5:2,6:2,8:3|</td><td>-Bioisosteric replacement
-Block metabolic site</td><td>(Navarrete-Vázquez et al., 2006)</td></tr>
<tr><td>Phenyl</td><td>Phenyl_to _4-trifluoromethylphenyl
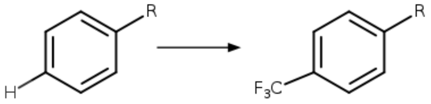

t</td><td>Atom addition</td><td>[*:6]-[c;x2D3:1]1[c;x2D2:2][c;x2D2:3][c;x2D2:4][c;x2D2][c;x2D2:5]1>>FC(F)(F)[c;x2:4]1[c;x2D2][c;x2D2:5][c;x2D3:1](-[*:6])[c;x2D2:2][c;x2D2:3]1 |rb:1:2,2:2,3:2,4:2,5:2,6:2,11:2,12:2,13:2,14:2,16:2,17:2,s:1:3,2:2,3:2,4:2,5:2,6:2,14:3|</td><td>-Bioisosteric replacement
-Block metabolic site</td><td>(Navarrete-Vázquez et al., 2006)</td></tr>
<tr><td>Phenyl</td><td>Phenyl_to _deuteromethoxyphenyl
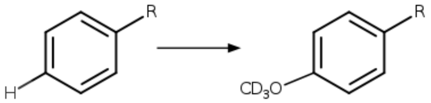

t</td><td>Atom addition</td><td>[*:6]-[c;x2D3:1]1[c;x2D2:2][c;x2D2:3][c;x2D2:4][c;x2D2][c;x2D2:5]1>>[2H]C([2H])([2H])[#8]-[c;x2:4]1[c;x2D2][c;x2D2:5][c;x2D3:1](-[*:6])[c;x2D2:2][c;x2D2:3]1 |rb:1:2,2:2,3:2,4:2,5:2,6:2,12:2,13:2,14:2,15:2,17:2,18:2,s:1:3,2:2,3:2,4:2,5:2,6:2,15:3|</td><td>-Bioisosteric replacement
-Block metabolic site</td><td>(Pirali et al., 2019)</td></tr>
<tr><td>Phenyl</td><td>Phenyl_to _pyridine
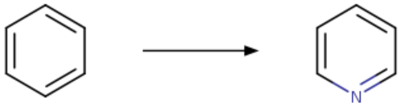

t, o, m, p</td><td>Ring modification</td><td>[c;x2:4]1[c;x2:3][c;x2:2][c;D2:1][c;x2:6][c;x2:5]1>>[c;x2:4]1[c;x2:5][c;x2:6][n:1][c;x2:2][c;x2:3]1 |rb:0:2,1:2,2:2,4:2,5:2,6:2,7:2,8:2,10:2,11:2,s:3:2|</td><td>-Isosteric replacement</td><td>(Subbaiah and Meanwell, 2021)</td></tr>
<tr><td>Phenyl</td><td>Phenyl_to _pyrimidine
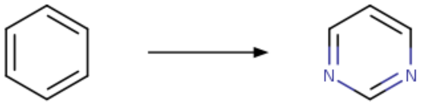

t, o, m, p</td><td>Ring modification</td><td>[c;x2:4]1[c;x2:3][c;D2:2][c;x2:1][c;D2:6][c;x2:5]1>>[c;x2:4]1[c;x2:5][n:6][c;x2:1][n:2][c;x2:3]1 |rb:0:2,1:2,3:2,5:2,6:2,7:2,9:2,11:2,s:2:2,4:2|</td><td>-Isosteric replacement</td><td>(Subbaiah and Meanwell, 2021)</td></tr>
</table>

<table>
<tr><td>Phenyl</td><td>Phenyl_to _pyrazine
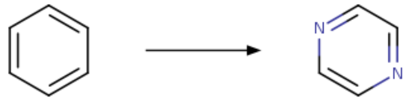
t, o, m, p</td><td>Ring modification</td><td>[c;x2:4]1[c;D2:3][c;x2:2][c;x2:1][c;D2:6][c;x2:5]1>>[c:1]1[c:2][n:3][c:4][c:5][n:6]1 |rb:0:2,2:2,3:2,5:2,s:1:2,4:2|</td><td>-Isosteric replacement</td><td>(Subbaiah and Meanwell, 2021)</td></tr>
<tr><td>Phenyl</td><td>Phenyl_to _pyridazine
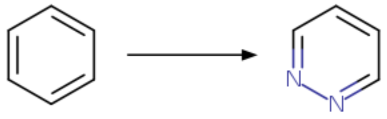
t, o, m, p</td><td>Ring modification</td><td>[c;x2:4]1[c;x2:3][c;x2:2][c;x2:1][c;D2:6][c;D2:5]1>>[c:2]1[c:3][c:4][n:5][n:6][c:1]1 |rb:0:2,2:2,3:2,,s:4:2,5:2|</td><td>-Isosteric replacement</td><td>(Subbaiah and Meanwell, 2021)</td></tr>
<tr><td>Phenyl</td><td>Phenyl_to _1_2_4_triazine
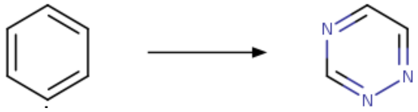
t, o, m, p</td><td>Ring modification</td><td>[c;D2:4]1[c;D2:3][c;x2:2][c;x2:1][c;D2:6][c;x2:5]1>>[c:1]1[c:2][n:3][n:4][c:5][n:6]1 |rb:2:2,3:2,5:2,s:0:2,1:2,4:2|</td><td>-Isosteric replacement</td><td>(Subbaiah and Meanwell, 2021)</td></tr>
<tr><td>Phenyl</td><td>Phenyl_to_tetrazine
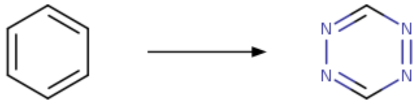
t, p</td><td>Ring modification</td><td>[c;x2:4]1[c;D2:5][c;D2:6][c;x2:1][c;D2:2][c;D2:3]1>>[c:4]1[n:5][n:6][c;x2:1][n:2][n:3]1 |rb:0:2,3:2,9:2,s:1:2,2:2,4:2,5:2|</td><td>-Isosteric replacement</td><td>(Subbaiah and Meanwell, 2021)</td></tr>
<tr><td>Phenyl</td><td>Phenyl_to_2pyridinone
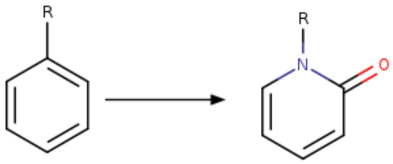
t, o, m, p</td><td>Ring modification</td><td>[*:7]-[c;x2D3:1]1[c;x2:6][c;x2:5][c;x2:4][c;x2:3][c;x2D2:2]1>>[*:7]-[#7:1]-1-[#6:6]=[#6:5]-[#6:4]=[#6:3]-[#6;X3x2:2]-1=O |c:9,11,rb:1:2,2:2,3:2,4:2,5:2,6:2,13:2,s:1:3|</td><td>-Improved potency
-Reduce off-target
-Metabolic stability
-Enhanced solubility
-Reduced lipophilicity</td><td>(Subbaiah and Meanwell, 2021)</td></tr>
<tr><td>Phenyl</td><td>Phenyl_to_2pyridinone2
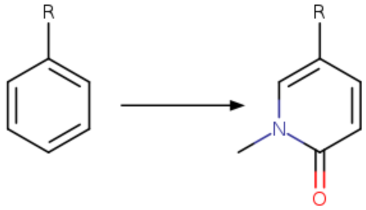
o, m</td><td>Ring modification</td><td>[*:7]-[c;x2D3:1]1[c;x2:2][c;D2:3][c;D2:4][c;x2:5][c;x2:6]1>>[#6]-[#7:3]-1-[#6:2]=[#6:1](-[*:7])-[#6:6]=[#6:5]-[#6:4]-1=O |c:12,t:9,rb:1:2,2:2,5:2,6:2,s:1:3,3:2,4:2|</td><td>-Enhanced solubility

-Reduced lipophilicity</td><td>(Ripa et al., 2018; Zhang and Pike, 2021)</td></tr>
<tr><td>Phenyl</td><td>Phenyl_to_4pyridinone
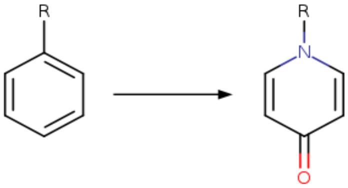
t, o, m</td><td>Ring modification</td><td>[*:7]-[c;x2D3:1]1[c;x2:6][c;x2:5][c;x2D2:4][c;x2:3][c;x2:2]1>>[*:7]-[#7:1]-1-[#6:2]=[#6:3]-[#6;x2:4](=O)-[#6:5]=[#6:6]-1 |c:9,13,rb:1:2,4:2,11:2,s:1:3,4:2 |</td><td>-Enhanced solubility
-Reduce off-target
-Reduced lipophilicity</td><td>(Tamura et al., 2012; Subbaiah and Meanwell, 2021; Zhang and Pike, 2021)</td></tr>
</table>

<table>
<tr><td>Phenyl</td><td>Phenyl_to_azaborinine
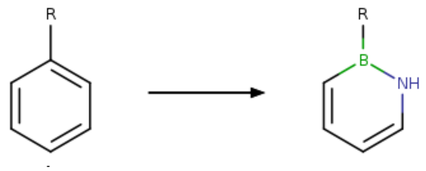
t, o, m, p</td><td>Ring modification</td><td>[*:7]-[c;D3:1]1[c;D2:2][c;x2:3][c;x2:4][c;x2:5][c;x2:6]1>>[*:7]-[#5:1]-1-[#7H1:2]-[#6:3]=[#6:4]-[#6:5]=[#6:6]-1 |c:10,12,rb:3:2,4:2,5:2,6:2,s:1:3|</td><td>-Enhanced solubility
-Improve bioactivity
-Improve in vivo oral availability</td><td>(Zhao et al., 2017)</td></tr>
<tr><td>Phenyl</td><td>Phenyl_to_pyrrole
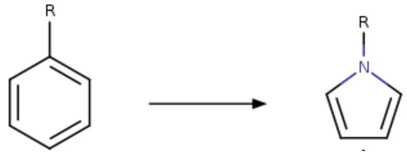
t, o, m, p</td><td>Ring substitution</td><td>[*:6]-[c;D3:1]1[c;D2][c;x2:2][c;x2:3][c;x2:4][c;x2:5]1>>[*:6]-[#7:1]-1-[#6;x2:5]=[#6;x2:4]-[#6;x2:3]=[#6;x2:2]-1 |c:9,11,rb:3:2,4:2,5:2,6:2,9:2,10:2,11:2,12:2,s:1:3|</td><td>-Enhanced solubility</td><td>(Dalvie et al., 2002; Subbaiah and Meanwell, 2021)</td></tr>
<tr><td>Phenyl</td><td>Phenyl_to_furanyl
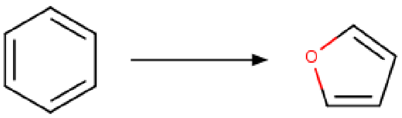
t, o, m, p</td><td>Ring substitution</td><td>[c;x2:5]1[c;D2:1][c;D2][c;x2:2][c;x2:3][c;x2:4]1>>[#8:1]-1-[*;x2:5]=[*;x2:4]-[*;x2:3]=[*;x2:2]-1 |c:7,9,rb:0:2,3:2,4:2,5:2,7:2,8:2,9:2,10:2,s:1:2|</td><td>-Enhanced solubility</td><td>(Dalvie et al., 2002; Subbaiah and Meanwell, 2021)</td></tr>
<tr><td>Phenyl</td><td>Phenyl_to_thienyl
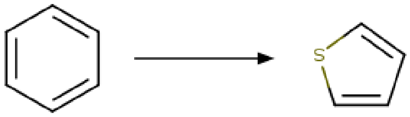
t, o, m, p</td><td>Ring substitution</td><td>[c;x2:5]1[c;D2:1][c;D2][c;x2:2][c;x2:3][c;x2:4]1>>[#16:1]-1-[*;x2:5]=[*;x2:4]-[*;x2:3]=[*;x2:2]-1 |c:7,9,rb:0:2,3:2,4:2,5:2,7:2,8:2,9:2,10:2,s:1:2|</td><td>-Enhanced solubility</td><td>(Dalvie et al., 2002; Subbaiah and Meanwell, 2021)</td></tr>
<tr><td>Phenyl</td><td>Phenyl_to_oxazoles
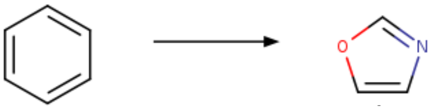
t, o, m, p</td><td>Ring substitution</td><td>[c;x2:4]1[c;D2][c;D2:3][c;x2:2][c;D2:1][c;x2:5]1>>[#8:1]-1-[*:2]=[#7:3]-[*:4]=[*:5]-1 |c:7,9,rb:0:2,3:2,5:2,s:2:2,4:2|</td><td>-Increased polarity (the measured properties is dependent on the topology)
-Improved potency
-Reduced lipophilicity
-Increased membrane permeability</td><td>(Kotoku et al., 2019; Subbaiah and Meanwell, 2021)</td></tr>
<tr><td>Phenyl</td><td>Phenyl_to_thiazoles
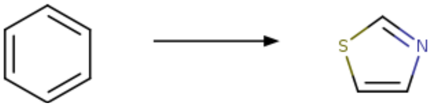
t, o, m, p</td><td>Ring substitution</td><td>[c;x2:5]1[c;x2:4][c;D2:3][c;x2:2][c;D2:1][c;x2D2]1>>[#16:1]-1-[*:2]=[#7:3]-[*:4]=[*:5]-1 |c:7,9,rb:0:2,1:2,3:2,5:2,s:2:2,4:2|</td><td>-Increased polarity (the measured properties is dependent on the topology)
-Metabolic stability
-Improved potency
-Reduced lipophilicity
-Increased membrane permeability</td><td>(Dossetter et al., 2012; Kotoku et al., 2019; Subbaiah and Meanwell, 2021)</td></tr>
<tr><td>Phenyl</td><td>Phenyl_to_isoxazoles
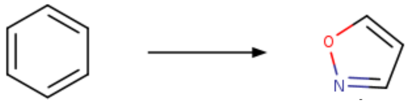
t, o, m, p</td><td>Ring substitution</td><td>[c;x2:4]1[c;D2][c;x2:3][c;D2:2][c;D2:1][c;x2:5]1>>[#8:1]-1-[#7:2]=[*:3]-[*:4]=[*:5]-1 |c:7,9,rb:0:2,2:2,5:2,s:3:2,4:2|</td><td>-Bioisosteric replacement
-Improved potency
-Reduced lipophilicity
-Increased membrane permeability</td><td>(Kotoku et al., 2019; Subbaiah and Meanwell, 2021)</td></tr>
</table>

<table>
<tr><td>Phenyl</td><td>Phenyl_to _imidazole
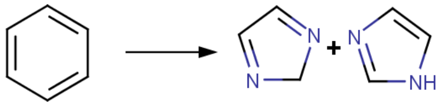
t, o, m, p</td><td>Ring substitution</td><td>[c;x2;D2:1]1[c;x2:2][c;x2;D2:3][c;D2][c;x2:4][c;x2:5]1>>[#7:1]-1-[*:2]=[#7:3]-[*:4]=[*:5]-1 |c:7,9,s:0:2,2:2|</td><td>-The effect on solubility was dependent upon the regioisomer topology
-Improved potency
-Reduced CYP3A4 TDI
-Metabolic stability</td><td>(Dossetter et al., 2012; Bonazzi et al., 2020; Subbaiah and Meanwell, 2021)</td></tr>
<tr><td>Phenyl</td><td>Phenyl_to _imidazole1
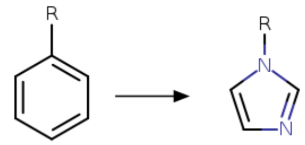
t, o, m, p</td><td>Ring substitution</td><td>[*:6]-[c;x2D3:1]1[c;x2:5][c;x2:4][c;D2][c;x2D2:3][c;x2:2]1>>[*:6]-[#7:1]-1-[*:2]=[#7:3]-[*:4]=[*:5]-1 |c:9,11,rb:1:2,2:2,3:2,5:2,6:2,s:1:3,5:2|</td><td>-Bioisosteric replacement
The effect on solubility was dependent upon the regioisomer topology</td><td>(Ritchie and Macdonald, 2016; Subbaiah and Meanwell, 2021)</td></tr>
<tr><td>Phenyl</td><td>Phenyl_to_pyrazol
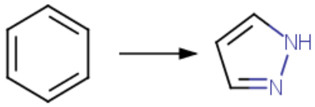
t, o, m, p</td><td>Ring substitution</td><td>[c;x2:5]1[c;x2D2][c;x2:4][c;x2:3][c;x2D2:2][c;x2:1]1>>[#7;x2:1]-1-[#7:2]=[*:3]-[*:4]=[*:5]-1 |c:7,9,rb:0:2,1:2,2:2,3:2,4:2,6:2,s:5:2,4:2|</td><td>-Bioisosteric replacement
-Reduced lipophilicity,
-Higher LLE
-Reduced hLiMT cytotoxicity
-Reduced CYP3A4 TDI</td><td>(Liang et al., 2016; McKerrall et al., 2019; Bonazzi et al., 2020; Subbaiah and Meanwell, 2021)</td></tr>
<tr><td>Phenyl</td><td>Phenyl_to_pyrazol1
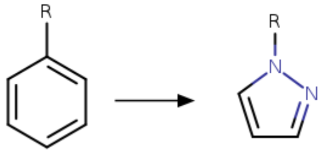
t, o, m, p</td><td>Ring substitution</td><td>[*:6]-[c;x2D3:1]1[c;x2:2][c;x2D2][c;x2:3][c;x2:4][c;x2D2:5]1>>[*:6]-[#7:1]-1-[#6:2]=[*:3]-[*:4]=[#7:5]-1 |c:9,11,rb:1:2,2:2,3:2,4:2,5:2,6:2,s:1:3,6:2|</td><td>-Bioisosteric replacement
- Reduced hERG liability
-Reduced lipophilicity</td><td>(Deng et al., 2015; Ritchie and Macdonald, 2016; Subbaiah and Meanwell, 2021)</td></tr>
<tr><td>Phenyl</td><td>Phenyl_to _triazole1
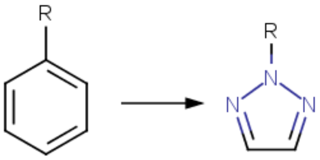
t, m, p</td><td>Ring substitution</td><td>[*:6]-[c;x2D3:1]1[c;x2D2:2][c;x2D2][c;x2:3][c;x2:4][c;x2D2:5]1>>[*:6]-[#7:1]-1-[#7:2]=[#6:3]-[#6:4]=[#7:5]-1 |c:9,11,rb:1:2,2:2,3:2,4:2,5:2,6:2,s:1:3,2:2,6:2|</td><td>-Bioisosteric replacement</td><td>(Subbaiah and Meanwell, 2021)</td></tr>
<tr><td>Phenyl</td><td>Phenyl_to _triazole
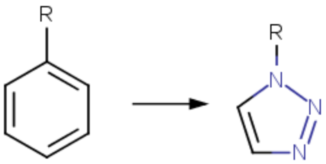
t, o, m</td><td>Ring substitution</td><td>[*:6]-[c;x2D3:1]1[c;x2D2:2][c;x2D2][c;x2D2:3][c;x2:4][c;x2:5]1>>[*:6]-[#7:1]-1-[#6:5]=[#6:4]-[#7:3]=[#7:2]-1 |c:9,11,rb:1:2,2:2,3:2,4:2,5:2,6:2,s:1:3,2:2,4:2|</td><td>-Bioisosteric replacement</td><td>(Subbaiah and Meanwell, 2021)</td></tr>
<tr><td>Phenyl</td><td>Phenyl_to _triazole1
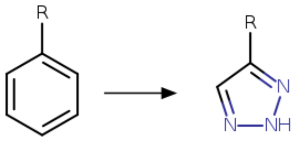
t, o</td><td>Ring substitution</td><td>[c;D2;x2:1]1[c;x2D2:2][c;x2:3][c;x2:4][c;D2][c;x2D2:5]1>>[#7;x2:1]-1-[#7:5]=[*:4]-[*:3]=[#7;D2:2]-1 |c:7,9,rb:1:2,2:2,3:2,6:2,s:5:2,1:2,0:2|</td><td>-Bioisosteric replacement
-Improved potency
-Reduced CYP3A4 TDI</td><td>(Bonazzi et al., 2020; Subbaiah and Meanwell, 2021)</td></tr>
</table>

<table>
<tr><td>Phenyl</td><td>Phenyl_to_oxadiazole
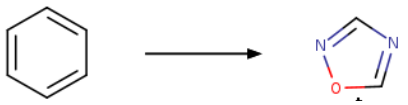
t, p</td><td>Ring substitution</td><td>[c;x2:1]1[c;x2D2][c;x2D2:5][c;x2:4][c;x2D2:3][c;x2D2:2]1>>[#8:3]-1-[#6:4]=[#7:5]-[#6:1]=[#7:2]-1 |c:7,9,rb:0:2,1:2,2:2,3:2,4:2,5:2,s:2:2,4:2,5:2|</td><td>-Bioisosteric replacement
-Metabolic stability
-Improved potency
-Reduced lipophilicity
-Increased membrane permeability</td><td>(Kotoku et al., 2019; Subbaiah and Meanwell, 2021; Camci and Karali, 2023)</td></tr>
<tr><td>Phenyl</td><td>Phenyl_to_oxadiazole_1
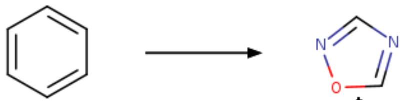
m</td><td>Ring substitution</td><td>[*:6]-[c;x2:1]1[c;x2D2:5][c;x2D2:4][c;x2D2][c;x2:3](-[*:7])[c;x2D2:2]1>>[*:7]-[#6:3]-1=[#7:2]-[#6:1](-[*:6])=[#7:5]-[#8:4]-1 |c:12,rb:1:2,2:2,3:2,4:2,5:2,7:2,s:2:2,3:2,7:2|</td><td>-Bioisosteric replacement

-Metabolic stability</td><td>(Kotoku et al., 2019; Subbaiah and Meanwell, 2021; Camci and Karali, 2023)</td></tr>
<tr><td>Phenyl</td><td>Phenyl_to_oxadiazole2
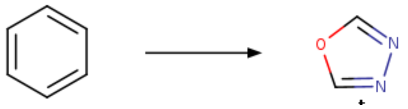
t, p</td><td>Ring substitution</td><td>[c;x2D2:2]1[c;x2D2:3][c;x2:4][c;x2D2:5][c;x2D2][c;x2:1]1>>[#8:5]-1-[#6:4]=[#7:3]-[#7:2]=[#6:1]-1 |c:7,9,rb:0:2,1:2,2:2,3:2,4:2,5:2,s:0:2,1:2,3:2|</td><td>-Bioisosteric replacement

-Metabolic stability
-Reduced lipophilicity</td><td>(Dossetter et al., 2012; Subbaiah and Meanwell, 2021)</td></tr>
<tr><td>Phenyl</td><td>Phenyl_to_oxadiazole_3
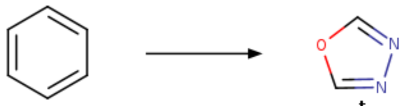
m</td><td>Ring substitution</td><td>[#6:6]-[c;x2:1]1[c;x2D2][c;D2:5][c;x2D2:4][c;x2:3](-[#6:7])[c;x2D2:2]1>>[#6:6]-[#6:1]-1=[#7:5]-[#7:4]=[#6:3](-[#6:7])-[#8:2]-1 |rb:1:2,2:2,4:2,5:2,7:2,s:3:2,4:2,7:2|</td><td>-Bioisosteric replacement

-Metabolic stability</td><td>(Dossetter et al., 2012; Subbaiah and Meanwell, 2021)</td></tr>
<tr><td>Phenyl</td><td>Phenyl_to _triazole2
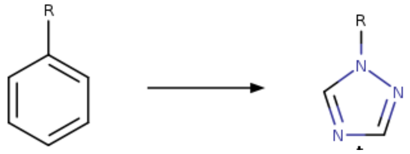
t, o, m, p</td><td>Ring substitution</td><td>[c;D2,D3;x2:1]1[c;x2:2][c;x2D2:3][c;x2:4][c;D2][c;D2:5]1>>[#7;x2:1]-1-[#7:5]=[*:4]-[#7:3]=[*:2]-1 |c:7,9,rb:1:2,2:2,3:2,6:2,s:2:2,5:2|</td><td>-Bioisosteric replacement</td><td>(Subbaiah and Meanwell, 2021)</td></tr>
<tr><td>Phenyl</td><td>Phenyl_to_thiadiazole
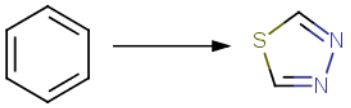
t, p</td><td>Ring substitution</td><td>[c;x2:1]1[c;x2D2:2][c;x2D2:3][c;x2:4][c;D2][c;x2D2:5]1>>[#16:5]-1-[#6:4]=[#7:3]-[#7:2]=[#6:1]-1 |c:7,9,rb:0:2,1:2,2:2,3:2,5:2,s:1:2,2:2,5:2|</td><td>-Bioisosteric replacement

-Metabolic stability</td><td>(Dossetter et al., 2012; Subbaiah and Meanwell, 2021)</td></tr>
<tr><td>Phenyl</td><td>Phenyl_to_thiadiazole
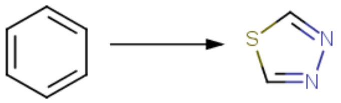
m</td><td>Ring substitution</td><td>[#6:6]-[c;x2:1]1[c;x2D2][c;D2:5][c;x2D2:4][c;x2:3](-[#6:7])[c;x2D2:2]1>>[#6:6]-[#6:1]-1=[#7:5]-[#7:4]=[#6:3](-[#6:7])-[#16:2]-1 |rb:1:2,2:2,4:2,5:2,7:2,s:3:2,4:2,7:2|</td><td>-Bioisosteric replacement
-Improved potency
-Reduced lipophilicity
-Increased membrane permeability</td><td>(Kotoku et al., 2019; Subbaiah and Meanwell, 2021)</td></tr>
<tr><td>Phenyl</td><td>Phenyl_to_thiadiazole2
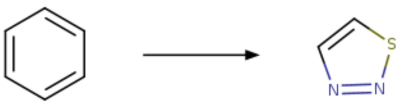
t, o</td><td>Ring substitution</td><td>[c;x2:1]1[c;x2:2][c;x2D2:3][c;x2D2][c;x2D2:4][c;x2D2:5]1>>[#16:5]-1-[#6:1]=[#6:2]-[#7:3]=[#7:4]-1 |c:7,9,rb:0:2,1:2,2:2,3:2,5:2,s:2:2,4:2,5:2|</td><td>-Bioisosteric replacement

-Metabolic stabilit</td><td>(Dossetter et al., 2012; Subbaiah and Meanwell, 2021)</td></tr>
<tr><td>Phenyl</td><td>Phenyl_to_cyclohexane</td><td>Ring substitution</td><td>[c;x2:2]1[c;x2:3][c;x2:4][c;x2:5][</td><td>-Increase</td><td>(Press et al.,</td></tr>
</table>

	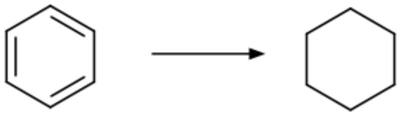<br>t, o, m, p		c;x2:6][c;x2:1]1>>[#6:5]-1-[#6:6]-[#6:1]-[#6:2]-[#6:3]-[#6:4]-1	lipophilicity<br>-Improved aqueous solubility<br>-Enhanced oral bioavailability<br>-Reproducible PK profiles	2012; Press, Neil J. et al., 2015; Subbaiah and Meanwell, 2021)
Phenyl	Phenyl_to_cyclohexene<br>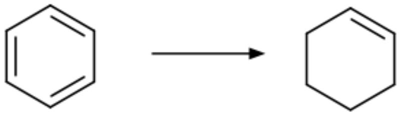<br>t, o, m, p	Ring modification	[c;x2:1]1[c;x2:6][c;x2:5][c;x2:4][c;D2:3][c;D2:2]1>>[#6:4]-1-[#6:3]-[#6:2]-[#6:1]=[#6:6]-[#6:5]-1	-Improved potency<br>-Enhanced selectivity<br><br>* Reactive structure	(Schnider et al., 2020; Subbaiah and Meanwell, 2021)
Phenyl	Phenyl_to_bicyclo[4.1.0]heptane<br>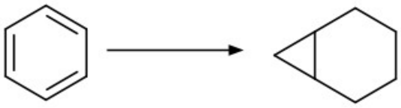<br>t, o, m, p	Ring substitution	[c;x2:2]1[c;x2:3][c;x2:4][c;x2:5][c;x2:6][c;x2:1]1>>[#6]-1-[#6:1]-2-[#6:2]-[#6:3]-[#6:4]-[#6:5]-[#6:6]-1-2	-Bioisosteric replacement<br>-Used to eliminate release of an aniline as a potential mutagenic liability	(Xu et al., 2006; Subbaiah and Meanwell, 2021)
Phenyl	Phenyl_to_cyclopentane<br>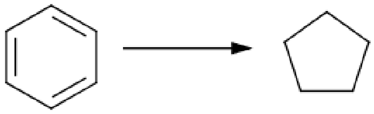<br>t, o, m, p	Ring substitution	[c;D2]1[c;x2:5][c;x2:4][c;x2:3][c;x2:2][c;x2:1]1>>[#6:2]-1-[#6:3]-[#6:4]-[#6:5]-[#6:1]-1	-Increase lipophilicity	(Ritchie and Macdonald, 2016; Subbaiah and Meanwell, 2021)
Phenyl	Phenyl_to_cyclobutyl<br>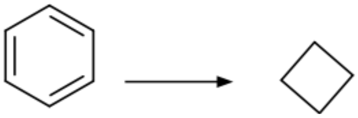<br>t, o, p	Ring substitution	[c;x2D2]1[c;D2,D3;x2:2][c;x2D3:1][c;x2D2][c;x2D2][c;D2,D3;x2:3]1>>[#6]-1-[#6:3]-[#6:2]-[#6:1]-1	-Reduced *in vitro* clearance<br>-Abrogation of potential safety issues associated with DNA binding<br>-Reduced potential to cause PL	(Wager et al., 2011; Subbaiah and Meanwell, 2021)
Phenyl	Phenyl_to_cyclopropanyl<br>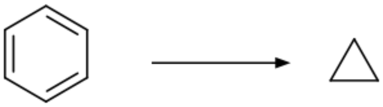<br>t, m, p	Ring substitution	[c;D2,D3;x2:3]1[c;x2D2][c;x2D2][c;x2D3:1][c;x2D2][c;D2,D3;x2:2]1>>[#6:3]-1-[#6:1]-[#6:2]-1	-Bioisosteric replacement<br>-Improved potency<br>-Reduced hydrophobicity<br>-Increased Fsp^3 content	(Abe et al., 2011; Cumming et al., 2012; Winneroski et al., 2020; Subbaiah and Meanwell, 2021)
Phenyl	Phenyl_to_BCP (bicyclo[1.1.1]pentane)<br>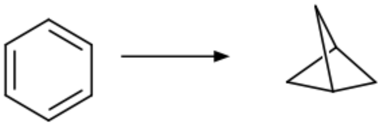<br>t, o, p	Ring substitution	[c;x2:3]1[c;x2D2][c;x2D2][c;x2:1][c;x2:2][c;x2D2]1>>[#6]-1-[#6:1]-2-[#6:2]-[#6:3]-1-[#6]-2 |rb:0:2,1:2,2:2,3:2,4:2,5:2|	-Bioisosteric replacement<br>-Increased Fsp^3 content<br>-Improved potency<br>-Improved solubility	(Nicolaou et al., 2016; Subbaiah and Meanwell, 2021; Zhao et al., 2021)

Phenyl	Phenyl_to_bicyclo[2.1.1]hexane<br>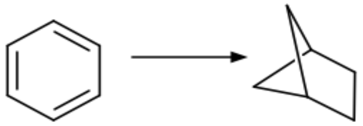<br>t, o, p	Ring substitution	[c;x2:3]1[c;x2D2][c;x2D2][c;x2:1][c;x2:2][c;x2D2]1>>[#6]-1-[#6:3]-2-[#6:2]-[#6:1]-1-[#6]-[#6]-2 |rb:0:2,1:2,2:2,3:2,4:2,5:2|	-Bioisosteric replacement<br>-Increased Fsp^3 content	(Subbaiah and Meanwell, 2021)
Phenyl	Phenyl_to_CUB<br>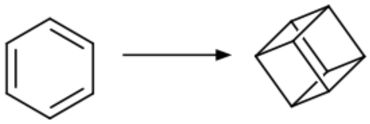<br>t, o, p	Ring substitution	[c;x2:3]1[c;x2D2][c;x2D2][c;x2:1][c;x2:2][c;x2D2]1>>[#6;x2]-1-2-[#6:2]-3-[#6;x2:1]-4-[#6]-1-[#6;x2]-1-[#6;x2:3]-2-[#6]-3-[#6]-4-1 |rb:0:2,1:2,2:2,3:2,4:2,5:2,6:2,8:2,10:2,11:2|	-Bioisosteric replacement<br>-Increased Fsp^3 content<br>-Reduced $cLogD_{7.4}$<br>-Reduced MP<br>-Improved aqueous solubility	(Nicolaou et al., 2016; Subbaiah and Meanwell, 2021)
Phenyl	Phenyl_to_BCO (bicyclo[2.2.2]octane)<br>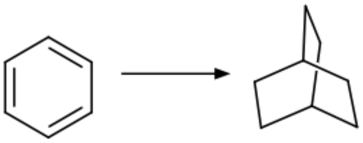<br>t, o, m, p	Ring substitution	[c;x2:5]1[c;x2:1][c;x2:6][c;x2:2][c;x2:3][c;x2:4]1>>[#6:5]-1-[#6]-[#6:1]-2-[#6:2]-[#6:3]-[#6:4]-1-[#6]-[#6]-2 |rb:0:2,3:2,4:2,5:2|	-Bioisosteric replacement<br>-Improved potency<br>-Improved solubility<br>-Reduced PPB	(Kiesman et al., 2006; Iwaki et al., 2020; Subbaiah and Meanwell, 2021)
Phenyl	Phenyl_to_norbornane (bicyclo[2.2.1]heptane)<br>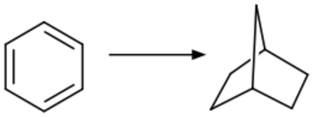<br>t, o, m, p	Ring substitution	[c;x2:4]1[c;x2:3][c;x2:2][c;x2:6][c;x2:1][c;x2:5]1>>[#6:3]-1-[#6:2]-[#6:1]-2-[#6:6]-[#6:5]-[#6:4]-1-[#6]-2<br>|rb:0:2,1:2,2:2,3:2,4:2,5:2|	-Bioisosteric replacement<br>-Improved potency<br>-Iimproved solubility<br>-Reduced PPB	(Iwaki et al., 2020; Subbaiah and Meanwell, 2021)
Phenyl	Phenyl_to_adamantane<br>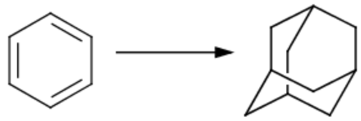<br>t, o, m, p	Ring substitution	[c;x2:5]1[c;x2:6][c;x2:1][c;x2:2][c;x2:3][c;x2:4]1>>[#6]-1-[#6]-2-[#6]-[#6:2]-3-[#6:1]-[#6:6]-1-[#6:5]-[#6:4](-[#6]-2)-[#6:3]-3<br>|rb:0:2,3:2,4:2,5:2|	-Increased sp^3 character<br>-Improved potency	(Wang et al., 2020; Subbaiah and Meanwell, 2021)
Phenyl	Phenyl_to_spiro[3.3] heptane<br>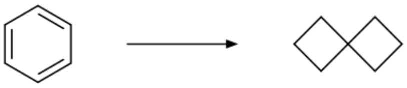<br>t, m, p	Ring substitution	[*:4]-[c;x2D3:1]1[c;x2D2][c;x2D2][c;x2:3][c;x2:2][c;x2D2]1>>[*:4]-[#6:1]-1-[#6]C2([#6:2]-[#6:3]-[#6]2)[#6]-1<br>|rb:1:2,2:2,3:2,4:2,5:2,6:2|	-Bioisosteric replacement<br>-Increased Fsp^3 content	(Subbaiah and Meanwell, 2021; Prysiazhniuk et al., 2023)
Phenyl	Phenyl_to_spiroheptene<br>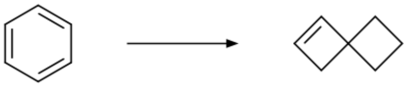<br>t, o, m, p	Ring substitution	[c;x2:4]1[c;x2:3][c;x2:2][c;x2:1][c;x2:6][c;x2:5]1>>[#6:4]-1-[#6:5]C2([#6:3]-1)[#6:6]-[#6:1]=[#6:2]2<br>|c:12,rb:0:2,1:2,2:2,3:2,4:2,5:2|	-Bioisosteric replacement<br>-Increased Fsp^3 content<br><br>* Reactive structure	(Subbaiah and Meanwell, 2021; Swidorski et al., 2021)
Phenyl	Phenyl_to_tetrahydropyran<br>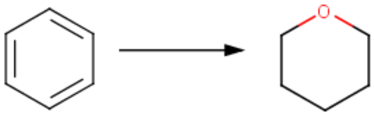<br>t, o, m, p	Ring substitution	[c;x2:5]1[c;x2:6][c;D2:1][c;x2:2][c;x2:3][c;x2:4]1>>[#6:4]-1-[#6:5]-[#6:6]-[#8:1]-[#6:2]-[#6:3]-1<br>|rb:0:2,1:2,3:2,4:2,5:2,s:2:2|	-Bioisosteric replacement<br>-Lower intrinsic clearance in liver microsomes<br>-Reduced lipophilicity<br>-Lower TDI of CYP3A4<br>-Reduced metabolic activation	(Wang et al., 2018; Subbaiah and Meanwell, 2021)

Phenyl	Phenyl_to_3_6_dihydro_2H_pyran<br>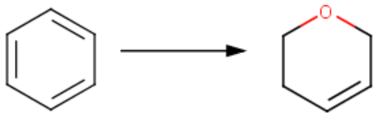<br>t, o, m, p	Ring substitution	[c;x2:5]1[c;D2:1][c;D2][c;x2:2][c;x2:3][c;x2:4]1>>[#6]-1-[#6:5]-[#6:4]=[#6:3]-[#6:2]-[#8:1]-1	-Bioisosteric replacemen<br><br>* Reactive structure	(Subbaiah and Meanwell, 2021)
Phenyl	Phenyl_to_dioxane<br>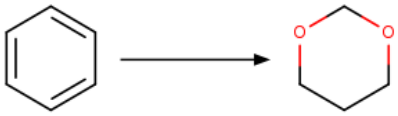<br>t, o, m, p	Ring substitution	[c;x2:5]1[c;x2:6][c;D2:1][c;x2:2][c;x2:3][c;x2:4]1>>[#6:5]-1-[#6:4]-[#8:3]-[#6:2]-[#8:1]-[#6:6]-1 |rb:0:2,1:2,3:2,4:2,5:2,s:2:2,4:2|	lower intrinsic clearance in liver microsomes<br>-Reduced lipophilicity<br>-Lower TDI of CYP3A4<br>-Reduced metabolic activation	(Wang et al., 2018; Subbaiah and Meanwell, 2021)
Phenyl	Phenyl_to_tetrahydrofuran<br>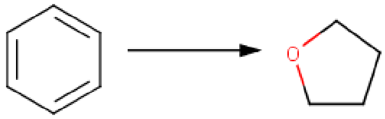<br>t, o, m, p	Ring substitution	[c;x2:5]1[c;D2][c;D2:1][c;x2:2][c;x2:3][c;x2:4]1>>[#6:4]-1-[#6:3]-[#6:2]-[#8:1]-[#6:5]-1 |rb:0:2,1:2,3:2,4:2,5:2,s:2:2|	-Lower intrinsic clearance in liver microsomes<br>-Reduced lipophilicity<br>-Lower TDI of CYP3A4<br>-Reduced metabolic activation	(Wang et al., 2018; Subbaiah and Meanwell, 2021)
Phenyl	Phenyl_to_dioxolane<br>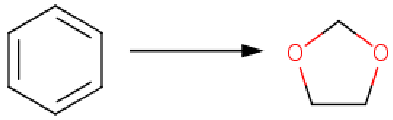<br>t, o, m, p	Ring substitution	[c;x2:5]1[c;D2][c;x2D2:1][c;x2:2][c;x2D2:3][c;x2:4]1>>[#6:5]-1-[#6:4]-[#8:3]-[#6:2]-[#8:1]-1 |rb:0:2,1:2,3:2,4:2,5:2,s:2:2,4:2|	-Bioisosteric replacement	(Subbaiah and Meanwell, 2021)
Phenyl	Phenyl_to_oxetane<br>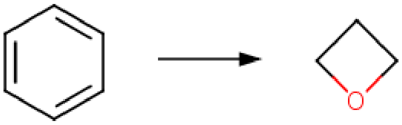<br>t, o, p	Ring substitution	[c;x2:4]1[c;x2D2][c;x2D2:1][c;x2:2][c;x2:3][c;x2D2]1>>[#6:3]-1-[#6:2]-[#8:1]-[#6:4]-1 |rb:0:2,1:2,2:2,3:2,4:2,5:2,s:2:2|	-Bioisosteric replacement	(Burkhard et al., 2010; Wuitschik et al., 2010; Subbaiah and Meanwell, 2021)
Phenyl	Phenyl_to_dioxepane<br>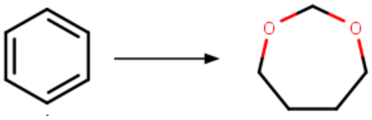<br>t, o, m, p	Ring substitution	[c;x2:4]1[c;x2D2:3][c;x2:2][c;D2:1][c;x2:6][c;x2:5]1>>[#6]-1-[#6:5]-[#6:6]-[#8:1]-[#6:2]-[#8:3]-[#6:4]-1 |rb:0:2,1:2,2:2,4:2,5:2,s:1:2,3:2|	-Lower intrinsic clearance in liver microsomes<br>-Reduced lipophilicity<br>-Lower TDI of CYP3A4<br>-Reduced metabolic activation	(Wang et al., 2018; Subbaiah and Meanwell, 2021)
Phenyl	Phenyl_to_oxabicyclo[2.1.1]hexane<br>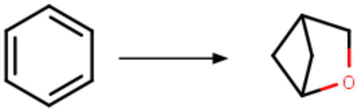<br>t, o, m, p	Ring substitution	[c;x2:4]1[c;x2D2:5][c;x2:1][c;x2:2][c;x2:3][c;x2D2]1>>[#6:2]-1-[#6:3]-2-[#6]-[#6:1]-1-[#8:5]-[#6:4]-2 |rb:0:2,2:2,3:2,4:2,5:2,s:1:2|	-Improved solubility<br>-Increased Fsp^3<br>-Lower lipophilicity	(Levterov et al., 2020; Subbaiah and Meanwell, 2021)

Phenyl	Phenyl_to_oxabicyclo[2.2.2]octane 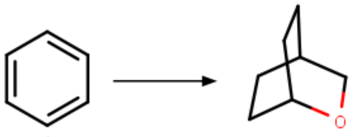 t, o, m, p	Ring substitution	[c;x2:5]1[c;x2:6][c;x2;D3:1][c;x2:2][c;x2D2:3][c;x2:4]1>>[#6]-1-[#6]-[#6:4]-2-[#6:5]-[#6:6]-[#6:1]-1-[#6:2]-[#8:3]-2 |rb:0:2,1:2,2:2,3:2,4:2,5:2,s:4:2|	-Improved solubility<br>-Decrease of clogP<br>-Increased the metabolic stability<br>-Increased the life half time<br>-Improved lipophilicity	(Subbaiah and Meanwell, 2021; Mykhailiuk et al., 2023)
Phenyl	Phenyl_thieno_3_2_b_thiophene 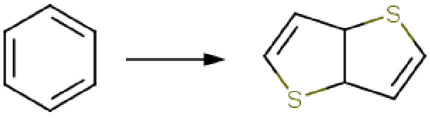 t, o, m, p	Ring substitution	[c;x2:2]1[c;D2;x2:3][c;x2:4][c;x2:5][c;D2:6][c;x2:1]1>>[#16:3]-1-[#6:4]=[#6:5]-[#6]-2=[#6]-1-[#6:2]=[#6:1]-[#16:6]-2 |c:7,9,12,s:1:2,4:2|	-Bioisosteric replacement<br><br>-Improved potency	(Bilello et al., 2014; Subbaiah and Meanwell, 2021)
Phenyl	phenyl_to_pyrazolo_3_2_b_1_3_thiazole 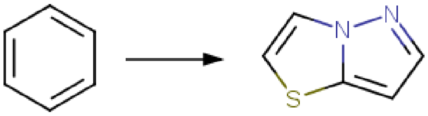 t, m, p	Ring substitution	[c;x2:1]1[c;D2:6][c;x2:5][c;x2:4][c;x2D2:3][c;x2:2]1>>[#16:3]-1-[#6:4]=[#6:5]-[#7]-2-[#7:6]=[#6:1]-[#6:2]=[#6]-1-2 |rb:0:2,2:2,3:2,4:2,5:2,s:1:2,4:2|	-Bioisosteric replacement	(Subbaiah and Meanwell, 2021)
Phenyl	Phenyl_to_pyrazolo_3_2_b_1_3_oxazole 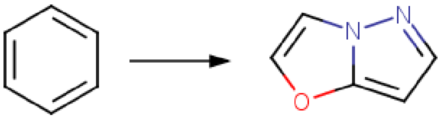 t, m, p	Ring substitution	[c;x2:1]1[c;D2:6][c;x2:5][c;x2:4][c;x2D2:3][c;x2:2]1>>[#8:3]-1-[#6:4]=[#6:5]-[#7]-2-[#7:6]=[#6:1]-[#6:2]=[#6]-1-2 |t:12,rb:0:2,2:2,3:2,4:2,5:2,s:1:2,4:2|	-Bioisosteric replacement	(Subbaiah and Meanwell, 2021)
Phenyl	Phenyl_to_imidazo_2_1_b_thiazole 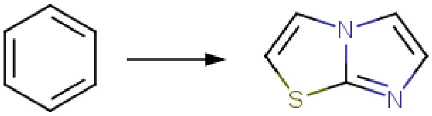 t, o, m, p	Ring substitution	[c;x2:1]1[c;x2:6][c;x2:5][c;x2:4][c;x2D2:3][c;x2D2:2]1>>[#16:3]-1-[#6:4]=[#6:5]-[#7]-2-[#6:6]=[#6:1]-[#7:2]=[#6]-1-2 |t:12,rb:0:2,2:2,3:2,4:2,5:2,s:4:2,5:2|	-Bioisosteric replacement	(Subbaiah and Meanwell, 2021)
Phenyl	Phenyl_to_1_3_thiazolo_4_5_d_1_3_thiazole 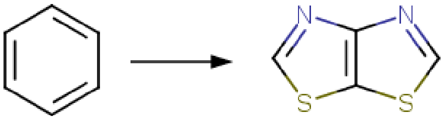 t, p	Ring substitution	[c;x2:1]1[c;x2D2:6][c;x2D2:5][c;x2:4][c;x2D2:3][c;x2D2:2]1>>[#16:5]-1-[#6:4]=[#7:3]-[#6]-2=[#6]-1-[#16:6]-[#6:1]=[#7:2]-2 |c:7,13,rb:0:2,1:2,2:2,3:2,4:2,5:2,s:1:2,2:2,4:2,5:2|	-Bioisosteric replacement	(Subbaiah and Meanwell, 2021)
Phenyl	Phenyl_to_1_3_thiazolo_5_4_d_1_3_thiazole 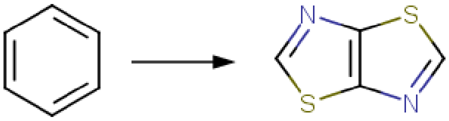 t, p	Ring substitution	[c;x2:1]1[c;x2D2:6][c;x2D2:5][c;x2:4][c;x2D2:3][c;x2D2:2]1>>[#16:5]-1-[#6:4]=[#7:3]-[#6]-2=[#6]-1-[#7:6]=[#6:1]-[#16:2]-2 |c:7,9,12,rb:0:2,1:2,2:2,3:2,4:2,5:2,s:1:2,2:2,4:2,5:2|	-Bioisosteric replacement	(Subbaiah and Meanwell, 2021)
Phenyl	Phenyl_to__1_3_oxazolo_4_5_d_1_3_oxazole 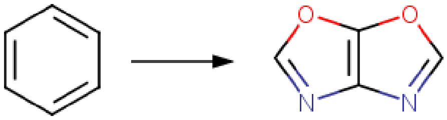 t, p	Ring substitution	[c;x2:1]1[c;x2D2:6][c;x2D2:5][c;x2:4][c;x2D2:3][c;x2D2:2]1>>[#8:5]-1-[#6:4]=[#7:3]-[#6]-2=[#6]-1-[#8:6]-[#6:1]=[#7:2]-2 |c:7,13,rb:0:2,1:2,2:2,3:2,4:2,5:2,s:1:2,2:2,4:2,5:2|	-Bioisosteric replacement	(Subbaiah and Meanwell, 2021)

<table>
<tr><th></th><th></th><th></th><th></th><th></th><th></th></tr>
<tr><td>Phenyl</td><td>Phenyl_to__1_3_oxazolo_5_4_d_1_3_oxazole
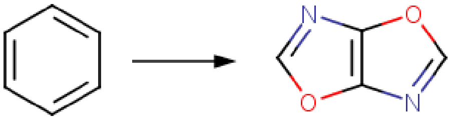

t, p</td><td>Ring substitution</td><td>[c;x2:1]1[c;x2D2:6][c;x2D2:5][c;x2:4][c;x2D2:3][c;x2D2:2]1>>[#8:5]-1-[#6:4]=[#7:3]-[#6]-2=[#6]-1-[#7:6]=[#6:1]-[#8:2]-2 |c:7,9,12,rb:0:2,1:2,2:2,3:2,4:2,5:2,s:1:2,2:2,4:2,5:2|</td><td>-Bioisosteric replacement</td><td>(Subbaiah and Meanwell, 2021)</td></tr>
<tr><td>Phenyl</td><td>Phenyl_to_pyrrolo_1_2_b_pyrazole
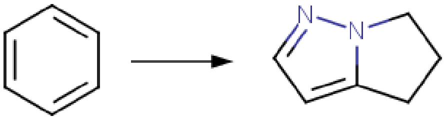

t, o, m, p</td><td>Ring substitution</td><td>[c;x2D2:2]1[c;x2:3][c;x2:4][c;x2:5][c;x2:6][c;x2:1]1>>[#6:4]-1-[#6:3]-[#7]-2-[#7:2]=[#6:1]-[#6]=[#6]-2-[#6:5]-1 |rb:0:2,1:2,2:2,3:2,4:2,5:2,s:0:2|</td><td>-Bioisosteric replacement
-Improved enzyme inhibitory activity and cell potency
-Enhanced aqueous solubility</td><td>(Meanwell and Sistla, 2021; Subbaiah and Meanwell, 2021)</td></tr>
<tr><td>Phenyl</td><td>Phenyl_to__pyrrolo_3_4_c_pyrazole
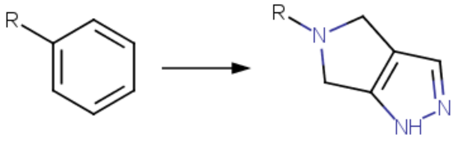

t, o, m</td><td>Ring substitution</td><td>[*:7]-[c;x2D3:1]1[c;x2:6][c;x2:5][c;x2D2:4][c;x2:3][c;x2:2]1>>[*:7]-[#7:1]-1-[#6:6]-[#6]-2=[#6](-[#6:2]-1)-[#6:3]=[#7:4]-[#7:5]-2 |c:10,14,rb:1:2,2:2,3:2,s:1:3,4:2,3:2|</td><td>-Bioisosteric replacement</td><td>(Meanwell and Sistla, 2021; Subbaiah and Meanwell, 2021)</td></tr>
<tr><td>Phenyl</td><td>Phenyl_to__pyrrolo_3_4_c_pyrazole
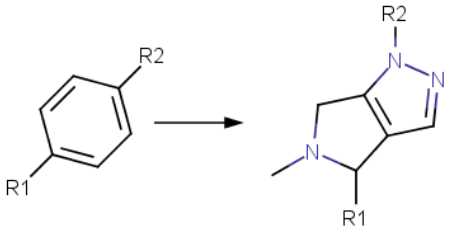
</td><td>Ring substitution</td><td>[*:7]-[c;x2D3:1]1[c;x2:6][c;x2D2:5][c;x2D3:4](-[*:8])[c;x2:3][c;x2:2]1>>[#6]-[#7:6]-1-[#6:5]-[#6]-2=[#6](-[#6:2]=[#7:3]-[#7:4]-2-[*:8])-[#6:1]-1-[*:7] |c:13,t:11,rb:1:2,2:2,3:2,4:2,6:2,7:2,s:1:3,3:2,4:3|</td><td>-Bioisosteric replacement</td><td>(Subbaiah and Meanwell, 2021)</td></tr>
<tr><td>Phenyl</td><td>Phenyl_to_pyrrolo_3_4_d_imidazole
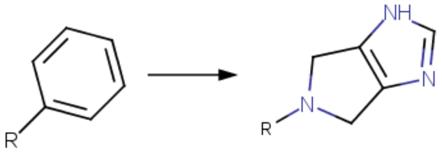

t, o, m, p</td><td>Ring substitution</td><td>[*:7]-[c;x2D3:1]1[c;x2:2][c;x2D2:3][c;x2:4][c:5][c;x2:6]1>>[*:7]-[#7:1]-1-[#6:6]-[#6]-2=[#6](-[#6:2]-1)-[#7:3]=[#6:4]-[#7:5]-2 |c:10,14,rb:1:2,2:2,3:2,4:2,6:2,s:1:3,3:2|</td><td>-Bioisosteric replacement</td><td>(Subbaiah and Meanwell, 2021)</td></tr>
<tr><td>Phenyl</td><td>Phenyl_to_pyrrolo_3_4_d_1_2_3_triazole
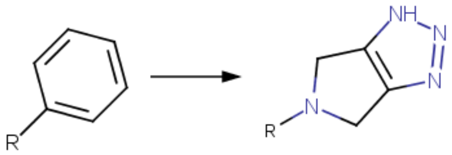

t, o, m</td><td>Ring substitution</td><td>[*:7]-[c;x2D3:1]1[c;x2:6][c:5][c;x2D2:4][c;x2D2:3][c;x2:2]1>>[*:7]-[#7:1]-1-[#6:6]-[#6]-2=[#6](-[#6:2]-1)-[#7:3]=[#7:4]-[#7:5]-2 |c:10,14,rb:1:2,2:2,4:2,5:2,6:2,s:1:3,4:2,5:2|</td><td>-Bioisosteric replacement</td><td>(Subbaiah and Meanwell, 2021)</td></tr>
<tr><td>Phenyl</td><td>Phenyl_to_pyrrolo_3_4_d_1_2_3_triazole
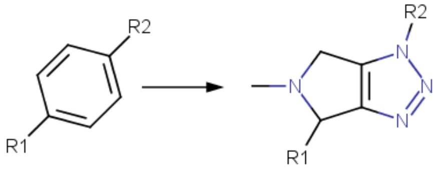
</td><td>Ring substitution</td><td>[*:8]-[c;x2D3:4]1[c;x2:3][c;x2:2][c;x2D3:1](-[*:7])[c;x2:6][c;x2D2:5]1>>[#6]-[#7:6]-1-[#6:5]-[#6]-2=[#6](-[#7:2]=[#7:3]-[#7:4]-2-[*:8])-[#6:1]-1-[*:7] |c:13,t:11,rb:1:2,2:2,3:2,4:2,6:2,7:2,s:1:3,4:3,2:2,3:2|</td><td>-Bioisosteric replacement</td><td>(Subbaiah and Meanwell, 2021)</td></tr>
</table>

<table>
<tr><td>Phenyl</td><td>Phenyl_to_pyrrolo_3_4_d_1_3_oxazole
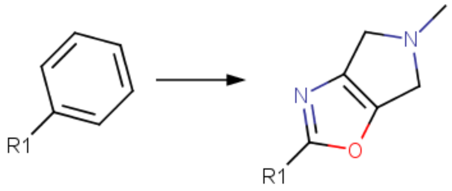
t, m</td><td>Ring substitution</td><td>[c;x2:1]1[c;x2:6][c;x2D2:5][c;x2:4][c;x2D2:3][c;x2:2]1>>[#6]-[#7:1]-1-[#6:6]-[#6]-2=[#6](-[#6:2]-1)-[#7:3]=[#6:4]-[#8:5]-2 |rb:0:2,1:2,2:2,3:2,4:2,5:2,s:0:2,2:2,4:2|</td><td>-Bioisosteric replacement</td><td>(Meanwell and Sistla, 2021; Subbaiah and Meanwell, 2021)</td></tr>
<tr><td>Phenyl</td><td>Phenyl_to_pyrrolo_3_4_d_1_3_oxazole
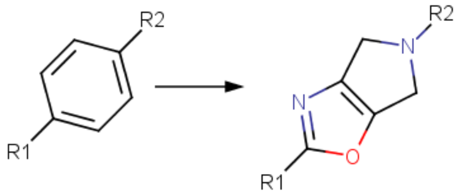</td><td>Ring substitution</td><td>[*:7]-[c;x2:4]1[c;x2D2:5][c;x2:6][c;x2D3:1](-[*:8])[c;x2:2][c;x2D2:3]1>>[*:8]-[#7:1]-1-[#6:6]-[#6]-2=[#6](-[#6:2]-1)-[#7:3]=[#6:4](-[*:7])-[#8:5]-2 |c:11,t:15,rb:1:2,2:2,3:2,4:2,6:2,7:2,s:2:2,4:3,7:2|</td><td>-Bioisosteric replacement</td><td>(Subbaiah and Meanwell, 2021)</td></tr>
<tr><td>Phenyl</td><td>Phenyl_to_pyrrolo_3_4_d_thiazole
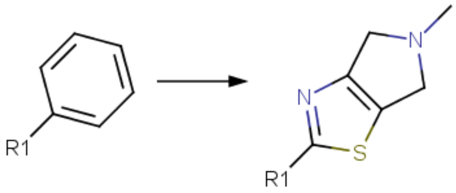
t, m</td><td>Ring substitution</td><td>[c;x2:1]1[c;x2:6][c;x2D2:5][c;x2:4][c;x2D2:3][c;x2:2]1>>[#6]-[#7:1]-1-[#6:6]-[#6]-2=[#6](-[#6:2]-1)-[#7:3]=[#6:4]-[#16:5]-2 |rb:0:2,1:2,2:2,3:2,4:2,5:2,s:0:2,2:2,4:2|</td><td>-Bioisosteric replacement</td><td>(Subbaiah and Meanwell, 2021)</td></tr>
<tr><td>Phenyl</td><td>Phenyl_to_pyrrolo_3_4_d_thiazole
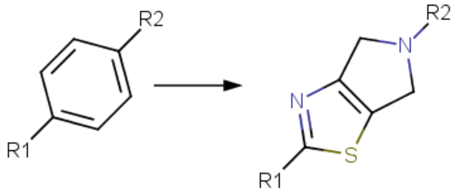</td><td>Ring substitution</td><td>[*:7]-[c;x2:4]1[c;x2D2:5][c;x2:6][c;x2D3:1](-[*:8])[c;x2:2][c;x2D2:3]1>>[*:8]-[#7:1]-1-[#6:6]-[#6]-2=[#6](-[#6:2]-1)-[#7:3]=[#6:4](-[*:7])-[#16:5]-2 |c:11,t:15,rb:1:2,2:2,3:2,4:2,6:2,7:2,s:2:2,4:3,7:2|</td><td>-Bioisosteric replacement</td><td>(Subbaiah and Meanwell, 2021)</td></tr>
<tr><td>Phenyl</td><td>Phenyl_to_azetidine
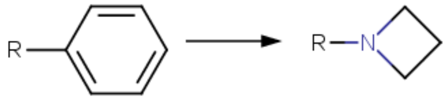
t. o, p</td><td>Ring substitution</td><td>[*:5]-[c;x2:1]1[c;x2:3][c;x2;D2][c;x2:4][c;x2;D2][c;x2:2]1>>[*:5]-[#7:1]-1-[#6:2]-[#6:4]-[#6:3]-1 |s:1:3|</td><td>-Bioisosteric replacement
-Improved solubility
-Enhanced potency
-Reduced LogD
-Increased LLE</td><td>(Ma et al., 2020; Subbaiah and Meanwell, 2021)</td></tr>
<tr><td>Phenyl</td><td>Phenyl_to_azetidine2
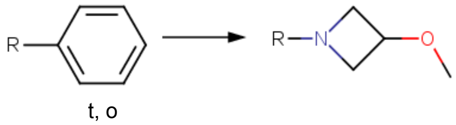
t, o</td><td>Ring substitution</td><td>[*:5]-[c;x2D3:1]1[c;x2:3][c;x2D2][c;D2:4][c;x2D2][c;x2:2]1>>[#6]-[#8]-[#6:4]-1-[#6:2]-[#7:1](-[*:5])-[#6:3]-1 |rb:1:2,2:2,3:2,5:2,6:2,s:1:3|</td><td>-Bioisosteric replacement
-Improved solubility
-Enhanced potency
-Reduced LogD
-Increased LLE</td><td>(Ma et al., 2020; Subbaiah and Meanwell, 2021)</td></tr>
<tr><td>Phenyl</td><td>Phenyl_to_azetidine2_p
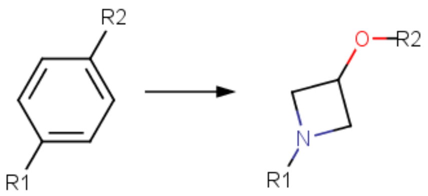</td><td>Ring substitution</td><td>[*:5]-[c;x2D3:1]1[c;x2:2][c;x2D2][c:4](-[*:6])[c;x2D2][c;x2:3]1>>[*:6]-[#8]-[#6:4]-1-[#6:3]-[#7:1](-[*:5])-[#6:2]-1 |rb:1:2,2:2,3:2,6:2,7:2,s:1:3|</td><td>-Improved solubility
-Enhanced potency
-Reduced LogD
-Increased LLE</td><td>(Ma et al., 2020; Subbaiah and Meanwell, 2021)</td></tr>
</table>

<table>
<tr><td>Phenyl</td><td>Phenyl_to_pyrrolidine
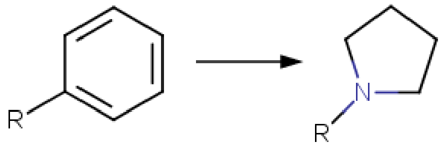

t, o, m, p</td><td>Ring substitution</td><td>[*:6]-[c;x2D3:1]1[c;x2:2][c;x2:3][c;x2:4][c;x2D2][c;x2:5]1>>[*:6]-[#7:1]-1-[#6:2]-[#6:3]-[#6:4]-[#6:5]-1
|rb:1:2,2:2,3:2,5:2,6:2,s:1:3|</td><td>-Bioisosteric replacement
-Improved solubility
-Enhanced potency
-Reduced LogD
-Increased LLE</td><td>(Ma et al., 2020; Subbaiah and Meanwell, 2021)</td></tr>
<tr><td>Phenyl</td><td>Phenyl_to_2_pyrrolidone
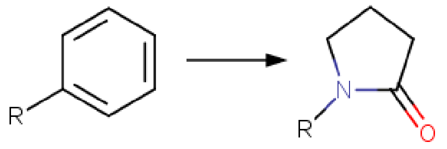

t, o, m, p</td><td>Ring substitution</td><td>[*:6]-[c;x2D3:1]1[c;x2:5][c;x2D2][c;x2:4][c;x2:3][c;x2;D2:2]1>>[*:6]-[#7:1]-1-[#6:5]-[#6:4]-[#6:3]-[#6:2]-1=O
|rb:1:2,2:2,3:2,4:2,5:2,6:2,s:1:3|</td><td>-Bioisosteric replacement
-Reduction of Vd and brain penetration
-Lower potential for PL</td><td>(Mattei et al., 2010; Subbaiah and Meanwell, 2021)</td></tr>
<tr><td>Phenyl</td><td>Phenyl_to_pyrrolidine2
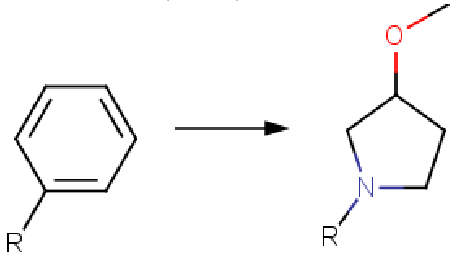

t, o, m</td><td>Ring substitution</td><td>[*:6]-[c;x2D3:1]1[c;x2:5][c;x2D2][c;x2D2:4][c;x2:3][c;x2:2]1>>[#6]-[#8]-[#6:4]-1-[#6:3]-[#6:2]-[#7:1](-[*:6])-[#6:5]-1
|rb:1:2,2:2,3:2,4:2,5:2,6:2,s:1:3|</td><td>-Bioisosteric replacement</td><td>(Subbaiah and Meanwell, 2021)</td></tr>
<tr><td>Phenyl</td><td>phenyl_to_pyrrolidine2p
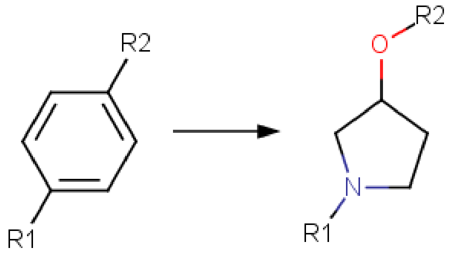
</td><td>Ring substitution</td><td>[*:6]-[c;x2D3:1]1[c;x2D2:5][c;x2D2][c;x2:4](-[*:7])[c;x2D2:3][c;x2D2:2]1>>[*:7]-[#8]-[#6:4]-1-[#6:3]-[#6:2]-[#7:1](-[*:6])-[#6:5]-1
|s:1:3|</td><td>-Bioisosteric replacement</td><td>(Subbaiah and Meanwell, 2021)</td></tr>
<tr><td>Phenyl</td><td>Phenyl_to_piperidine
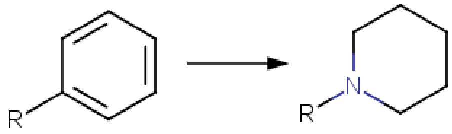
</td><td>Ring substitution</td><td>[*:7]-[c;x2D3:1]1[c;x2:6][c;x2:5][c;x2:4][c;x2:3][c;x2:2]1>>[*:7]-[#7:1]-1-[#6:6]-[#6:5]-[#6:4]-[#6:3]-[#6:2]-1
|rb:1:2,2:2,3:2,4:2,5:2,6:2,s:1:3|</td><td>-Bioisosteric replacement
-Reduced lipophilicity
-Improved aqueous solubility</td><td>(Subbaiah and Meanwell, 2021)</td></tr>
<tr><td>Phenyl</td><td>Phenyl_to_piperidone
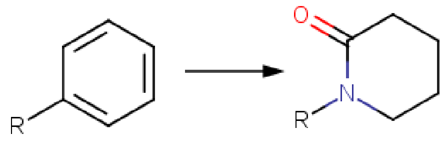

t, o, m, p</td><td>Ring substitution</td><td>[*:7]-[c;x2:1]1[c;D2:2][c;x2:3][c;x2:4][c;x2:5][c;x2:6]1>>[*:7]-[#7:1]-1-[#6:6]-[#6:5]-[#6:4]-[#6:3]-[#6:2]-1=O
|rb:1:2,3:2,4:2,5:2,6:2,s:1:3|</td><td>-Bioisosteric replacement</td><td>(Subbaiah and Meanwell, 2021)</td></tr>
<tr><td>Phenyl</td><td>Phenyl_to_piperidine2
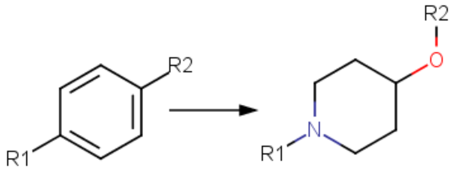
</td><td>Ring substitution</td><td>[*:8]-[c;x2:4]1[c;x2:3][c;x2:2][c;x2D3:1](-[*:7])[c;x2:6][c;x2:5]1>>[*:8]-[#8]-[#6:4]-1-[#6:3]-[#6:2]-[#7:1](-[*:7])-[#6:6]-[#6:5]-1
|rb:1:2,2:2,3:2,4:2,6:2,7:2,s:4:3|</td><td>-Increased sp3 content
-Lower LogP
-Enhanced metabolic stability</td><td>(Shi et al., 2018; Subbaiah and Meanwell, 2021)</td></tr>
</table>

<table>
<tr><td>Phenyl</td><td>Phenyl_to_piperidine2
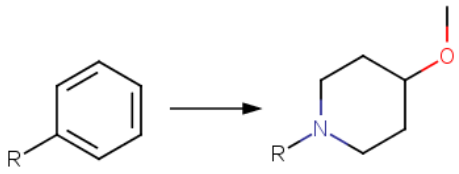
t, o, m</td><td>Ring substitution</td><td>[*:7]-[c;x2D3:1]1[c;x2:5][c;x2:6][c;x2D2:4][c;x2:3][c;x2:2]1>>[#6]-[#8]-[#6:4]-1-[#6:3]-[#6:2]-[#7:1](-[*:7])-[#6:6]-[#6:5]-1 |rb:1:2,2:2,3:2,4:2,5:2,6:2,s:1:3|</td><td>-Increased sp3 content
-Lower LogP
-Enhanced metabolic stability</td><td>(Shi et al., 2018; Subbaiah and Meanwell, 2021)</td></tr>
<tr><td>Phenyl</td><td>Phenyl_to_piperazin_2_one
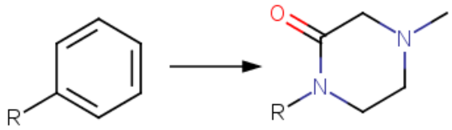
t, o, m</td><td>Ring substitutio</td><td>[*:7]-[c;x2D3:1]1[c;x2:6][c;x2:5][c;x2D2:4][c;x2:3][c;x2D2:2]1>>[#6]-[#7:4]-1-[#6:5]-[#6:6]-[#7:1](-[*:7])-[#6:2](=O)-[#6:3]-1 |rb:1:2,2:2,3:2,4:2,5:2,6:2,s:1:3|</td><td>-Bioisosteric replacement</td><td>(Subbaiah and Meanwell, 2021)</td></tr>
<tr><td>Phenyl</td><td>Phenyl_to_piperazin_2_one
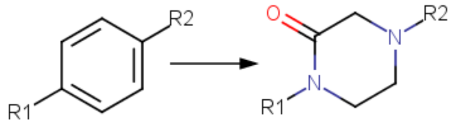</td><td>Ring substitution</td><td>[*:8]-[c;x2:4]1[c;x2:3][c;x2;D2:2][c;x2:1](-[*:7])[c;x2:6][c;x2:5]1>>[*:8]-[#7:4]-1-[#6:5]-[#6:6]-[#7:1](-[*:7])-[#6:2](=O)-[#6:3]-1 |rb:1:2,2:2,3:2,4:2,6:2,7:2,s:1:3,4:3|</td><td>-Bioisosteric replacement</td><td>(Subbaiah and Meanwell, 2021)</td></tr>
<tr><td>Phenyl</td><td>Phenyl_to_piperazine_2_3_dione
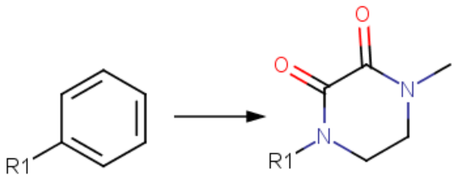
t, o, m</td><td>Ring substitutio</td><td>[*:7]-[c;x2D3:1]1[c;x2:6][c;x2:5][c;x2D2:4][c;x2D2:3][c;x2D2:2]1>>[#6]-[#7:4]-1-[#6:5]-[#6:6]-[#7:1](-[*:7])-[#6:2](=O)-[#6:3]-1=O |rb:1:2,2:2,3:2,4:2,5:2,6:2|</td><td>-Bioisosteric replacement</td><td>(Subbaiah and Meanwell, 2021)</td></tr>
<tr><td>Phenyl</td><td>Phenyl_to_piperazine_2_3_dione
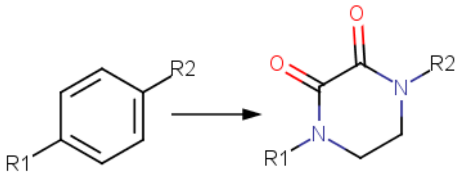</td><td>Ring substitution</td><td>[*:8]-[c;x2:4]1[c;x2;D2:3][c;x2;D2:2][c;x2:1](-[*:7])[c;x2:6][c;x2:5]1>>[*:8]-[#7:4]-1-[#6:5]-[#6:6]-[#7:1](-[*:7])-[#6:2](=O)-[#6:3]-1=O |rb:1:2,2:2,3:2,4:2,6:2,7:2,s:1:3,4:3|</td><td>-Bioisosteric replacement</td><td>(Subbaiah and Meanwell, 2021)</td></tr>
<tr><td>Phenyl</td><td>Phenyl_to_morpholine
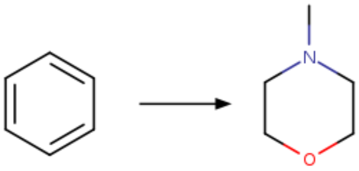
t, o, m</td><td>Ring substitution</td><td>[*:7]-[c;x2:1]1[c;x2:2][c;x2:3][c;x2;D2:4][c;x2:5][c;x2:6]1>>[*:7]-[#7:1]-1-[#6:2]-[#6:3]-[#8:4]-[#6:5]-[#6:6]-1 |rb:1:2,2:2,3:2,4:2,5:2,6:2,s:1:3,4:2|</td><td>-Bioisosteric replacement</td><td>(Subbaiah and Meanwell, 2021)</td></tr>
<tr><td>Phenyl</td><td>Phenyl_to_piperazine
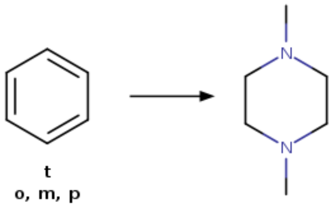
t
o, m, p</td><td>Ring substitution</td><td>[*:7]-[c;x2:1]1[c;x2:6][c;x2:5][c;x2:4](-[*:8])[c;x2:3][c;x2:2]1>>[*:7]-[#7:1]-1-[#6:2]-[#6:3]-[#7:4](-[*:8])-[#6:5]-[#6:6]-1 |rb:1:2,2:2,3:2,4:2,6:2,7:2,s:1:3,4:3|</td><td>-Bioisosteric replacement
-Reduced lipophilicity
-Improved aqueous solubility</td><td>(Subbaiah and Meanwell, 2021)</td></tr>
</table>

Phenyl	Phenyl_to_3_azabicyclo_3_2_1_octane 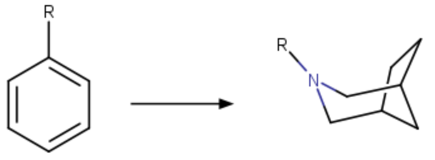 t, o, m, p	Ring substitution	[*:5]-[c;x2D3:1]1[c;x2:2][c;x2D2][c;x2:4][c;x2D2][c;x2:3]1>>[*:5]-[#7:1]-1-[#6:3]-[#6]-2-[#6]-[#6]-[#6](-[#6:4]-2)-[#6:2]-1 |rb:1:2,2:2,3:2,4:2,5:2,6:2,s:1:3|	-Bioisosteric replacement	(Ratni et al., 2021; Subbaiah and Meanwell, 2021)
Phenyl	Phenyl_to_2Azabicyclo_2_2_2_octane 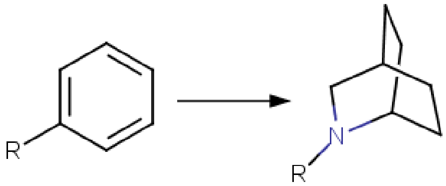 t, o, m, p	Ring substitution	[*:7]-[c;x2D3:1]1[c;x2:2][c;x2:6][c;x2:4][c;x2:5][c;x2:3]1>>[*:7]-[#7:1]-1-[#6:2]-[#6:6]-2-[#6:4]-[#6:5]-[#6:3]-1-[#6]-[#6]-2 |rb:1:2,2:2,3:2,4:2,5:2,6:2,s:1:3|	-Bioisosteric replacement	(Subbaiah and Meanwell, 2021)
Phenyl	Pheny_to_2_6_diazaspiro_3_3_heptane 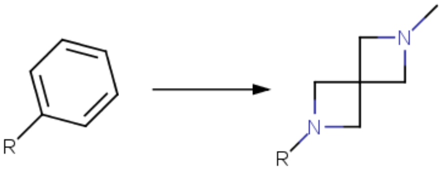	Ring substitution	[*:7]-[c;x2D3:1]1[c;x2:2][c;x2:3][c;x2:4][c;x2:5][c;x2:6]1>>[#6]-[#7:4]-1-[#6:3]C2([#6:5]-1)[#6:2]-[#7:1](-[*:7])-[#6:6]2 |rb:1:2,2:2,3:2,4:2,5:2,6:2,s:1:3,4:2|	-Bioisosteric replacement	(Subbaiah and Meanwell, 2021)
Phenyl	Pheny_to_2_6_diazaspiro_3_3_heptane 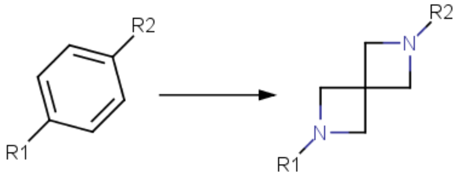	Ring substitution	[*:8]-[c;x2D3:4]1[c;x2:3][c;x2:2][c;x2D3:1](-[*:7])[c;x2:6][c;x2:5]1>>[*:8]-[#7:4]-1-[#6:3]C2([#6:5]-1)[#6:2]-[#7:1](-[*:7])-[#6:6]2 |rb:1:2,2:2,3:2,4:2,6:2,7:2,s:1:3,4:3|	-Bioisosteric replacement	(Subbaiah and Meanwell, 2021)
Phenyl	Phenyl_to_2_oxa_6_azaspiro_3_3_heptane 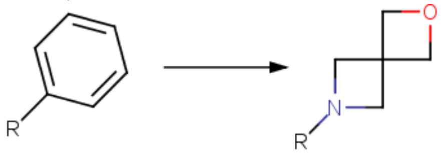 t, o, m	Ring substitution	[*:7]-[c;x2D3:1]1[c;x2:2][c;x2:3][c;x2D2:4][c;x2:5][c;x2:6]1>>[*:7]-[#7:1]-1-[#6:2]C2([#6:3]-[#8]-[#6:5]2)[#6]-1 |rb:1:2,2:2,3:2,4:2,5:2,6:2,s:1:3,4:2|	-Bioisosteric replacement	(Subbaiah and Meanwell, 2021)
Phenyl	Phenyl_to_1-(azetidin-1-yl)one 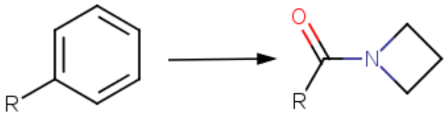 t, o, m	Ring substitution	[*:5]-[c;x2D3:1]1[c;x2:2][c;x2D2][c;D2][c;x2:4][c;x2:3]1>>[*:5]-[#6](=O)-[#7:1]-1-[#6:2]-[#6:4]-[#6:3]-1 |rb:1:2,2:2,3:2,4:2,5:2,6:2,s:1:3|	-Bioisostere replacement -Increased potency,	(Palacios et al., 2019; Subbaiah and Meanwell, 2021)
Phenyl	Phenyl_to_1-(azetidin-1-yl)one 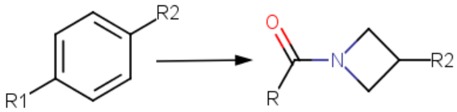	Ring substitution	[*:5]-[c;x2D3:1]1[c;x2:2][c;x2D2][c;x2:4](-[*:6])[c;x2D2][c;x2:3]1>>[*:6]-[#6:4]-1-[#6:2]-[#7:1](-[#6:3]-1)-[#6](-[*:5])=O |rb:1:2,2:2,3:2,4:2,6:2,7:2||	-Bioisostere replacement -Increased potency	(Palacios et al., 2019; Subbaiah and Meanwell, 2021)
Phenyl	Phenyl_to_n_propyl_oxime 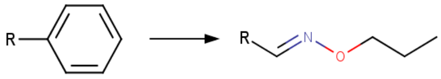 t	Functional group change	[*:3]-[c;x2D3:1]1[c;x2D2][c;x2D2][c;x2D2][c;x2D2][c;x2D2]1>>[#6]-[#6]-[#6]-[#8]\[#7]=[#6:1]\[*:3] |rb:1:2,2:2,3:2,4:2,5:2,6:2,s:1:3|	-Bioisostere replacement -Modulation of selectivity	(Piemontese et al., 2015; Subbaiah and Meanwell, 2021)

<table>
<tr><td>Phenyl</td><td>Phenyl_to_oxime_t
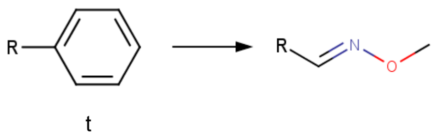</td><td>Functional group change</td><td>[*:3]-[c;x2D3:1]1[c;x2D2][c;x2D2][c;x2D2][c;x2D2][c;x2D2]1>>[#6]-[#8]\[#7]=[#6:1]\[*:3] |rb:1:2,2:2,3:2,4:2,5:2,6:2,s:1:3|</td><td>-Isostere replacement</td><td>(Macchia et al., 1994; Subbaiah and Meanwell, 2021)</td></tr>
<tr><td>Phenyl</td><td>Phenyl_to_oxime_m
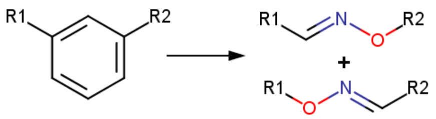</td><td>Linker modification</td><td>[*:2]-[c;x2D3:1]1[c;x2D2][c;x2D2][c;x2D2][c;x2D3:3](-[*:4])[c;x2D2]1>>[*:2]-[#6:1]-[#8]\[#7]=[#6:3]\[*:4] |rb:1:2,2:2,3:2,4:2,5:2,7:2,s:1:3,5:3|</td><td>-Isostere replacement</td><td>(Macchia et al., 1994; Subbaiah and Meanwell, 2021)</td></tr>
<tr><td>Phenyl</td><td>Phenyl_to_oxime_p
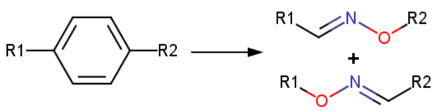</td><td>Linker modification</td><td>[*:2]-[c;x2D3:1]1[c;x2D2][c;x2D2][c;x2D3:3](-[*:4])[c;x2D2][c;x2D2]1>>[*:2]-[#6:1]-[#8]\[#7]=[#6:3]\[*:4] |rb:1:2,2:2,3:2,4:2,6:2,7:2,s:1:3,4:3|</td><td>-Isostere replacement</td><td>(Macchia et al., 1994; Subbaiah and Meanwell, 2021)</td></tr>
<tr><td>Phenyl</td><td>Phenyl_to_pinacolone_t
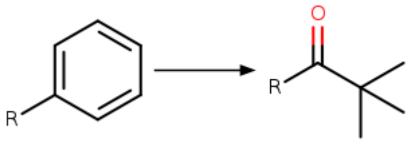</td><td>Functional group change</td><td>[*:2]-[c;x2D3:1]1[c;x2D2][c;x2D2][c;x2D2][c;x2D2][c;x2D2]1>>[#6]C([#6])([#6])[#6:1](-[*:2])=O |rb:1:2,2:2,3:2,4:2,5:2,6:2,s:1:3|</td><td>-Isosteric replacement

-Increased metabolic stability</td><td>(Subbaiah and Meanwell, 2021)</td></tr>
<tr><td>Phenyl</td><td>phenyl_to_pinacolone_o
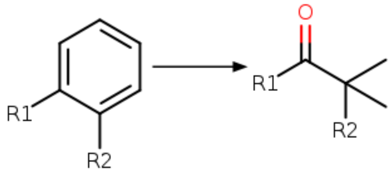</td><td>Linker modification</td><td>[*:4]-[c;x2D3:3]1[c;x2D2][c;x2D2][c;x2D2][c;x2D2][c;x2D3:1]1-[*:2]>>[#6][C:3]([#6])([*:4])[#6:1](-[*:2])=O |rb:1:2,2:2,3:2,4:2,5:2,6:2,s:1:3,6:3|</td><td>-Increased metabolic stability .Mitigated bioactivation and GSH adduct formation
-Improved oral bioavailability</td><td>(Wood et al., 2006; Subbaiah and Meanwell, 2021)</td></tr>
<tr><td>Phenyl</td><td>Phenyl_to_cyclopropyl carbonyl_t
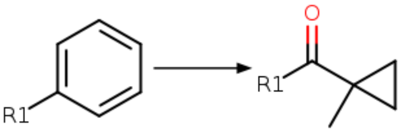</td><td>Functional group change</td><td>[*:2]-[c;x2D3:1]1[c;x2D2][c;x2D2][c;x2D2][c;x2D2][c;x2D3:3]1-[*:4]>>[*:2]-[#6:1](=O)[C:3]1([*:4])[#6]-[#6]1 |rb:1:2,2:2,3:2,4:2,5:2,6:2,s:1:3,6:3|</td><td>-Isosteric replacement

-Increased metabolic stability</td><td>(Subbaiah and Meanwell, 2021)</td></tr>
<tr><td>Phenyl</td><td>Phenyl_to_cyclopropyl carbonyl_o
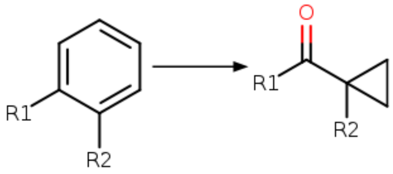</td><td>Linker modification</td><td>[*:4]-[c;x2D3:3]1[c;x2D2][c;x2D2][c;x2D2][c;x2D2][c;x2D3:1]1-[*:2]>>[*:2]-[#6:1](=O)[C:3]1([*:4])[#6]-[#6]1 |rb:1:2,2:2,3:2,4:2,5:2,6:2,s:1:3,6:3|</td><td>-Increased metabolic stability .Mitigated bioactivation and GSH adduct formation
-Improved oral bioavailability</td><td>(Wood et al., 2006; Subbaiah and Meanwell, 2021)</td></tr>
<tr><td>Phenyl</td><td>Phenyl_to_ciclopropyl_t
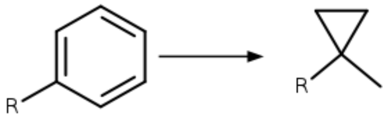</td><td>Functional group change.</td><td>[*:2]-[c;x2D3:1]1[c;x2D2][c;x2D2][c;x2D2][c;x2D2][c;x2D2]1>>[#6][C:1]1([*:2])[#6]-[#6]1 |rb:1:2,2:2,3:2,4:2,5:2,6:2,s:1:3|</td><td>-Reduced MW
-Llower lipophilicity
-Increased LLE
-Higher Fsp^3 count</td><td>(Subbaiah and Meanwell, 2021)</td></tr>
</table>

<table>
<tr><th></th><th></th><th></th><th></th><th></th><th></th></tr>
<tr><td>Phenyl</td><td>Phenyl_to_ciclopropyl_o
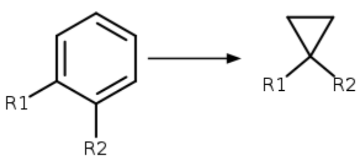</td><td>Ring substitution</td><td>[*:3]-[c;x2D3]1[c;x2D2][c;x2D2][c;x2D2][c;x2D2][c;x2D3:1]1-[*:2]>>[*:3][C:1]1([*:2])[#6]-[#6]1 |rb:1:2,2:2,3:2,4:2,5:2,6:2,s:1:3,6:3|</td><td>-Increased potency,
-Reduced MW
-Llower lipophilicity
-Increased LLE
-Higher Fsp^3 count</td><td>(Subbaiah and Meanwell, 2021)</td></tr>
<tr><td>Phenyl</td><td>Phenyl_to_OCCF3
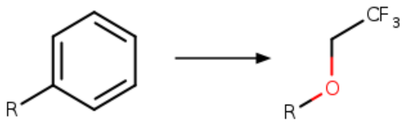
t</td><td>Functional group change.</td><td>[*:2]-[c;x2D3:1]1[c;x2D2][c;x2D2][c;x2D2][c;x2D2][c;x2D2]1>>FC(F)(F)[#6]-[#8]-[#6:1]-[*:2] |rb:1:2,2:2,3:2,4:2,5:2,6:2,s:1:3|</td><td>-Reduce phototoxicity risk
-Lower lipophilicity
-Enhanced Solubility
-Reduced PPB
-Reduced protein covalent binding
-Reduced hERG inhibition</td><td>(Subbaiah and Meanwell, 2021)</td></tr>
<tr><td>Phenyl</td><td>Phenyl_to_OCCCF3
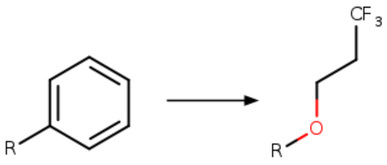
t</td><td>Functional group change.</td><td>[*:2]-[c;x2D3:1]1[c;x2D2][c;x2D2][c;x2D2][c;x2D2][c;x2D2]1>>FC(F)(F)[#6]-[#6]-[#8]-[#6:1]-[*:2] |rb:1:2,2:2,3:2,4:2,5:2,6:2,s:1:3|</td><td>-Reduce phototoxicity risk
-Lower lipophilicity
-Enhanced Solubility
-Reduced PPB
-Reduced protein covalent binding
-Reduced hERG inhibition</td><td>(Subbaiah and Meanwell, 2021)</td></tr>
<tr><td>Phenyl</td><td>Phenyl_to_acetylenic_t
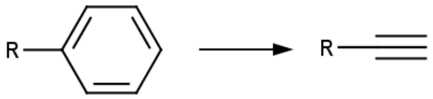</td><td>Functional group change.</td><td>[*:2]-[c;x2D3:1]1[c;x2D2][c;x2D2][c;x2D2][c;x2D2][c;x2D2]1>>[*:2][C:1]#C |rb:1:2,2:2,3:2,4:2,5:2,6:2|</td><td>-Isosteric replacement
-Reduced lipophilicity
-Improved metabolic stability
-Enhanced aqueous solubility</td><td>(Dragovich et al., 2003; Talele, 2020; Subbaiah and Meanwell, 2021)</td></tr>
<tr><td>Phenyl</td><td>Phenyl_to_acetylenic_p
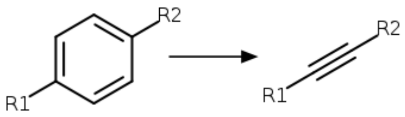</td><td>Functional group change/ Linker modification</td><td>[*:3]-[c;x2D3:2]1[c;x2D2][c;x2D2][c;x2D3:1](-[*:4])[c;x2D2][c;x2D2]1>>[#6:3][C:2]#[C:1][*:4] |rb:1:2,2:2,3:2,4:2,6:2,7:2,s:1:3,4:3|</td><td>-Isosteric replacement</td><td>(Talele, 2020; Subbaiah and Meanwell, 2021)</td></tr>
<tr><td>Phenyl</td><td>Phenyl_to_ethylen_t
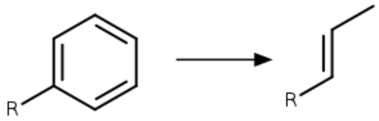</td><td>Functional group change</td><td>[*:2]-[c;x2D3:1]1[c;x2D2][c;x2D2][c;x2D2][c;x2D2][c;x2D2]1>>[#6]-[#6]=[#6:1]-[*:2] |rb:1:2,2:2,3:2,4:2,5:2,6:2,s:1:3|</td><td>-Bioisostere replacement

* Reactive structure</td><td>(Subbaiah and Meanwell, 2021)</td></tr>
<tr><td>Phenyl</td><td>Phenyl_to_ethylene_p
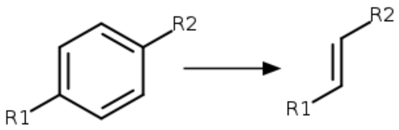</td><td>Functional group change/ Linker modification</td><td>[*:3]-[c;x2D3:2]1[c;x2D2][c;x2D2][c;x2D3:1](-[*:4])[c;x2D2][c;x2D2]1>>[*:3]\[#6:2]=[#6:1]\[*:4] |rb:1:2,2:2,3:2,4:2,6:2,7:2,s:1:3,4:3|</td><td>-Bioisostere replacement

* Reactive structure</td><td>(Morin et al., 2018; Subbaiah and Meanwell, 2021)</td></tr>
<tr><td>Phenyl</td><td>Phenyl_to_butanyl_t
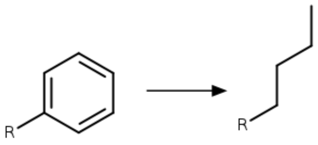</td><td>Functional group change</td><td>[*:2]-[c;x2D3:1]1[c;x2D2][c;x2D2][c;x2D2][c;x2D2][c;x2D2]1>>[#6]-[#6]-[#6]-[#6:1]-[*:2] |rb:1:2,2:2,3:2,4:2,5:2,6:2,s:1:3|</td><td>-Bioisostere replacement
-Increased Fsp^3 content
-Reduced $cLogD_{7.4}$
-Reduced MP</td><td>(Subbaiah and Meanwell, 2021)</td></tr>
</table>

Phenyl	Phenyl_to_butanyl_p 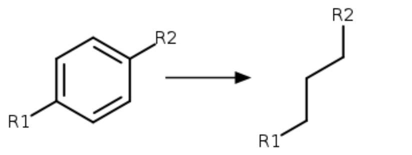	Functional group change/ Linker modification	[*:3]-[c;x2D3:2]1[c;x2D2][c;x2D2][c;x2D3:1](-[*:4])[c;x2D2][c;x2D2]1>>[#6]-[#6]-[#6]-[#6:1]-[*:2]	-Bioisostere replacement<br>-Increased Fsp^3 content<br>-Reduced cLogD$_{7.4}$<br>-Reduced MP<br>-Improved aqueous solubility	(Nicolaou et al., 2016; Subbaiah and Meanwell, 2021)
Phenyl	Phenyl_to_*trans*_acryl_m 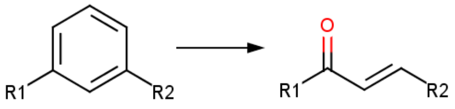	Functional group change/ Linker modification	[*:3]-[c;x2D3:2]1[c;x2D2][c;x2D2][c;x2D3:1](-[*:4])[c;x2D2][c;x2D2]1>>	-Bioisostere replacement<br><br>* Reactive structure	(Subbaiah and Meanwell, 2021)
Phenyl	Phenyl_to_trans-cyclopropyl_ketone_t 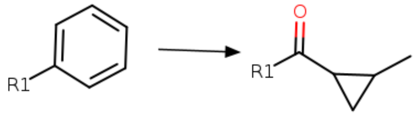	Functional group change	[#6:2]-[c;x2D3:1]1[c;x2D2][c;x2D2][c;x2D2][c;x2D2][c;x2D2]1>>[#6]-[#6]-1-[#6]-[#6]-1-[#6:1](-[*:2])=O |rb:1:2,2:2,3:2,4:2,5:2,6:2|	-Bioisostere replacement<br>-Reduced lipophilicity	(Kuhn et al., 2016; Subbaiah and Meanwell, 2021)
Phenyl	Phenyl_to_trans-cyclopropyl_ketone_m 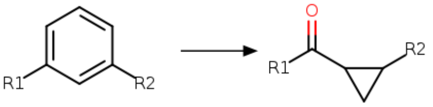	Linker modification	[*:2]-[c;x2D3:1]1[c;x2D2][c;x2D2][c;x2D2][c;x2D3:3](-[*:4])[c;x2D2]1>>[*:4]-[#6:3]-1-[#6]-[#6]-1-[#6:1](-[*:2])=O |rb:1:2,2:2,3:2,4:2,5:2,7:2|	-Bioisostere replacement<br>-Reduced lipophilicity<br>-Improved solubility	(Kuhn et al., 2016; Subbaiah and Meanwell, 2021)
Phenyl	Phenyl_to_trans-cyclopropyl_ketone_p 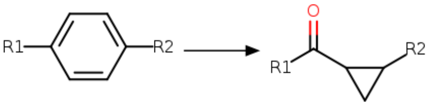	Linker modification	[*:3]-[c;x2D3:2]1[c;x2D2][c;x2D2][c;x2D3:1](-[*:4])[c;x2D2][c;x2D2]1>>[*:3]-[#6:2]-1-[#6]-[#6]-1-[#6:1](-[*:4])=O |rb:1:2,2:2,3:2,4:2,6:2,7:2,s:1:3,4:3|	-Biososteric replacement<br>-Reduced lipophilicity	(Kuhn et al., 2016; Subbaiah and Meanwell, 2021)
Phenyl	Phenyl_to_amide_t 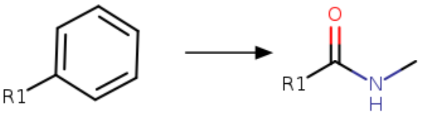	Functional group change	[*:2]-[c;x2D3:1]1[c;x2D2][c;x2D2][c;x2D2][c;x2D2][c;x2D2]1>>[#6]-[#7]-[#6:1](-[*:2])=O |rb:1:2,2:2,3:2,4:2,5:2,6:2|	-Biososteric replacement<br><br>-Reduced lipophilicity	(Subbaiah and Meanwell, 2021)
Phenyl	Phenyl_to_amide_m 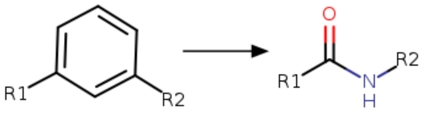	Linker modification	[*:2]-[c;x2D3:1]1[c;x2D2][c;x2D2][c;x2D2][c;x2D3:3](-[*:4])[c;x2D2]1>>[*:4]-[#7:3]-[#6:1](-[*:2])=O |rb:1:2,2:2,3:2,4:2,5:2,7:2|	-Biososteric replacement<br>-Preserved potency<br>-Reduced lipophilicity	(Beno et al., 2015; Subbaiah and Meanwell, 2021)
Phenyl	Phenyl_to_amide_p 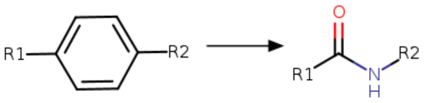	Linker modification	[*:3]-[c;x2D3:2]1[c;x2D2][c;x2D2][c;x2D3:1](-[*:4])[c;x2D2][c;x2D2]1>>[*:3]-[#7:2]-[#6:1](-[*:4])=O |rb:1:2,2:2,3:2,4:2,6:2,7:2,s:1:3,4:3|	-Biososteric replacement<br><br>-Reduced lipophilicity	(Subbaiah and Meanwell, 2021)

<table>
<tr><th></th><th></th><th></th><th></th><th></th><th></th></tr>
<tr><td>bipheyl</td><td>Biphenyl_to_thieno_3_2_b_thiophene_t_p
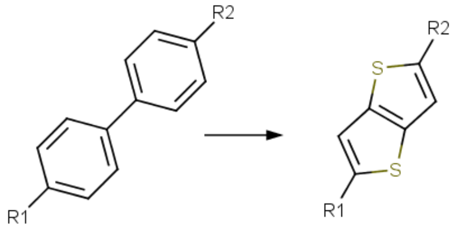</td><td>Ring substitution</td><td>[c;D3:1]1[c;D2:2][c;D2][c;D3]([c;D2][c;D2]1)-[c;D3]1[c;D2][c;D2][c;D3:4][c;D2:3][c;D2]1>>[#16:2]-1-[#6:1]=[#6]-[#6]-2=[#6]-1-[#6]=[#6:4]-[#16:3]-2 |c:14,16,19,s:0:3,1:2,9:3,10:2|</td><td>-Biososteric replacement</td><td>(Dousson et al., 2011; Subbaiah and Meanwell, 2021)</td></tr>
<tr><td>bipheyl</td><td>Biphenyl_to_thieno_3_2_b_thiophene2
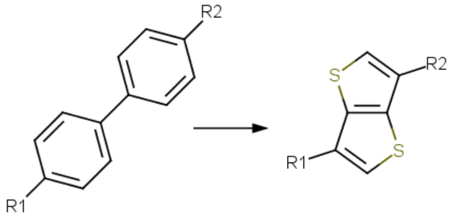</td><td>Ring substitution/ Linker modification</td><td>[c;D3:1]1[c;D2:2][c;D2][c;D3]([c;D2][c;D2]1)-[c;D3]1[c;D2][c;D2][c;D3:4][c;D2:3][c;D2]1>>[#16:2]-1-[#6]=[#6:1]-[#6]-2=[#6]-1-[#6:4]=[#6]-[#16:3]-2 |c:14,16,19,s:0:3,1:2,9:3,10:2|</td><td>-Biososteric replacement

-Improved potency</td><td>(Dousson et al., 2011; Subbaiah and Meanwell, 2021)</td></tr>
<tr><td>biphenyl</td><td>Bihenyl_to_acetylenic
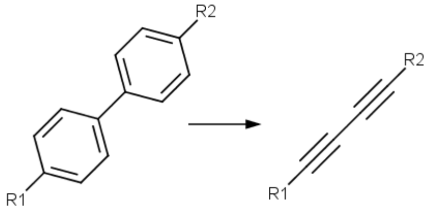</td><td>Functional group change/ Linker modification</td><td>[c;D3:1]1[c;D2][c;D2][c;D3]([c;D2][c;D2]1)-[c;D3]1[c;D2][c;D2][c;D3:2][c;D2][c;D2]1>>[C:1]#CC#[C:2] |s:0:3,9:3|</td><td>-Lower MW

-Reduced lipophilicity

-Improved PK</td><td>(Ivachtchenko et al., 2014)</td></tr>
<tr><td>biphenyl</td><td>biphenyl_to_biphenylene
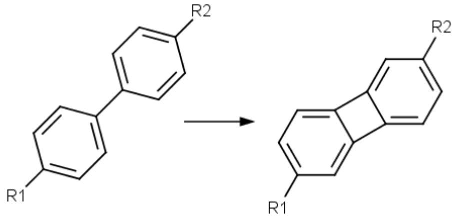</td><td>Ring addition/ Linker modification</td><td>[*:8]-[c;D3:7]1[c;D2][c;D2:6][c;D3:5]([c;D2][c;D2]1)-[c;D3:3]1[c;D2][c;D2][c;D3:2](-[*:1])[c;D2][c;D2:4]1>>[*:1]-[#6:2]-1=[#6]-[#6]=[#6:3]-2-[#6:4](=[#6]-1)-[#6:5]-1=[#6]-[#6]=[#6:7](-[*:8])-[#6]=[#6:6]-2-1 |t:16,25,s:1:3,10:3|</td><td>-Biososteric replacement

-Improved potency</td><td>(Kazmierski et al., 2020)</td></tr>
<tr><td>Sulfonamide</td><td>sulfonamide_to _gem-dimetilsulfone
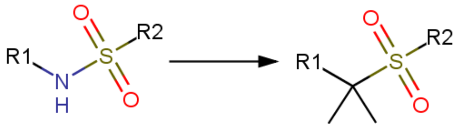</td><td>Linker modification</td><td>[*:1]-[#7;!R:2][S:3]([*:4])(=O)=O>>[#6][C:2]([#6])([*:1])[S:3]([*:4])(=O)=O</td><td>-Biososteric replacement

-Reduce hepatotoxicity risk</td><td>(Shao et al., 2013)</td></tr>
<tr><td>Sulfonamide</td><td>Sulfonamide_to_sulfonate_ester
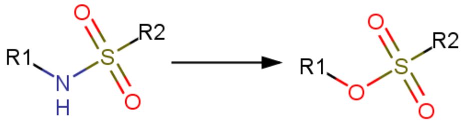</td><td>Linker modification</td><td>[*:5]-[#7;D2:4][S:2]([*:1])(=O)=[O:3]>>[*:5]-[#8:4][S:2]([*:1])(=[O:3])=O</td><td>-Biososteric replacement

-Reduce hepatotoxicity risk</td><td>(Vullo and Carta, 2019)</td></tr>
<tr><td>Sulfonamide</td><td>Sulfonamide_to_Sulfonimidamide
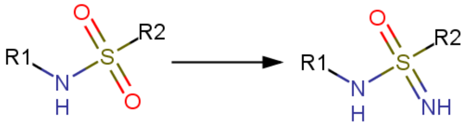</td><td>Linker modification</td><td>[*:5]-[#7;D2:4][S:2]([*:1])(=O)=[O:3]>>[*:5]-[#7:4][S:2]([*:1])(=[#7])=[O:3]</td><td>-Biososteric replacement
-Decreased lipophilicity and PPB
-Increased solubility</td><td>(Sehgelmeble et al., 2012)</td></tr>
</table>

Phosphate	Phosphate_to_sulphonate 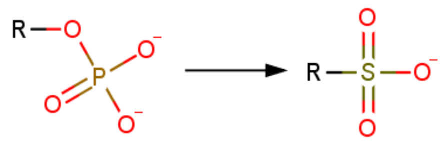	Functional group change	[#8;D1][P;X4:1]([#8;D1])(=[O;X1:2])[#8;X2]-[*:3]>>[#8-][S:1]([*:3])(=O)=[O;D1:2]	-Biososteric replacement	(Hussain et al., 2008)
Phosphate	Phosphate_to_acylsulphamate 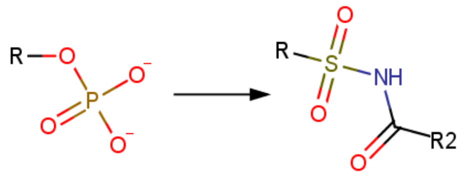	Functional group change	[#8;D1][P;X4:1]([#8;D1])(=[O;X1:2])[#8;X2]-[*:3]>>[#6]-[#6](=O)-[#7][S:1]([*:3])(=O)=[O;D1:2]<br><br>R2=CH3	-Biososteric replacement	(Hussain et al., 2008)
Phosphate	Phosphate_to_TZD 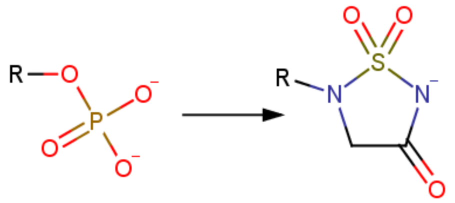	Ring addition	[#8;D1][P;X4:1]([#8;D1])(=[O;X1:2])[#8;X2:3]-[*:4]>>[*:4]-[#7:3]1-[#6]-[#6](=O)-[#7-][S:1]1(=[O:2])=O	-Biososteric replacement	(Elliott et al., 2012)
Phosphate	Phosphate_to_IZD 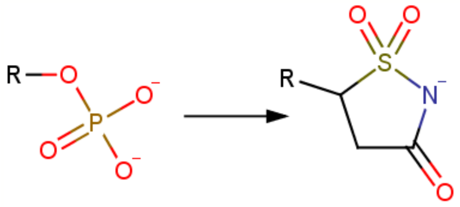	Ring addition	[#8;D1][P;X4:1]([#8;D1])(=[O;X1:2])[#8;X2:3]-[*:4]>>[*:4]-[#6:3]1-[#6]-[#6](=O)-[#7-][S:1]1(=[O:2])=O	-Biososteric replacement	(Elliott et al., 2012)
Phosphate	Phenyl_to_Benzoxazolinone 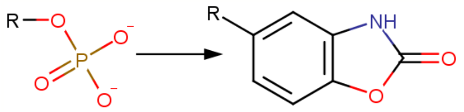	Ring addition	[#8;D1][P;X4:1]([#8;D1])(=[O;X1:2])[#8;X2]-[*:3]>>[*:3]-[#6]-1=[#6]-[#6]-2=[#6](-[#8]-[#6:1](=[O:2])-[#7]-2)-[#6]=[#6]-1	-Biososteric replacement	(Elliott et al., 2012)
Phosphate	Phenyl_to_Benzoxathiazole_dioxide 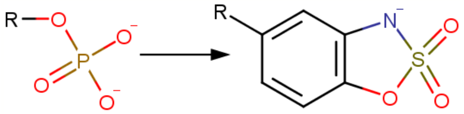	Ring addition	[#8;D1][P;X4:1]([#8;D1])(=[O;X1:2])[#8;X2]-[*:3]>>[*:3]-[#6]-1=[#6]-[#6]-2=[#6](-[#8][S:1](=[O:2])(=O)[#7-]-2)-[#6]=[#6]-1	-Biososteric replacement	(Elliott et al., 2012)
Phosphate	Phosphate_to_benzopyranone 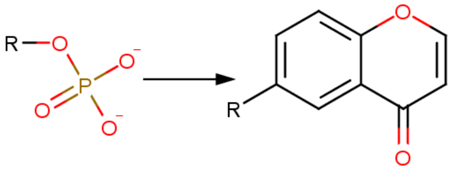	Ring addition	[#8;D1][P;X4:1]([#8;D1])(=[O;X1:2])[#8;X2:3]-[*:4]>>[*:4]-[#6:3]-1=[#6]-[#6]=[#6]-2-[#8]-[#6]=[#6]-[#6:1](=[O:2])-[#6]-2=[#6]-1	-Biososteric replacement	(Elliott et al., 2012)

Phosphate	Phosphate_to_thiochromenone<br>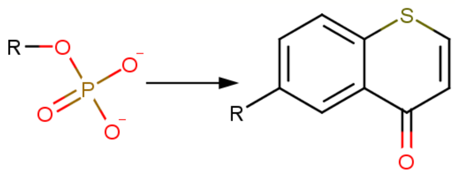	Ring addition	[#8;D1][P;X4:1]([#8;D1])(=[O;X1:2])[#8;X2:3]-[*:4]>>[*:4]-[#6:3]-1=[#6]-[#6]=[#6]-2-[#16]-[#6]=[#6]-[#6:1](=[O:2])-[#6]-2=[#6]-1	-Biososteric replacement	(Elliott et al., 2012)
Phosphate	Phosphate_to_dimethylnaphthalenone<br>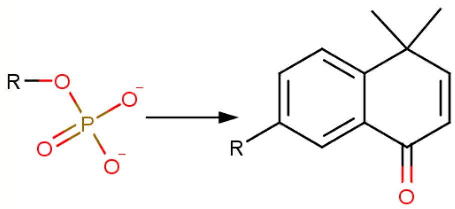	Ring addition	[#8;D1][P;X4:1]([#8;D1])(=[O;X1:2])[#8;X2:3]-[*:4]>>[#6]C1([#6])[#6]=[#6]-[#6:1](=[O:2])-[#6]-2=[#6]-[#6:3](-[*:4])=[#6]-[#6]=[#6]1-2	-Biososteric replacement	(Elliott et al., 2012)
Phosphate	Phosphate_to_benzoxathiine-4,4-dione<br>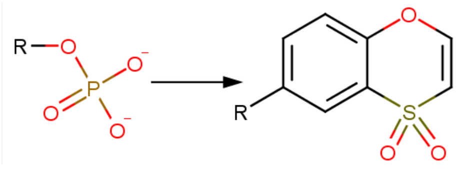	Ring addition	[#8;D1][P;X4:1]([#8;D1])(=[O;X1:2])[#8;X2:3]-[*:4]>>[*:4]-[#6:3]-1=[#6]-[#6]=[#6]-2-[#8]-[#6]=[#6][S:1](=[O:2])(=O)[#6]-2=[#6]-1	-Biososteric replacement	(Elliott et al., 2012)
Phosphate	Phosphate_to_squarate<br>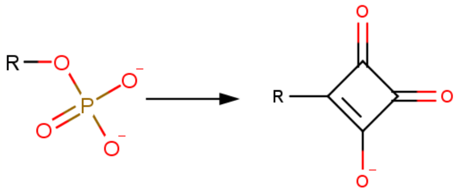	Ring addition	[#8;D1][P;X4:1]([#8;D1])(=[O;X1:2])[#8;X2:3]-[*:4]>>[#8D1-]-[#6]-1=[#6:3](-[*:4])-[#6:1](=[O:2])-[#6]-1=O	-Biososteric replacement	(Elliott et al., 2012)
Ester	Methyl/Ethyl_ester_to_1_3_4_oxadiazole<br>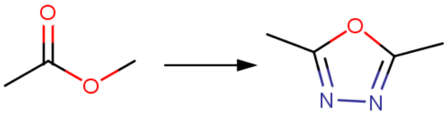	Ring addition	[#6;A;$([C;!R;H3:4]),$([C;!R;D2:4][C;D1]):4][#8:3]-[#6:2](-[#6:1])=O>>[#6:4]-[#6]-1=[#7]-[#7]=[#6:2](-[$([#1,*]):1])-[#8:3]-1	-Biososteric replacement<br><br>-Metabolic stability	(Hoshi et al., 2016)
Ester	Methyl/Ethyl_ester_to_1_2_4_oxadiazole<br>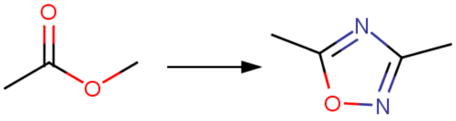	Ring addition	[#6;A;$([C;!R;H3:4]),$([C;!R;D2:4][C;D1]):4][#8:3]-[#6;!R:2](-[#6:1])=O>>[#6:4]-[#6]-1=[#7]-[#8]-[#6:2](-[$([#1,*]):1])=[#7:3]-1	-Biososteric replacement<br><br>-Metabolic stability	(Boström et al., 2012; Hoshi et al., 2016; Aprile et al., 2021; Camci and Karali, 2023)
Ester	Methyl/Ethyl_ester_to_oxazolidone<br>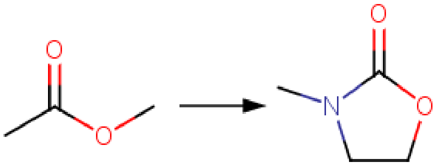	Ring addition	[#6;A;$([C;!R;H3:4]),$([C;!R;D2:4][C;D1])][#8:3]-[#6:2](-[#6:1])=O>>[$([#1,*]):1]-[#7]-1-[#6]-[#8]-[#6:3]-[#6:2]-1=O	-Biososteric replacement<br><br>-Metabolic stability	(Hoshi et al., 2016)
Ester	Methyl/Ethyl_ester_to_methoxyimidoyl_fluoride<br>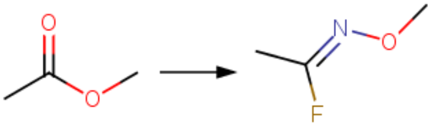	Functional group change	[#6;A;$([C;!R;H3:4]),$([C;!R;D2:4][C;D1]):4][#8:3]-[#6;!R:2](-[#6:1])=O>>F\[#6:2](-[*:1])=[#7]/[#8:3]-[*:4]	-Biososteric replacement<br><br>-Metabolic stability	(Hoshi et al., 2016)

Ester	Methyl/Ethyl_ester_to_methoxyimidoyl_chloride 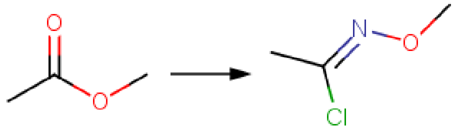	Functional group change	[#6;A;$([C;!R;H3:4]),$([C;!R;D2:4][C;D1]):4][#8:3]-[#6;!R:2](-[#6:1])=O>>Cl\[#6:2](-[*:1])=[#7]/[#8:3]-[*:4]	-Biososteric replacement<br>-Metabolic stability	(Hoshi et al., 2016)
Ester	Methyl/Ethyl_ester_to_cyanide 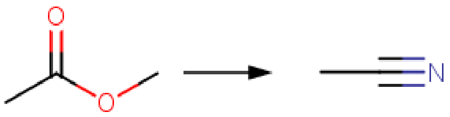	Functional group change	[#6;A;$([C;!R;H3:4]),$([C;!R;D2:4][C;D1])][#8:3]-[#6;!R:2](-[#6:1])=O>>[*:1][C:2]#[N:3]	-Biososteric replacement<br>-Metabolic stability	(Hoshi et al., 2016)
Ester	Methyl/Ethyl_ester_to_methyl_thioester 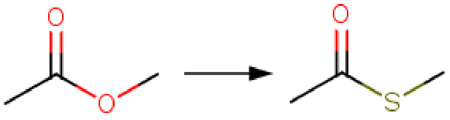	Atom modification	[#6;A;$([C;!R;H3:4]),$([C;!R;D2:4][C;D1]):4][#8:3]-[#6;!R:2](-[#6:1])=O>>[#6:1]-[#6;!R:2](=O)-[#16:3]-[*:4]	-Biososteric replacement<br>-Metabolic stability	(Hoshi et al., 2016)
Ester	Methyl/Ethyl_ester_to_methyl_thioester 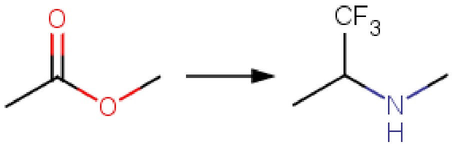	Functional group change	[#6;A;$([C;!R;H3:4]),$([C;!R;D2:4][C;D1])][#8:3]-[#6;!R:2](-[#6:1])=O>>[C:4][C;D1][#16:3]-[#6;!R:2](-[#6:1])=O	-Biososteric replacement<br>-Metabolic stability	(Hoshi et al., 2016)
Ester	Ester_to_trifluoro_propanamine 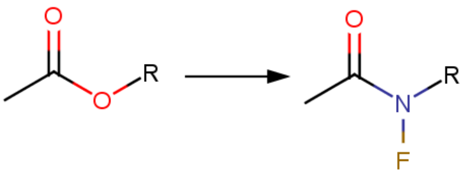	Functional group change	[#6:1]-[#6;!R:2](=O)-[#8:3]-[*:4]>>[#6:1]-[#6;!R:2](-[#7:3]-[*:4])C(F)(F)F	-Biososteric replacement<br>-Metabolic stability	(Hoshi et al., 2016)
Ester	Ester_to_amide 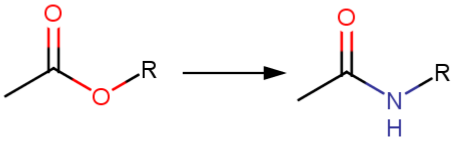	Atom modification	[#6:1]-[#6;!R:2](=O)-[#8:3]-[*:4]>>[#6:1]-[#6;!R:2](=O)-[#7:3]-[*:4]	-Biososteric replacement	(Swain, n.d.)
Ester	Ester_to_CNoxime 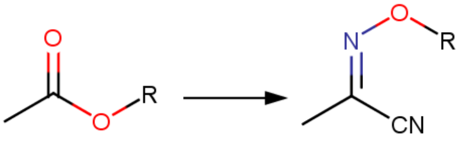	Functional group change	[#6:1]-[#6;!R:2](=O)-[#8:3]-[*:4]>>[#6:1]\[#6;!R:2](=[#7]/[#8:3]-[*:4])C#N	-Biososteric replacement<br>-Metabolic stability	(Swain, n.d.)
Ester	Ester_to_1_3_oxazole 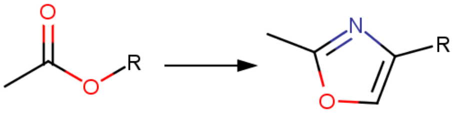	Ring addition	[#6:1]-[#6;!R:2](=O)-[#8:3]-[*:4]>>[*:1]-[#6:2]-1=[#7:3]-[#6](-[*:4])=[#6]-[#8]-1	-Biososteric replacement<br>-Metabolic stability	(Swain, n.d.)
Ester	Ester_to_1_2_4_triazole 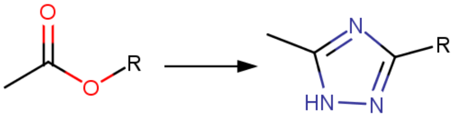	Ring addition	[#6:1]-[#6;!R:2](=O)-[#8:3]-[*:4]>>[*:1]-[#6:2]-1=[#7:3]-[#6](-[*:4])=[#7]-[#7]-1	-Biososteric replacement<br>-Metabolic stability	(Swain, n.d.)

ABBREVIATIONS USED:

BBB:blood brain barrier; Fsp^3 :fraction of sp^3 carbon centers ; hLiMT: human liver microtissues; LLE: ligand-lipophilicity efficiency; MP: melting point; MW: molecular weigh; PK:pharmacokinetic ; PL:phospholipidosis, PPB:plasma protein binding; TDI: CYP450 time-dependent inhibition.

REFERENCES:

Abe, H., Kikuchi, S., Hayakawa, K., Iida, T., Nagahashi, N., Maeda, K., et al. (2011). Discovery of a Highly Potent and Selective MEK Inhibitor: GSK1120212 (JTP-74057 DMSO Solvate). *ACS Med. Chem. Lett.* 2, 320–324.

Agnew-Francis, K. A., and Williams, C. M. (2020). Squaramides as Bioisosteres in Contemporary Drug Design. *Chem. Rev.* 120, 11616–11650.

Ahmed, S. K., Etoga, J.-L. G., Patel, S. A., Bridges, R. J., and Thompson, C. M. (2011). Use of the hydantoin isostere to produce inhibitors showing selectivity toward the vesicular glutamate transporter versus the obligate exchange transporter system xc-. *Bioorg. Med. Chem. Lett.* 21, 4358–4362.

Altman, R. A., Sharma, K. K., Rajewski, L. G., Toren, P. C., Baltezor, M. J., Pal, M., et al. (2018). Tyr1-ψ[(Z)CF=CH]-Gly2 Fluorinated Peptidomimetic Improves Distribution and Metabolism Properties of Leu-Enkephalin. *ACS Chem. Neurosci.* 9, 1735–1742.

Aprile, S., Riva, B., Bhela, I. P., Cordero-Sanchez, C., Avino, G., Genazzani, A. A., et al. (2021). 1,2,4-Oxadiazole-Bearing Pyrazoles as Metabolically Stable Modulators of Store-Operated Calcium Entry. *ACS Med. Chem. Lett.* 12, 640–646.

Ballatore, C., Gay, B., Huang, L., Robinson, K. H., James, M. J., Trojanowski, J. Q., et al. (2014). Evaluation of the cyclopentane-1,2-dione as a potential bio-isostere of the carboxylic acid functional group. *Bioorg. Med. Chem. Lett.* 24, 4171–4175.

Ballatore, C., Huryn, D. M., and Smith, A. B., 3rd (2013). Carboxylic acid (bio)isosteres in drug design. *ChemMedChem* 8, 385–395.

Ballatore, C., Soper, J. H., Piscitelli, F., James, M., Huang, L., Atasoylu, O., et al. (2011). Cyclopentane-1,3-dione: a novel isostere for the carboxylic acid functional group. Application to the design of potent thromboxane (A2) receptor antagonists. *J. Med. Chem.* 54, 6969–6983.

Barnes-Seeman, D., Jain, M., Bell, L., Ferreira, S., Cohen, S., Chen, X.-H., et al. (2013). Metabolically Stable tert-Butyl Replacement. *ACS Med. Chem. Lett.* 4, 514–516.

Barrett, S. D., Bridges, A. J., Dudley, D. T., Saltiel, A. R., Fergus, J. H., Flamme, C. M., et al. (2008). The discovery of the benzhydroxamate MEK inhibitors CI-1040 and PD 0325901. *Bioorg. Med. Chem. Lett.* 18, 6501–6504.

Benmansour, F., Eydoux, C., Querat, G., de Lamballerie, X., Canard, B., Alvarez, K., et al. (2016). Novel 2-phenyl-5-[(E)-2-(thiophen-2-yl)ethenyl]-1,3,4-oxadiazole and 3-phenyl-5-[(E)-2-(thiophen-2-yl)ethenyl]-1,2,4-oxadiazole derivatives as dengue virus inhibitors targeting NS5 polymerase. *Eur. J. Med. Chem.* 109, 146–156.

Beno, B. R., Yeung, K.-S., Bartberger, M. D., Pennington, L. D., and Meanwell, N. A. (2015). A Survey of the Role of Noncovalent Sulfur Interactions in Drug Design. *J. Med. Chem.* 58, 4383–4438.

Bilello, J. P., Lallos, L. B., McCarville, J. F., La Colla, M., Serra, I., Chapron, C., et al. (2014). In vitro activity and resistance profile of samatasvir, a novel NS5A replication inhibitor of hepatitis C virus. *Antimicrob. Agents Chemother.* 58, 4431–4442.

Black, W. C., Bayly, C. I., Davis, D. E., Desmarais, S., Falgueyret, J.-P., Léger, S., et al. (2005). Trifluoroethylamines as amide isosteres in inhibitors of cathepsin K. *Bioorg. Med. Chem. Lett.* 15, 4741–4744.

Boezio, A. A., Andrews, K., Boezio, C., Chu-Moyer, M., Copeland, K. W., DiMauro, E. F., et al. (2018). 1,2,4-Triazolsulfone: A novel isosteric replacement of acylsulfonamides in the context of NaV1.7 inhibition. *Bioorg. Med. Chem. Lett.* 28, 2103–2108.

Bonazzi, S., Goold, C. P., Gray, A., Thomsen, N. M., Nunez, J., Karki, R. G., et al. (2020). Discovery of a Brain-Penetrant ATP-Competitive Inhibitor of the Mechanistic Target of Rapamycin (mTOR) for CNS Disorders. *J. Med. Chem.* 63, 1068–1083.

Boström, J., Hogner, A., Llinàs, A., Wellner, E., and Plowright, A. T. (2012). Oxadiazoles in medicinal chemistry. *J. Med. Chem.* 55, 1817–1830.

Bredael, K., Geurs, S., Clarisse, D., De Bosscher, K., and D'hooghe, M. (2022). Carboxylic acid bioisosteres in medicinal chemistry: Synthesis and properties. *J. Chem.* 2022, 1–21.

Burkhard, J. A., Wuitschik, G., Rogers-Evans, M., Müller, K., and Carreira, E. M. (2010). Oxetanes as versatile elements in drug discovery and synthesis. *Angew. Chem. Int. Ed Engl.* 49, 9052–9067.

Camci, M., and Karali, N. (2023). Bioisosterism: 1,2,4-oxadiazole rings. *ChemMedChem* 18, e202200638.

Chang, W., Mosley, R. T., Bansal, S., Keilman, M., Lam, A. M., Furman, P. A., et al. (2012). Inhibition of hepatitis C virus NS5A by fluoro-olefin based γ-turn mimetics. *Bioorg. Med. Chem. Lett.* 22, 2938–2942.

Chebib, M., Johnston, G. A. R., and Mattsson, J. P. (1999). Aminomethyl-2, 6-difluorophenols as a novel class of increased lipophilicity GABAC receptor antagonists. *Bioorg. Med. Chem.* 9,3093-3098.

Cumming, J. N., Smith, E. M., Wang, L., Misiaszek, J., Durkin, J., Pan, J., et al. (2012). Structure based design of iminohydantoin BACE1 inhibitors: identification of an orally available, centrally active BACE1 inhibitor. *Bioorg. Med. Chem. Lett.* 22, 2444–2449.

Dalvie, D. K., Kalgutkar, A. S., Khojasteh-Bakht, S. C., Obach, R. S., and O'Donnell, J. P. (2002). Biotransformation reactions of five-membered aromatic heterocyclic rings. *Chem. Res. Toxicol.* 15, 269–299.

Deng, Q., Lim, Y.-H., Anand, R., Yu, Y., Kim, J.-H., Zhou, W., et al. (2015). Use of molecular modeling aided design to dial out hERG liability in adenosine A(2A) receptor antagonists. *Bioorg. Med. Chem. Lett.* 25, 2958–2962.

Di Grandi, M. J., Berger, D. M., Hopper, D. W., Zhang, C., Dutia, M., Dunnick, A. L., et al. (2009). Novel pyrazolopyrimidines as highly potent B-Raf inhibitors. *Bioorg. Med. Chem. Lett.* 19, 6957–6961.

Dossetter, A. G., Douglas, A., and O'Donnell, C. (2012). A matched molecular pair analysis of in vitro human microsomal metabolic stability measurements for heterocyclic replacements of di-substituted benzene containing compounds – identification of those isosteres more likely to have beneficial effects. *Med. Chem. Commun.* 3, 1164–1169.

Dousson, C. B., Dukhan, D., Parsy, C. C., and Pierra, C. (2011). Preparation of 5, 5-fused arylenes or heteroarylenes end-capped with amino acid and peptide derivatives as hepatitis C virus inhibitors. *World Patent Application*.

Dragovich, P. S., Prins, T. J., Zhou, R., Johnson, T. O., Hua, Y., Luu, H. T., et al. (2003). Structure-based design, synthesis, and biological evaluation of irreversible human rhinovirus 3C protease inhibitors. 8. Pharmacological optimization of orally bioavailable 2-pyridone-containing peptidomimetics. *J. Med. Chem.* 46, 4572–4585.

Dubois, M. A. J., Croft, R. A., Ding, Y., Choi, C., Owen, D. R., Bull, J. A., et al. (2021). Investigating 3,3-diaryloxetanes as potential bioisosteres through matched molecular pair analysis. *RSC Med Chem* 12, 2045–2052.

Dundee, J. W., Halliday, N. J., Harper, K. W., and Brogden, R. N. (1984). Midazolam. A review of its pharmacological properties and therapeutic use. *Drugs* 28, 519–543.

Elliott, T. S., Slowey, A., Ye, Y., and Conway, S. J. (2012). The use of phosphate bioisosteres in medicinal chemistry and chemical biology. *Medchemcomm* 3, 735–751.

Erickson, J. A., and McLoughlin, J. I. (1995). Hydrogen Bond Donor Properties of the Difluoromethyl Group. *J. Org. Chem.* 60, 1626–1631.

Evelyn, C. R., Bell, J. L., Ryu, J. G., Wade, S. M., Kocab, A., Harzdorf, N. L., et al. (2010). Design, synthesis and prostate cancer cell-based studies of analogs of the Rho/MKL1 transcriptional pathway inhibitor,

CCG-1423. *Bioorg. Med. Chem. Lett.* 20, 665–672.

Fransson, R., Nordvall, G., Bylund, J., Carlsson-Jonsson, A., Kratz, J. M., Svensson, R., et al. (2014). Exploration and Pharmacokinetic Profiling of Phenylalanine Based Carbamates as Novel Substance P 1–7 Analogues. *ACS Med. Chem. Lett.* 5, 1272–1277.

Froestl, W., Mickel, S. J., Hall, R. G., von Sprecher, G., Strub, D., Baumann, P. A., et al. (1995). Phosphinic Acid Analogs of GABA. 1. New Potent and Selective GABAB Agonists. *J. Med. Chem.* 38, 3297–3312.

Furet, P., Guagnano, V., Fairhurst, R. A., Imbach-Weese, P., Bruce, I., Knapp, M., et al. (2013). Discovery of NVP-BYL719 a potent and selective phosphatidylinositol-3 kinase alpha inhibitor selected for clinical evaluation. *Bioorg. Med. Chem. Lett.* 23, 3741–3748.

Hidalgo-Figueroa, S., Ramírez-Espinosa, J. J., Estrada-Soto, S., Almanza-Pérez, J. C., Román-Ramos, R., Alarcón-Aguilar, F. J., et al. (2013). Discovery of thiazolidine-2,4-dione/biphenylcarbonitrile hybrid as dual PPAR α/γ modulator with antidiabetic effect: in vitro, in silico and in vivo approaches. *Chem. Biol. Drug Des.* 81, 474–483.

Hoshi, A., Sakamoto, T., Takayama, J., Xuan, M., Okazaki, M., Hartman, T. L., et al. (2016). Systematic evaluation of methyl ester bioisosteres in the context of developing alkenyldiarylmethanes (ADAMs) as non-nucleoside reverse transcriptase inhibitors (NNRTIs) for anti-HIV-1 chemotherapy. *Bioorg. Med. Chem.* 24, 3006–3022.

Hübner, H., Haubmann, C., Utz, W., and Gmeiner, P. (2000). Conjugated Enynes as Nonaromatic Catechol Bioisosteres: Synthesis, Binding Experiments, and Computational Studies of Novel Dopamine Receptor Agonists Recognizing Preferentially the D3 Subtype. *J. Med. Chem.* 43, 756–762.

Hussain, M., Ahmed, V., Hill, B., Ahmed, Z., and Taylor, S. D. (2008). A re-examination of the difluoromethylenesulfonic acid group as a phosphotyrosine mimic for PTP1B inhibition. *Bioorg. Med. Chem.* 16, 6764–6777.

Isabel, E., Mellon, C., Boyd, M. J., Chauret, N., Deschênes, D., Desmarais, S., et al. (2011). Difluoroethylamines as an amide isostere in inhibitors of cathepsin K. *Bioorg. Med. Chem. Lett.* 21, 920–923.

Ivachtchenko, A. V., Mitkin, O. D., Yamanushkin, P. M., Kuznetsova, I. V., Bulanova, E. A., Shevkun, N. A., et al. (2014). Discovery of novel highly potent hepatitis C virus NS5A inhibitor (AV4025). *J. Med. Chem.* 57, 7716–7730.

Iwaki, Y., Ohhata, A., Nakatani, S., Hisaichi, K., Okabe, Y., Hiramatsu, A., et al. (2020). ONO-8430506: A Novel Autotaxin Inhibitor That Enhances the Antitumor Effect of Paclitaxel in a Breast Cancer Model. *ACS Med. Chem. Lett.* 11, 1335–1341.

Jagtap, A. D., Kondekar, N. B., Sadani, A. A., and Chern, J.-W. (2017). Ureas: Applications in Drug Design. *Curr. Med. Chem.* 24, 622–651.

Kazmierski, W. M., Baskaran, S., Walker, J. T., Miriyala, N., Meesala, R., Beesu, M., et al. (2020). GSK2818713, a Novel Biphenylene Scaffold-Based Hepatitis C NS5A Replication Complex Inhibitor with Broad Genotype Coverage. *J. Med. Chem.* 63, 4155–4170.

Kiesman, W. F., Zhao, J., Conlon, P. R., Dowling, J. E., Petter, R. C., Lutterodt, F., et al. (2006). Potent and orally bioavailable 8-bicyclo[2.2.2]octylxanthines as adenosine A1 receptor antagonists. *J. Med. Chem.* 49, 7119–7131.

Kim, J. J., Wood, M. R., Stachel, S. J., de Leon, P., Nomland, A., Stump, C. A., et al. (2014). (E)-Alkenes as replacements of amide bonds: development of novel and potent acyclic CGRP receptor antagonists. *Bioorg. Med. Chem. Lett.* 24, 258–261.

Kotoku, M., Maeba, T., Fujioka, S., Yokota, M., Seki, N., Ito, K., et al. (2019). Discovery of Second Generation RORγ Inhibitors Composed of an Azole Scaffold. *J. Med. Chem.* 62, 2837–2842.

Kuhn, B., Guba, W., Hert, J., Banner, D., Bissantz, C., Ceccarelli, S., et al. (2016). A real-world perspective on molecular design. *J. Med. Chem.* 59, 4087–4102.

Kumari, S., Carmona, A. V., Tiwari, A. K., and Trippier, P. C. (2020). Amide Bond Bioisosteres: Strategies, Synthesis, and Successes. *J. Med. Chem.* 63, 12290–12358.

Lassalas, P., Oukoloff, K., Makani, V., James, M., Tran, V., Yao, Y., et al. (2017). Evaluation of Oxetan-3-ol, Thietan-3-ol, and Derivatives Thereof as Bioisosteres of the Carboxylic Acid Functional Group. *ACS Med.*

Chem. Lett. 8, 864–868.

Levterov, V. V., Panasyuk, Y., Pivnytska, V. O., and Mykhailiuk, P. K. (2020). Water-soluble non-classical benzene mimetics. *Angew. Chem. Weinheim Bergstr. Ger.* 132, 7228–7234.

Liang, J., Zhang, B., Labadie, S., and Ortwine, D. F. (2016). Lead optimization of a pyrazolo [1, 5-a] pyrimidin-7 (4H)-one scaffold to identify potent, selective and orally bioavailable KDM5 inhibitors suitable for in vivo …. *Bioorg. Med. Chem.* 26, 4036-4041.

Li, J. J. (2020). *Medicinal Chemistry for Practitioners*. John Wiley & Sons.

Lu, D., and Vince, R. (2007). Discovery of potent HIV-1 protease inhibitors incorporating sulfoximine functionality. *Bioorg. Med. Chem. Lett.* 17, 5614–5619.

Ma, B., Bohnert, T., Otipoby, K. L., Tien, E., Arefayene, M., Bai, J., et al. (2020). Discovery of BIIB068: A Selective, Potent, Reversible Bruton's Tyrosine Kinase Inhibitor as an Orally Efficacious Agent for Autoimmune Diseases. *J. Med. Chem.* 63, 12526–12541.

Macchia, B., Balsamo, A., Breschi, M. C., Chiellini, G., Macchia, M., Martinelli, A., et al. (1994). The [(methyloxy)imino]methyl moiety as a bioisoster of aryl. A novel class of completely aliphatic beta-adrenergic receptor antagonists. *J. Med. Chem.* 37, 1518–1525.

Mattei, P., Boehringer, M., Di Giorgio, P., Fischer, H., Hennig, M., Huwyler, J., et al. (2010). Discovery of carmegliptin: a potent and long-acting dipeptidyl peptidase IV inhibitor for the treatment of type 2 diabetes. *Bioorg. Med. Chem. Lett.* 20, 1109–1113.

McKerrall, S. J., Nguyen, T., Lai, K. W., Bergeron, P., Deng, L., DiPasquale, A., et al. (2019). Structure- and Ligand-Based Discovery of Chromane Arylsulfonamide Nav1.7 Inhibitors for the Treatment of Chronic Pain. *J. Med. Chem.* 62, 4091–4109.

Meanwell, N. A. (2018). Fluorine and Fluorinated Motifs in the Design and Application of Bioisosteres for Drug Design. *J. Med. Chem.* 61, 5822–5880.

Meanwell, N. A., and Sistla, R. (2021). "Chapter Two - A survey of applications of tetrahydropyrrolo-3,4-azoles and tetrahydropyrrolo-2,3-azoles in medicinal chemistry," in *Advances in Heterocyclic Chemistry*, eds. N. A. Meanwell and M. L. Lolli (Academic Press), 31–100.

Mittal, R., and Awasthi, S. K. (2019). Recent Advances in the Synthesis of 5-Substituted 1H-Tetrazoles: A Complete Survey (2013–2018). *Synthesis* 51, 3765–3783.

Morin, M. D., Wang, Y., Jones, B. T., Mifune, Y., Su, L., Shi, H., et al. (2018). Diprovocims: A New and Exceptionally Potent Class of Toll-like Receptor Agonists. *J. Am. Chem. Soc.* 140, 14440–14454.

Mukherjee, P., Pettersson, M., Dutra, J. K., Xie, L., and Am Ende, C. W. (2017). Trifluoromethyl Oxetanes: Synthesis and Evaluation as a tert-Butyl Isostere. *ChemMedChem* 12, 1574–1577.

Mykhailiuk, P., Levterov, V., Panasyuk, Y., Sahun, K., Stashkevich, O., Badlo, V., et al. (2023). An "Ideal" Bioisoster of the para-substituted Phenyl Ring. *ChemRxiv*. doi: 10.26434/chemrxiv-2023-rbgz3.

Nakajima, K., Chatelain, R., Clairmont, K. B., Commerford, R., Coppola, G. M., Daniels, T., et al. (2017). Discovery of an Orally Bioavailable Benzimidazole Diacylglycerol Acyltransferase 1 (DGAT1) Inhibitor That Suppresses Body Weight Gain in Diet-Induced Obese Dogs and Postprandial Triglycerides in Humans. *J. Med. Chem.* 60, 4657–4664.

Navarrete-Vázquez, G., Alaniz-Palacios, A., Hidalgo-Figueroa, S., González-Acevedo, C., Ávila-Villarreal, G., Estrada-Soto, S., et al. (2013). Discovery, synthesis and in combo studies of a tetrazole analogue of clofibric acid as a potent hypoglycemic agent. *Bioorg. Med. Chem. Lett.* 23, 3244–3247.

Navarrete-Vázquez, G., Rojano-Vilchis, M. de M., Yépez-Mulia, L., Meléndez, V., Gerena, L., Hernández-Campos, A., et al. (2006). Synthesis and antiprotozoal activity of some 2-(trifluoromethyl)-1H-benzimidazole bioisosteres. *Eur. J. Med. Chem.* 41, 135–141.

Nicolaou, I., Zika, C., and Demopoulos, V. J. (2004). [1-(3,5-difluoro-4-hydroxyphenyl)-1H-pyrrol-3-yl]phenylmethanone as a bioisostere of a carboxylic acid aldose reductase inhibitor. *J. Med. Chem.* 47, 2706–2709.

Nicolaou, K. C., Vourloumis, D., Totokotsopoulos, S., Papakyriakou, A., Karsunky, H., Fernando, H., et al. (2016). Synthesis and Biopharmaceutical Evaluation of Imatinib Analogues Featuring Unusual Structural

Motifs. *ChemMedChem* 11, 31–37.

Palacios, D. S., Meredith, E. L., Kawanami, T., Adams, C. M., Chen, X., Darsigny, V., et al. (2019). Scaffold Morphing Identifies 3-Pyridyl Azetidine Ureas as Inhibitors of Nicotinamide Phosphoribosyltransferase (NAMPT). *ACS Med. Chem. Lett.* 10, 1524–1529.

Peng, X., Lanter, J. C., Y-P Chen, A., Brand, M. A., Wozniak, M. K., Hoekman, S., et al. (2023). Discovery of oxazoline enhancers of cellular progranulin release. *Bioorg. Med. Chem. Lett.* 80, 129048.

Piemontese, L., Fracchiolla, G., Carrieri, A., Parente, M., Laghezza, A., Carbonara, G., et al. (2015). Design, synthesis and biological evaluation of a class of bioisosteric oximes of the novel dual peroxisome proliferator-activated receptor α/γ ligand LT175. *Eur. J. Med. Chem.* 90, 583–594.

Pippione, A. C., Dosio, F., Ducime, A., Federico, A., Martina, K., Sainas, S., et al. (2015). Substituted 4-hydroxy-1,2,3-triazoles: synthesis, characterization and first drug design applications through bioisosteric modulation and scaffold hopping approaches. *Med. Chem. Commun.* 6, 1285–1292.

Pirali, T., Serafini, M., Cargnin, S., and Genazzani, A. A. (2019). Applications of Deuterium in Medicinal Chemistry. *J. Med. Chem.* 62, 5276–5297.

Press, Neil J., Taylor, R. J., Fullerton, J., Tranter, P., McCarthy, C., Keller, T. H., et al. (2015). Discovery and Optimization of 4-(8-(3-Fluorophenyl)-1,7-naphthyridin-6-yl)transcyclohexanecarboxylic Acid, an Improved PDE4 Inhibitor for the Treatment of Chronic Obstructive Pulmonary Disease (COPD). *J. Med. Chem.* 58, 6747–6752.

Press, N. J., Taylor, R. J., Fullerton, J. D., Tranter, P., McCarthy, C., Keller, T. H., et al. (2012). Solubility-driven optimization of phosphodiesterase-4 inhibitors leading to a clinical candidate. *J. Med. Chem.* 55, 7472–7479.

Prysiazhniuk, K., Datsenko, O., Polishchuk, O., Shulha, S., Shablykin, O., Nikandrova, L., et al. (2023). Spiro[3.3]heptane as a Non-collinear Benzene Bioisostere. *ChemRxiv*. doi: 10.26434/chemrxiv-2023-djc05.

Randolph, J. T., Krueger, A. C., Donner, P. L., Pratt, J. K., Liu, D., Motter, C. E., et al. (2018). Synthesis and Biological Characterization of Aryl Uracil Inhibitors of Hepatitis C Virus NS5B Polymerase: Discovery of ABT-072, a trans-Stilbene Analog with Good Oral Bioavailability. *J. Med. Chem.* 61, 1153–1163.

Ratni, H., Baumann, K., Bellotti, P., Cook, X. A., Green, L. G., Luebbers, T., et al. (2021). Phenyl bioisosteres in medicinal chemistry: discovery of novel γ-secretase modulators as a potential treatment for Alzheimer's disease. *RSC Med Chem* 12, 758–766.

Ripa, L., Edman, K., Dearman, M., Edenro, G., Hendrickx, R., Ullah, V., et al. (2018). Discovery of a Novel Oral Glucocorticoid Receptor Modulator (AZD9567) with Improved Side Effect Profile. *J. Med. Chem.* 61, 1785–1799.

Ritchie, T. J., and Macdonald, S. J. F. (2016). Heterocyclic replacements for benzene: Maximising ADME benefits by considering individual ring isomers. *Eur. J. Med. Chem.* 124, 1057–1068.

Rojas, J. J., Croft, R. A., Sterling, A. J., Briggs, E. L., Antermite, D., Schmitt, D. C., et al. (2022). Amino-oxetanes as amide isosteres by an alternative defluorosulfonylative coupling of sulfonyl fluorides. *Nat. Chem.* 14, 160–169.

Rombouts, F. J. R., Tovar, F., Austin, N., Tresadern, G., and Trabanco, A. A. (2015). Benzazaborinines as Novel Bioisosteric Replacements of Naphthalene: Propranolol as an Example. *J. Med. Chem.* 58, 9287–9295.

Schnider, P., Bissantz, C., Bruns, A., Dolente, C., Goetschi, E., Jakob-Roetne, R., et al. (2020). Discovery of Balovaptan, a Vasopressin 1a Receptor Antagonist for the Treatment of Autism Spectrum Disorder. *J. Med. Chem.* 63, 1511–1525.

Sehgelmeble, F., Janson, J., Ray, C., Rosqvist, S., Gustavsson, S., Nilsson, L. I., et al. (2012). Sulfonimidamides as sulfonamides bioisosteres: rational evaluation through synthetic, in vitro, and in vivo studies with γ-secretase inhibitors. *ChemMedChem* 7, 396–399.

Shao, P. P., Ye, F., Chakravarty, P. K., Herrington, J. B., Dai, G., Bugianesi, R. M., et al. (2013). Improved Cav2.2 Channel Inhibitors through a gem-Dimethylsulfone Bioisostere Replacement of a Labile Sulfonamide. *ACS Med. Chem. Lett.* 4, 1064–1068.

Sheppard, G. S., Pireh, D., Carrera, G. M., Jr, Bures, M. G., Heyman, H. R., Steinman, D. H., et al. (1994). 3-(2-(3-Pyridinyl)thiazolidin-4-oyl)indoles, a novel series of platelet activating factor antagonists. *J. Med. Chem.* 37, 2011–2032.

Shi, J., Gu, Z., Jurica, E. A., Wu, X., Haque, L. E., Williams, K. N., et al. (2018). Discovery of Potent and Orally Bioavailable Dihydropyrazole GPR40 Agonists. *J. Med. Chem.* 61, 681–694.

Sodano, T. M., Combee, L. A., and Stephenson, C. R. J. (2020). Recent Advances and Outlook for the Isosteric Replacement of Anilines. *ACS Med. Chem. Lett.* 11, 1785–1788.

Subbaiah, M. A. M., and Meanwell, N. A. (2021). Bioisosteres of the Phenyl Ring: Recent Strategic Applications in Lead Optimization and Drug Design. *J. Med. Chem.* 64, 14046–14128.

Sundriyal, S., Viswanad, B., Ramarao, P., Chakraborti, A. K., and Bharatam, P. V. (2008). New PPARγ ligands based on barbituric acid: Virtual screening, synthesis and receptor binding studies. *Bioorg. Med. Chem. Lett.* 18, 4959–4962.

Swain, C. (n.d.). Ester and amide bioisosteres. *Cambridge MedChem Consulting*. Available at: https://www.cambridgemedchemconsulting.com/resources/bioisoteres/ester_bioisosteres.html [Accessed March 29, 2023a].

Swain, C. (n.d.). Nitro bioisosteres. *Cambridge MedChem Consulting*. Available at: https://www.cambridgemedchemconsulting.com/news/index_files/e257c4796cad57a277e5b735ea47bf96-136.html [Accessed January 24, 2023b].

Swidorski, J. J., Jenkins, S., Hanumegowda, U., Parker, D. D., Beno, B. R., Protack, T., et al. (2021). Design and exploration of C-3 benzoic acid bioisosteres and alkyl replacements in the context of GSK3532795 (BMS-955176) that exhibit broad spectrum HIV-1 maturation inhibition. *Bioorg. Med. Chem. Lett.* 36, 127823.

Talele, T. T. (2020). Acetylene Group, Friend or Foe in Medicinal Chemistry. *J. Med. Chem.* 63, 5625–5663.

Tamura, Y., Omori, N., Kouyama, N., Nishiura, Y., Hayashi, K., Watanabe, K., et al. (2012). Design, synthesis and identification of novel benzimidazole derivatives as highly potent NPY Y5 receptor antagonists with attractive in vitro ADME profiles. *Bioorg. Med. Chem. Lett.* 22, 5498–5502.

Tseng, C.-C., Baillie, G., Donvito, G., Mustafa, M. A., Juola, S. E., Zanato, C., et al. (2019). The Trifluoromethyl Group as a Bioisosteric Replacement of the Aliphatic Nitro Group in CB1 Receptor Positive Allosteric Modulators. *J. Med. Chem.* 62, 5049–5062.

Vullo, D., and Carta, F. (2019). “Chapter 9 - Mechanisms of action of carbonic anhydrase inhibitors: zinc binders,” in *Carbonic Anhydrases*, eds. C. T. Supuran and A. Nocentini (Academic Press), 187–222.

Wager, T. T., Pettersen, B. A., Schmidt, A. W., Spracklin, D. K., Mente, S., Butler, T. W., et al. (2011). Discovery of two clinical histamine H(3) receptor antagonists: trans-N-ethyl-3-fluoro-3-[3-fluoro-4-(pyrrolidinylmethyl)phenyl]cyclobutanecarboxamide (PF-03654746) and trans-3-fluoro-3-[3-fluoro-4-(pyrrolidin-1-ylmethyl)phenyl]-N-(2-methylpropyl)cyclobutanecarboxamide (PF-03654764). *J. Med. Chem.* 54, 7602–7620.

Wang, L., Doherty, G. A., Judd, A. S., Tao, Z.-F., Hansen, T. M., Frey, R. R., et al. (2020). Discovery of A-1331852, a First-in-Class, Potent, and Orally-Bioavailable BCL-XL Inhibitor. *ACS Med. Chem. Lett.* 11, 1829–1836.

Wang, Y., Zhao, H., Brewer, J. T., Li, H., Lao, Y., Amberg, W., et al. (2018). De Novo Design, Synthesis, and Biological Evaluation of 3,4-Disubstituted Pyrrolidine Sulfonamides as Potent and Selective Glycine Transporter 1 Competitive Inhibitors. *J. Med. Chem.* 61, 7486–7502.

Winneroski, L. L., Erickson, J. A., Green, S. J., Lopez, J. E., Stout, S. L., Porter, W. J., et al. (2020). Preparation and biological evaluation of BACE1 inhibitors: Leveraging trans-cyclopropyl moieties as ligand efficient conformational constraints. *Bioorg. Med. Chem.* 28, 115194.

Winters, M. P., Crysler, C., Subasinghe, N., Ryan, D., Leong, L., Zhao, S., et al. (2008). Carboxylic acid bioisosteres acylsulfonamides, acylsulfamides, and sulfonylureas as novel antagonists of the CXCR2 receptor. *Bioorg. Med. Chem. Lett.* 18, 1926–1930.

Wood, M. R., Schirripa, K. M., Kim, J. J., Wan, B.-L., Murphy, K. L., Ransom, R. W., et al. (2006). Cyclopropylamino acid amide as a pharmacophoric replacement for 2,3-diaminopyridine. Application to the design of novel bradykinin B1 receptor antagonists. *J. Med. Chem.* 49, 1231–1234.

Wright, J. L., Gregory, T. F., Kesten, S. R., Boxer, P. A., Serpa, K. A., Meltzer, L. T., et al. (2000). Subtype-selective N-methyl-D-aspartate receptor antagonists: synthesis and biological evaluation of 1-(heteroarylalkynyl)-4-benzylpiperidines. *J. Med. Chem.* 43, 3408–3419.

Wuitschik, G., Carreira, E. M., Wagner, B., Fischer, H., Parrilla, I., Schuler, F., et al. (2010). Oxetanes in drug discovery: structural and synthetic insights. *J. Med. Chem.* 53, 3227–3246.

Xu, R., Li, S., Paruchova, J., McBriar, M. D., Guzik, H., Palani, A., et al. (2006). Bicyclic[4.1.0]heptanes as phenyl replacements for melanin concentrating hormone receptor antagonists. *Bioorg. Med. Chem.* 14, 3285–3299.

Xu, Y., Qian, L., Pontsler, A. V., McIntyre, T. M., and Prestwich, G. D. (2004). Synthesis of difluoromethyl substituted lysophosphatidic acid analogues. *Tetrahedron* 60, 43–49.

Zhang, C., Crawford, J. J., Landry, M. L., Chen, H., Kenny, J. R., Khojasteh, S. C., et al. (2020). Strategies to Mitigate the Bioactivation of Aryl Amines. *Chem. Res. Toxicol.* 33, 1950–1959.

Zhang, Y., and Pike, A. (2021). Pyridones in drug discovery: Recent advances. *Bioorg. Med. Chem. Lett.* 38, 127849.

Zhao, J.-X., Chang, Y.-X., He, C., Burke, B. J., Collins, M. R., Bel, M. D., et al. (2021). 1,2-Difunctionalized bicyclo[1.1.1]pentanes: Long–sought-after mimetics for ortho/meta-substituted arenes. *Proceedings of the National Academy of Sciences* 118, e2108881118.

Zhao, P., Nettleton, D. O., Karki, R. G., Zécri, F. J., and Liu, S.-Y. (2017). Medicinal Chemistry Profiling of Monocyclic 1,2-Azaborines. *ChemMedChem* 12, 358–361.
